# YAP1 induces hepatocellular carcinoma via DNA demethylation rather than by canonical driver gene mutations

**DOI:** 10.1038/s42003-026-10882-w

**Published:** 2026-09-17

**Authors:** Misaki Kosaka, Yoshimi Okamoto-Uchida, Haruka Hirose, Yuya Nagaoka, Norio Miyamura, Akinori Kanai, Daiki Hatakeyama, Michiko Nakagawa, Miki Nishio, Tomohiko Maehama, Akira Suzuki, Yutaka Suzuki, Teppei Shimamura, Hiroshi Nishina

**Affiliations:** 1 Medical Research Laboratory, Institute of Integrated Research, Institute of Science Tokyo, Tokyo, Japan; 2 Laboratory of Computational Life Science, National Cancer Center Research Institute, Tokyo, Japan; 3 Graduate School of Frontier Sciences, The University of Tokyo, Chiba, Japan; 4 Division of Molecular and Cellular Biology, Kobe University Graduate School of Medicine, Kobe, Japan; 5 Department of Biochemistry, Showa Medical University School of Medicine, Tokyo, Japan

**Keywords:** Cancer models, Oncogenes, Epigenomics

## Abstract

Large-scale genome sequencing analyses have identified driver gene mutations (DGMs) in most cancers as well as their associated tumorigenic mechanisms. However, a small fraction of cancers are not positive for these canonical DGMs, leaving the mechanisms underpinning their formation a mystery. We hypothesized that canonical DGM-negative cancers might be driven by activation of the transcriptional coactivator YAP1 that led to the induction of epigenetic changes. To test this theory, we established a mouse mosaic model of hepatocellular carcinoma (HCC) in which we induced YAP1-TEAD activation in a few hepatocytes. Whole-exome sequencing did not identify canonical DGMs in HCCs, but bisulfite sequencing revealed widespread DNA demethylation leading to the transcriptional activation of multiple oncogenes. Knockdown of the DNA demethylation-promoting gene, *Tet1*, attenuated HCC formation in these mice. Single-cell spatial transcriptomics identified a *Tet1*-high subpopulation of HCC cells that interacted with other hepatic cell types. Our mechanistic mouse data align with the observation that YAP1–TEAD–TET1-associated signatures were also elevated in hepatocytes from patients with Fontan-associated liver disease (FALD), a condition associated with the development of HCCs with lower frequencies of canonical DGMs. Our study suggests that the YAP1-TEAD-TET1 axis promotes canonical DGM-negative HCC development, and provides new insights into the molecular processes involved.

## Abstract (web summary)

Analysis of YAP1-induced hepatocellular carcinoma (HCC) using genomic, epigenomic, and spatial transcriptomic methods reveals that the YAP1–TEAD–TET1 axis drives HCC via DNA demethylation rather than canonical driver gene mutations.

## Introduction

Most cancers arise from rogue cells characterized by the activation of proteins encoded by oncogenes or the inactivation of proteins encoded by tumor suppressor genes^1,2^. These genes are commonly referred to as “driver genes”, and the functional changes they cause are typically attributed to DNA mutations. Recent large-scale whole-genome sequencing (WGS) efforts have shed light on the landscape of driver gene mutations (DGMs) linked to tumorigenesis. For instance, a pan-cancer analysis of over 2600 tumors across 38 types identified DGMs in >90% of cases, underscoring the importance of mutations to coding sequences^3^. However, approximately 5% of these tumors were negative for canonical DGMs, even after accounting for potential technical limitations in mutation-calling^3,4^. A subsequent high-depth WGS analysis of 494 hepatocellular carcinoma (HCC) samples revealed the existence of canonical DGM-negative HCCs^5^. In particular, lower frequencies of canonical DGMs were found in HCCs linked to Fontan-associated liver disease (FALD), which arises after the Fontan procedure is used to treat congenital heart disease^6–10^. In FALD patients, the liver is subjected to persistent hemodynamic stress due to chronically elevated central venous pressure, leading to the development of both HCCs and intrahepatic cholangiocarcinoma (ICC)^6–9^. Recent studies have reported some genomic mutations in FALD-associated HCCs^8,10^. However, canonical HCC DGMs, including *CTNNB1*, *TP53*, and *TERT* promoter mutations, were less frequent in FALD-associated HCCs than in other types of HCCs^10^. These findings imply that HCCs can be induced by alternative mechanisms that can drive carcinogenesis independently of genomic canonical DGMs^11^.

Several examples of such canonical DGM-negative mechanisms have been uncovered in model organisms. For example, in *Drosophila*, knockdown of the Polycomb group protein PH, a key epigenetic regulator, leads to “epigenetically initiated cancers^12^”. In this model, PH knockdown triggers DGM-negative tumorigenesis through activation of the JAK-STAT pathway (a Polycomb target). In mice, the artificial expression of multiple reprogramming factors (*Oct3/4*, *Sox2*, *Klf4*, and *Myc*) induces renal cancer dependent on the perturbation of DNA methylation patterns^13^.

Despite the above, most canonical DGM-negative mechanisms have been difficult to study. Among these is tumorigenesis linked to the transcriptional co-activator YAP1, which is controlled by the Hippo signaling pathway and interacts with TEAD transcription factors to form a complex that is widely recognized as a potent oncogenic factor^14–17^. This activation pathway is often triggered by extracellular stimuli such as mechanical force or fibrosis^18,19^. Once activated by Hippo signaling, the YAP1-TEAD complex regulates the expression of myriad target genes, including those involved in cell proliferation^14^. Persistent YAP1 activation in mouse hepatocytes leads to enhanced cell proliferation and dedifferentiation, ultimately inducing liver enlargement and HCC^20,21^. Consistent with these findings, abnormal accumulation of YAP1 in cellular nuclei has been observed in various human cancers and is particularly evident in liver tumors. Indeed, nuclear YAP1 accumulation is present in more than 50% of HCC patients and correlates positively with aggressive malignancy and poor prognosis^22,23^.

Interestingly, large-scale cancer genome sequencing efforts have revealed that oncogenic mutations of the YAP1 gene and genes upstream of YAP1 in the Hippo pathway are very rare in human cancers^14,16,24–27^. This paradox suggests that the frequent YAP1 activation observed in human malignancies results from molecular events other than mutations in the genes constituting the Hippo-YAP1-TEAD pathway. While TAZ (WWTR1) is a closely related paralog of YAP1 and also a Hippo pathway effector, previous studies have indicated that liver tumor phenotypes are mostly dependent on YAP1 and only partially dependent on TAZ^21,28^. These observations prompted us to focus on YAP1 in the present study.

DNA methylation is an epigenetic modification that regulates gene expression^29,30^. Such methylation primarily occurs on cytosine bases within CpG islands located in gene promoter regions. Altered DNA methylation is strongly associated with cancer development and progression, in that many malignancies display hypomethylation of oncogenes and hypermethylation of tumor suppressor genes^31–34^. Changes in DNA methylation are controlled by DNA methyltransferases (DNMTs), which add methyl groups to cytosine, and Ten-eleven translocation (TET) methylcytosine dioxygenases, which promote demethylation^35–37^. Recently, Wu et al. identified *Tet1* as a direct target of YAP1-TEAD signaling in mouse liver and determined the status of YAP1-TET1 target genes at 10 days post-YAP1 activation, a pre-malignant stage prior to the onset of HCC^38^. This group also reported that *Tet1* was essential for YAP1-induced HCC development, but much remains unknown regarding Tet1-expressing hepatocytes, their target genes, and cell–cell interactions in YAP1-induced HCC.

**Fig. 1:**
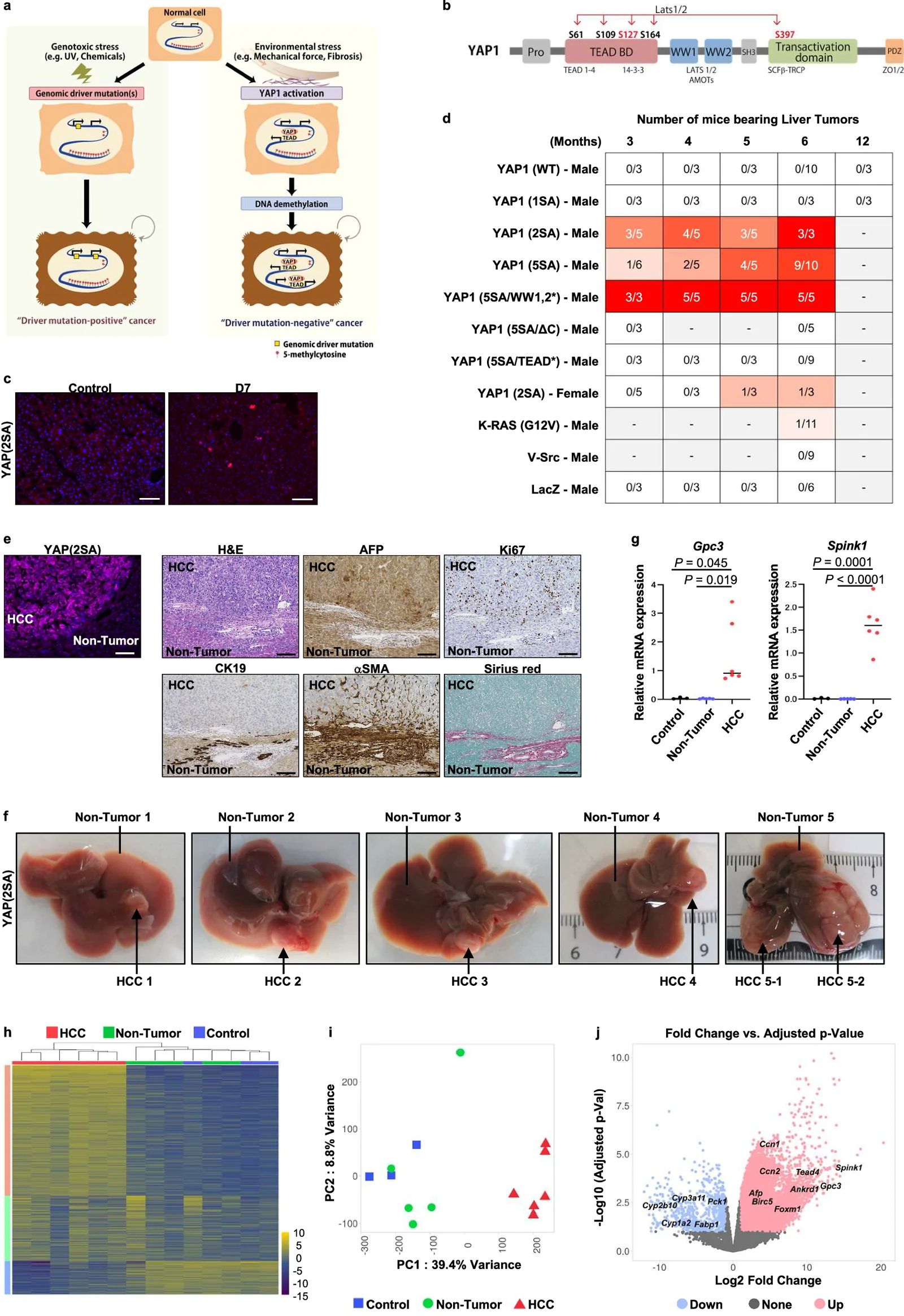
Hypothesis and experimental system for verifying canonical DGM-negative cancer formation. **a** Schematic diagram of our hypothesis regarding mechanisms of driver gene mutation (DGM)-positive and DGM-negative cancer formation. Left panel: In DGM-positive cancer, genotoxic stresses induce mutations in driver genes that lead to tumorigenesis. Right panel: In DGM-negative cancer, non-genotoxic environmental stimuli activate YAP1 in cells. Activated YAP1 then promotes tumorigenesis through DNA demethylation and abnormal activation of demethylation target genes. **b** Diagram of human YAP1 protein domain structure showing Lats1/2 phosphorylation sites (arrows). In the YAP1(2SA) mutant, the serines at positions 127 and 397 (indicated in red) are substituted with alanine. **c** Images of immunostaining using MYCtag antibody to detect exogenous YAP1(2SA) in mouse liver tissue at 7 days post-introduction (D7) or in control liver tissue (*n* > 10). Scale bars, 100 µm. **d** Chart showing the number of mice with a liver tumor relative to the total number of mice in each experimental condition at the indicated number of months after introduction of the indicated plasmids. The sex of the mice receiving the plasmids is indicated. **e** Representative images of (left) exogenous YAP1(2SA) immunostaining using MYCtag antibody, and (right) H&E, AFP, CK19, Ki67, αSMA, or Sirius Red staining, of cancerous (HCC) and non-tumor areas of livers bearing YAP1-TEAD-induced HCCs (*n* > 10). Scale bars, 100 µm. **f** Macroscopic images of four pairs of HCC and non-tumor tissues in livers from four male mice (HCC 1 vs Non-Tumor 1, HCC 2 vs Non-Tumor 2, HCC 3 vs Non-Tumor 3, HCC 4 vs Non-Tumor 4). Two HCCs and one non-tumor region from the same mouse liver (HCC 5-1 vs Non-Tumor 5, HCC 5-2 vs Non-Tumor 5) are shown at the far right. **g** Relative mRNA levels of the indicated HCC marker genes in the tissues in (**f**) as determined by qPCR (*n* = 6). Statistical significance was assessed using one-way ANOVA followed by Tukey’s multiple comparisons test. **h** Biclustering heatmap of RNA-seq analysis of the HCC and non-tumor samples in (**f**) plus control mouse livers (*n* = 3), using the top 3000 differentially expressed genes (DEGs). **i** Principal Component Analysis (PCA) of the data in (**h**). **j** Volcano plot of the differential gene expression analysis using the RNA-seq data shown in (h), comprising YAP1–TEAD-induced HCCs (*n* = 6 biologically independent samples), non-tumor liver tissues (*n* = 5 biologically independent samples).

Based on the above reports, we devised a hypothesis of cancer formation in which malignancy can be mediated by DNA demethylation and not necessarily by canonical DGMs (Fig. 1a). To test our hypothesis, we established a mosaic mouse model in which the development of HCC due to induction by active YAP1 could be followed. Our model was designed to facilitate three key analyses: (1) genomic comparisons of non-tumorous and tumorous regions within the same liver; (2) determination of whether the resulting HCC constituted a homogeneous or heterogeneous cell population; and (3) investigation of cellular interactions among cancer cells as well as between cancer cells and normal non-parenchymal cells. Using this model, we determined that active YAP1 can induce epigenetic changes that lead to canonical DGM-negative HCC.

## Results

### Establishment of a mosaic YAP1-TEAD-induced HCC model

To recapitulate the clonal proliferation of YAP1-activated cells among normal hepatocytes in vivo, we designed a mosaic YAP1-TEAD-induced HCC model to meet three criteria: (1) transfection of active YAP1 alone should cause cancer formation; (2) YAP1-activated cells should undergo clonal proliferation to form a cancer; and (3) mosaic analysis should be able to define interactions between YAP1-activated cells and normal cells. To create active YAP1, we took advantage of the YAP1(2SA) mutant (Fig. 1b) in which phosphorylation-dependent cytoplasmic retention (S127) and proteasomal degradation (S381) are impaired, resulting in constitutive nuclear localization and transcriptional activation^39,40^. Pilot experiments in which C57BL/6J mice were subjected to hydrodynamic tail vein injection (HTVi) of the YAP1(2SA) plasmid under the control of the albumin promoter confirmed the successful establishment of a mosaic model (Fig. 1c).

Next, to identify the functional domains of YAP1 required for tumorigenesis in our model, we transfected a series of plasmids bearing various YAP1 mutants into male and female mice (Fig. 1d). In male mice, we found that hepatocyte expression of YAP1(2SA) or YAP1(5SA) resulted in liver cancer formation at around 4 months (M) post-transfection. In contrast, expression of either wild-type (WT) YAP1 or YAP1(1SA) (single phosphorylation-site mutant at serine 127) did not induce HCC even after 12 months of observation. Furthermore, tumorigenesis was completely abolished in male mice expressing YAP1(5SA) mutants lacking either the C-terminal transactivation domain (5SA/ΔC) or the TEAD-binding domain (5SA/TEAD*). The YAP1(5SA/WW1,2) mutant, which is defective only in WW domain–mediated protein-protein interactions (including binding to transcription factors such as p73), retained the ability to induce HCC in male mice. These results demonstrate that YAP1-induced hepatocarcinogenesis is strictly dependent on its interaction with TEAD transcription factors and the subsequent transactivation of target genes. Notably, the oncogenic potency of YAP1(2SA) was superior to that of both the K-RAS (G12V) and v-Src controls in this model. As expected, we observed a sex difference in that female mice transfected with the YAP1(2SA) plasmid developed HCC less frequently than males. This sex difference is in line with the well-known male predominance of HCC in both mice and humans^41,42^.

When we histologically analyzed liver cancers that were induced by overexpression of the YAP1(2SA) construct and introduced by HTVi, we observed that YAP1 was expressed throughout the malignancy (Fig. 1e, left). Tissue staining with H&E or Sirius Red, or with antibodies to detect AFP (HCC marker), Ki67 (proliferation marker), CK19 (cholangiocyte marker), or αSMA (activated hepatic stellate cells), showed that these liver cancers were HCCs (Fig. 1e, right)^43^. Thus, our experimental system of YAP1-TEAD-induced HCC formation using male mice and the HTVi method met our criteria and was deemed suitable to test our hypothesis.

To analyze YAP1-TEAD-induced HCCs systematically, we examined six pairs of HCCs and their adjacent non-tumor tissues (Fig. 1f, and Supplementary Fig. 1). Four pairs of HCC and adjacent non-tumor tissues were prepared from four male mice (HCC 1–4 vs. Non-Tumor 1–4). In addition, two HCCs were prepared from one male mouse (HCC 5-1 and HCC 5-2) and paired with Non-Tumor 5 tissue. Livers from three normal male mice were used as controls. Quantitative PCR (qPCR) analysis confirmed the significant upregulation of the HCC markers *Gpc3* and *Spink1* in all six HCCs (Fig. 1g).

To define the gene expression profiles of these tissues, we performed RNA-sequencing (RNA-seq) on each pair as well as on control livers (Supplementary Data 1). Hierarchical clustering and principal component analysis (PCA) revealed that all YAP1-TEAD-induced HCCs displayed remarkably similar gene expression patterns and clustered separately from the non-tumor tissues (Fig. 1h, i and Supplementary Fig. 2a). Volcano plot analysis identified a shared set of differentially expressed genes (Fig. 1j). We then quantified and confirmed these altered expression levels by qPCR. Several canonical YAP1–TEAD target genes, including *Tead4, Ccn1*, and *Ccn2*, were significantly upregulated in HCCs (Supplementary Fig. 2b). In addition, enrichment of the YAP/TAZ transcriptional signature was confirmed by gene set enrichment analysis (GSEA) (Supplementary Fig. 2c)^44^. Conversely, the most significantly downregulated genes included metabolic enzymes such as *Cyp3a11* and *Pck1* (Supplementary Fig. 2d). Taken together, these data strongly suggest that YAP1-TEAD-induced HCCs are driven by a shared gene expression mechanism.

### YAP1-TEAD activation induces the formation of canonical DGM-negative HCCs

**Fig. 2:**
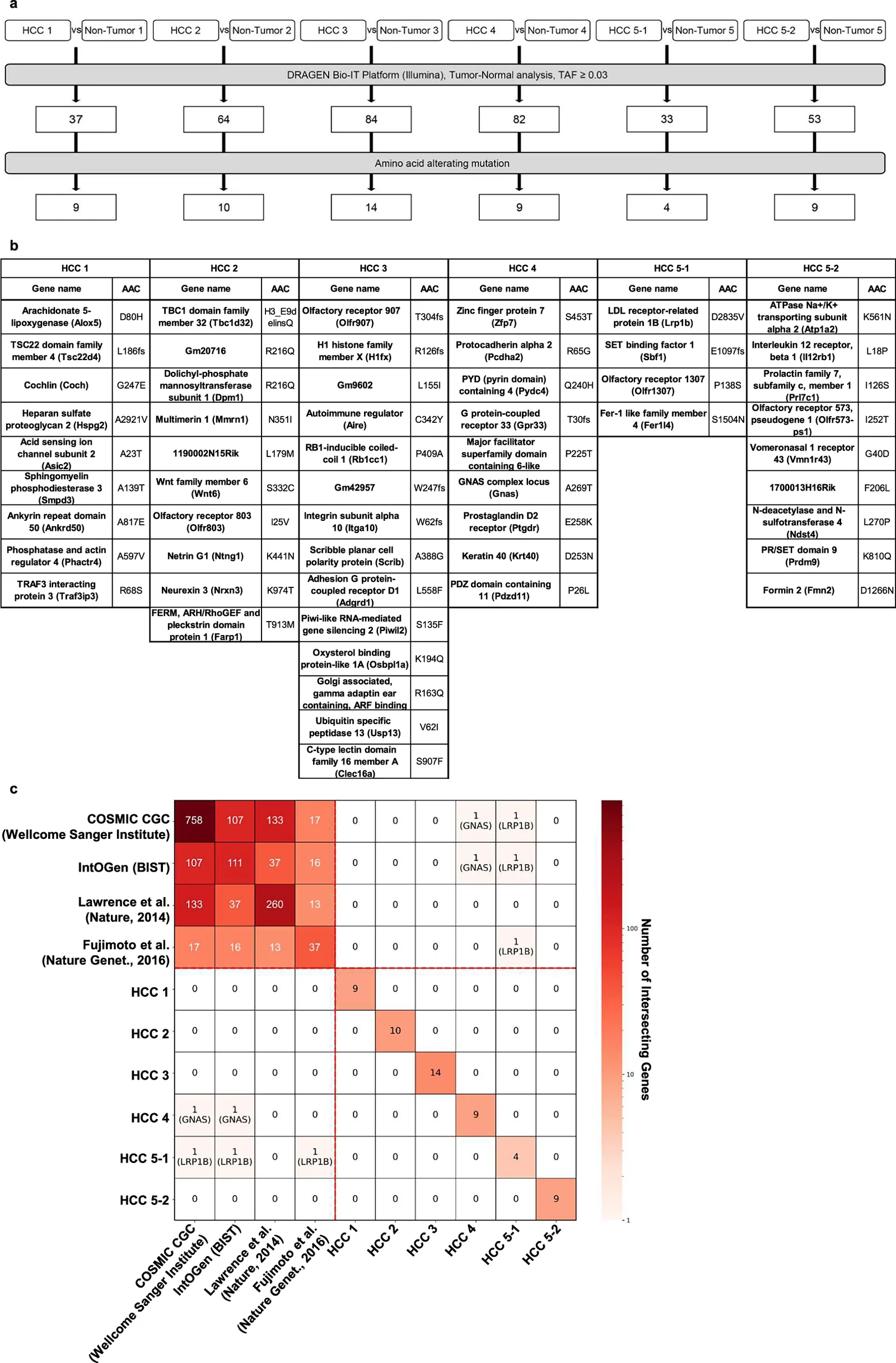
Whole exome sequencing (WES) analysis of YAP1-TEAD-induced mouse HCCs. **a** Flow chart of WES analysis for the six HCC vs Non-Tumor pairs in Fig. 1f. Numbers in the middle row indicate the number of mutations present after quality metrics and TAF filtering by the DRAGEN Bio-IT Platform. Numbers in the lower row indicate the number of amino acid-altering mutations in the middle row. **b** Gene names and amino acid changes (AAC) of amino acid-altering somatic mutations identified in the six HCCs in Fig. 1f. **c** Comparison of mutated gene lists identified in the six HCCs in (**b**) with reported driver gene lists from COSMIC CGC, IntOgen, Lawrence et al., and Fujimoto et al.^45–48^, as indicated.

To determine whether our model could give rise to canonical DGM-negative HCCs, we performed whole-exome sequencing (WES) on our six pairs of HCC and adjacent non-tumor tissues (Fig. 2a). Genomic DNA was extracted from each tissue and libraries were prepared using the Twist Mouse Exome Panel (Twist Bioscience). We then conducted deep WES to an average depth exceeding 330 times. Reads were subsequently mapped to the mouse reference genome using the DRAGEN Bio-IT Platform (Illumina). Following the removal of duplicate reads, somatic single-nucleotide variants (SNVs) and short insertions/deletions (indels) were called via a tumor vs. normal comparative analysis. Candidate mutations were filtered based on quality metrics, including LOD scores and read depth, and we retained those candidate mutations with a tumor allele frequency (TAF) of more than 0.03 (Supplementary Data 2–7). This filtering process yielded 37, 64, 84, 82, 33, and 53 somatic mutations per HCC, respectively. Among these, we further isolated a total of 55 non-synonymous (amino acid-altering) mutations across the six HCCs: 9, 10, 14, 9, 4, and 9 mutations per cancer, respectively. These mutations, including the affected genes and corresponding amino acid changes, are summarized in Fig. 2b.

Intriguingly, the mutational profiles of the six HCCs were entirely distinct, with no shared mutations observed among them (Fig. 2b). To determine whether any of these mutations was a DGM, we compared these mutated genes to those listed in several established human driver gene databases, including the COSMIC Cancer Gene Census (CGC, v102)^45^, IntOgen (BIST)^46^, and driver lists reported by Lawrence et al.^47^ and Fujimoto et al.^48^. Manual PubMed searches were also conducted. None of the coding mutations identified in HCC1, HCC2, or HCC5-2 corresponded to known canonical DGMs in the databases analyzed (Fig. 2c, and Supplementary Data 8). Based on these database comparisons and literature searches, we concluded that these three malignancies were canonical DGM-negative HCCs. On the other hand, HCC3 contained mutations in the tumor suppressor genes *Scribble planar cell polarity protein* (*Scrib*) and *RB1 inducible coiled-coil 1* (*Rb1cc1*); HCC4 had a mutation in the oncogene *GNAS complex locus* (*Gnas*); and HCC 5-1 had a mutation in the tumor suppressor *LDL receptor-related protein 1B* (*Lrp1b*). Thus, YAP1-TEAD activation led to HCC formation in the absence of canonical DGMs in about 50% of cases.

### *Tet1* knockdown attenuates YAP1–TEAD-induced HCC formation

**Fig. 3:**
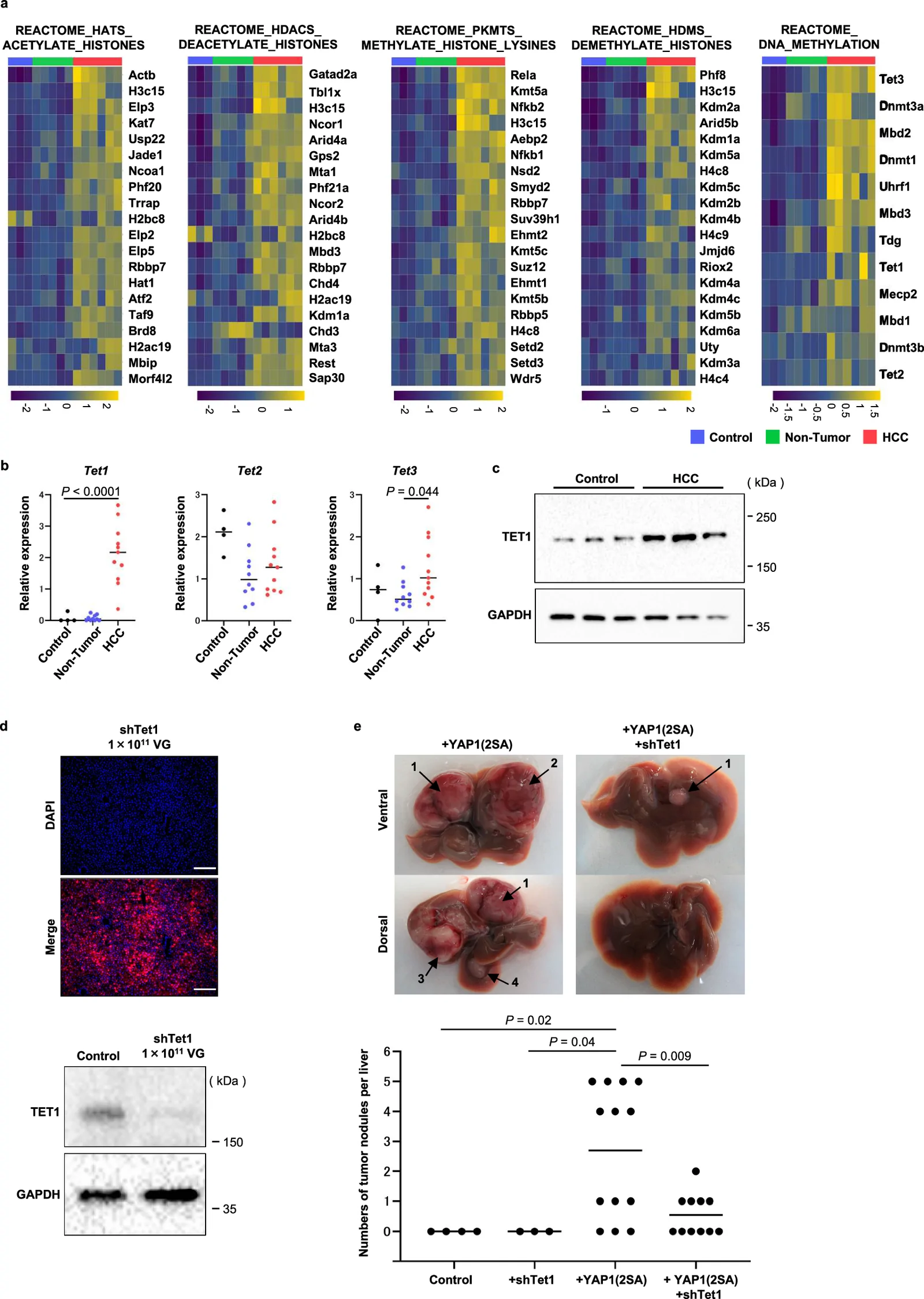
Analyses of epigenetic changes and TET1 knockdown effects in YAP1-TEAD-induced HCCs. **a** Heatmap visualization of expression of the indicated epigenetics-related gene sets using the RNA-seq results from Fig. 1. **b** Relative mRNA levels as determined by qPCR of the indicated *Tet* gene family members in control liver tissue, Non-Tumor areas of HCCs, and HCCs. The initial cohort of 6 HCCs plus 5 additional YAP1-TEAD-induced HCCs were examined (*n* = 11). Statistical significance was assessed using one-way ANOVA followed by Tukey’s multiple comparisons test. **c** Representative Western blotting to detect TET1 protein levels in a control liver and a YAP1-TEAD-induced HCC (*n* = 3). GAPDH, loading control. **d** Top: Representative RFP fluorescence imaging to detect shTet1-expressing hepatocytes in mouse liver at 2 weeks post-transduction. Scale bars, 100 µm. Bottom: Representative Western blotting to detect TET1 protein levels in a control liver and a shTet1-induced liver. **e** Top: Representative macroscopic images of YAP1(2SA)-induced HCCs with (left) or without (right) concurrent *Tet1* knockdown. Numbers with arrows indicate individual tumor nodules. Bottom: Quantitation of number of mice developing the indicated numbers of tumor nodules per liver. Each dot represents an individual mouse. Statistical significance was assessed using one-way ANOVA followed by Tukey’s multiple comparisons test.

To investigate whether epigenetic reprogramming could be the common oncogenic mechanism underlying both canonical DGM-negative and DGM-positive YAP1-TEAD-induced HCCs, we analyzed the expression of key epigenetic regulators in our six HCCs. First, we extracted expression data for genes linked to histone acetylation, histone deacetylation, histone methylation, histone demethylation, and DNA methylation from the RNA-seq analysis shown in Fig. 1. These results indicated that the expression levels of almost all epigenetic reprogramming genes were enhanced in our six YAP1-TEAD-induced HCCs (Fig. 3a). We next focused on the YAP1-TEAD target *Tet1*, a gene whose product promotes DNA demethylation. Among members of the *Tet* gene family, only *Tet1* was significantly upregulated at the mRNA level compared to non-tumor controls in a total of 11 HCCs (HCC1, 2, 3, 4, 5-1, 5-2 and an additional 5 YAP1-TEAD HCCs that were separately induced) (Fig. 3b). Consistent with this rise in mRNA levels, TET1 protein levels were also elevated in YAP1-TEAD-induced HCCs (Fig. 3c, and Supplementary Fig. 3a).

To determine if TET1 function was required for YAP1-TEAD-induced HCC formation, we performed in vivo knockdown experiments using shRNA targeting *Tet1* (shTet1) in mouse livers. Efficient transduction of nearly all hepatocytes with an AAV8-shTet1 vector resulted in a significant reduction in TET1 protein levels (Fig. 3d, and Supplementary Fig. 3b). Evaluation of tumor burden at 4 months post-HCC induction revealed that YAP1(2SA) delivery alone induced macroscopic tumors in 10 of 13 mice, yielding an average of 2.7 tumor nodules per liver (Fig. 3e). In contrast, knockdown of TET1 combined with YAP1(2SA) markedly suppressed the latter’s oncogenic potential since only 5 of 11 mice developed macroscopic tumors, and the average tumor nodule count was reduced to 0.5 per liver (*P* = 0.009). These results indicate that *Tet1* knockdown attenuates YAP1–TEAD-induced HCC formation, reducing tumor penetrance and multiplicity.

### YAP1-TEAD activation induces genome-wide DNA methylation changes that result in multiple oncogene expression

**Fig. 4:**
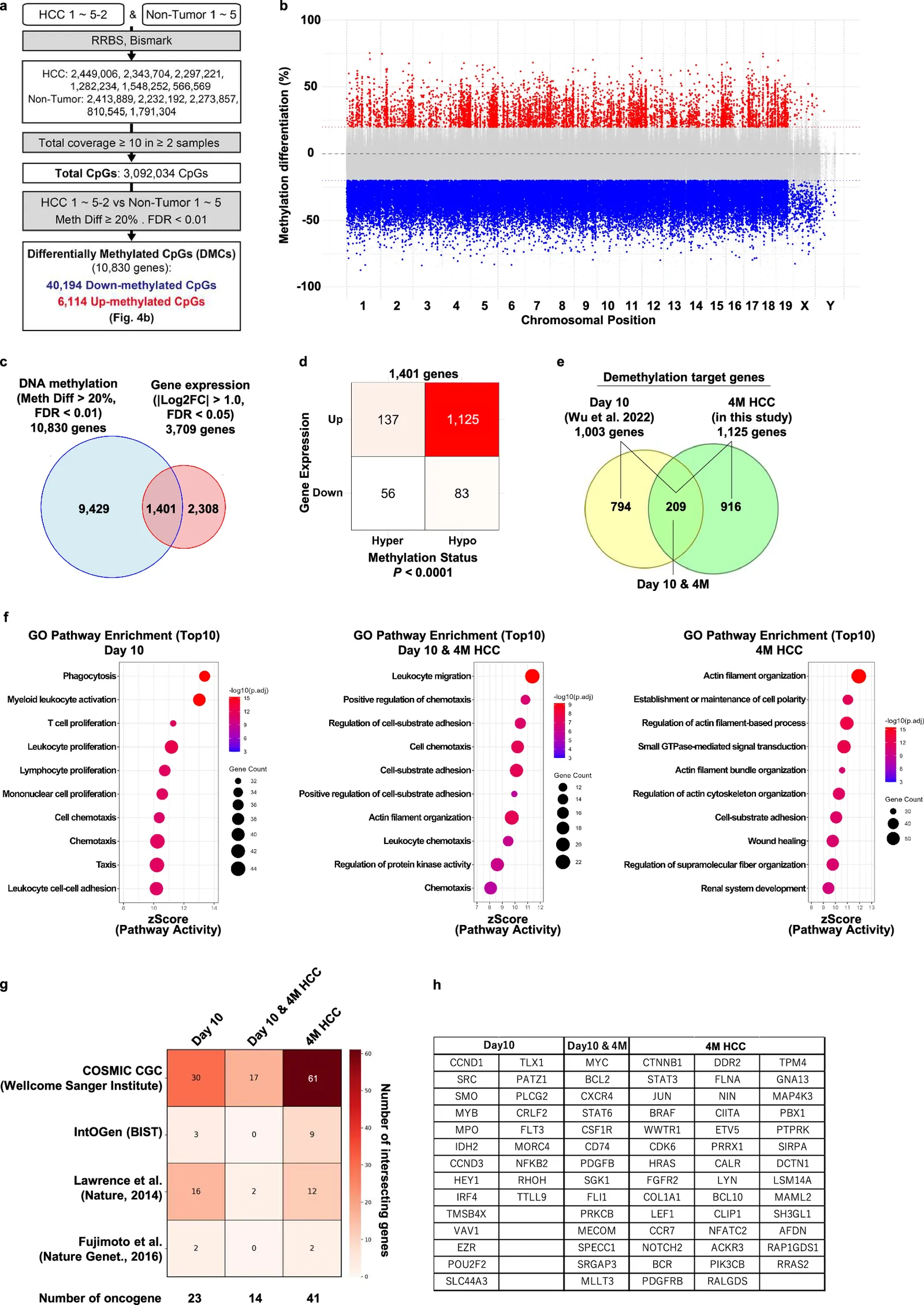
DNA methylation analysis and identification of target genes in YAP1-TEAD-induced HCCs. **a** Flow chart of RRBS analysis of the 6 HCC vs Non-Tumor pairs in Fig. 1f. Third row: Numbers of methylated CpG sites quantified in each tissue. Fifth row: Number of CpG sites after total coverage filtering. Seventh row: Numbers of CpG sites showing significantly decreased (blue) or increased (red) DNA methylation in YAP1-TEAD-induced HCCs, and their corresponding gene counts. **b** Genome-wide methylation alteration plot. Each dot represents a CpG site, with its chromosomal position indicated on the x-axis and its rate of methylation level change indicated on the y-axis. Genes with significantly increased methylation in HCCs are plotted in red, decreased methylation in blue, and unchanged in gray. **c** Venn diagram examining the overlap between 10,830 genes with significantly altered DNA methylation (Meth Diff > 20%, FDR < 0.01) and 3709 genes with significantly altered gene expression (|Log2FC| > 1.0, FDR < 0.05). **d** Contingency table classifying the 1401 relevant genes identified in (**c**) based on DNA hyper- or hypo-methylation vs. increased (up) or decreased (down) gene expression. Statistical significance was assessed using a two-sided Fisher’s exact test. **e** Venn diagram examining the overlap between the demethylation target genes activated in the liver at 10 days after YAP1 activation (data of Wu et al.) vs. those in the YAP1-TEAD-induced HCCs from (**d**) above. **f** Dot plots showing Gene Ontology (GO) enrichment analysis results for the demethylation target genes that were identified in (**e**) and expressed on Day 10, Day 10 & 4 M HCC, or 4 M HCC. **g** Comparison of the demethylation target genes identified in (e) with reported driver gene lists from COSMIC CGC, IntOgen, Lawrence et al., and Fujimoto et al.^45–48^. classified by time of expression, as indicated. The number of oncogenes identified is shown below. **h** Chart showing list of the oncogenes identified in (**g**) that were expressed on Day 10, Day 10 & 4 M HCC, or 4 M HCC.

To determine whether YAP1-TEAD activation alters DNA methylation patterns, we performed reduced representation bisulfite sequencing (RRBS) on the same six pairs of HCC and non-tumor tissues, following the workflow outlined in Fig. 4a. Genome-wide methylation analysis identified 40,194 significantly hypomethylated CpG sites and 6,114 significantly hypermethylated CpG sites in HCCs (Fig. 4a, b). These differentially methylated cytosines (DMCs) were annotated for 10,830 genes (Fig. 4a, c). Integration of these DMCs with our RNA-seq data in Fig. 1 and Supplementary Data 1 identified 1401 genes exhibiting significant changes in both DNA methylation and gene expression (Fig. 4c). Among these, 1125 genes were hypomethylated and upregulated (*P* < 0.0001; Fig. 4d, and Supplementary Data 9), and so were identified as DNA demethylation targets in YAP1-TEAD-induced HCCs.

Next, to delineate the time course of these demethylation changes during tumor progression, we compared our 1125 demethylation target genes identified at 4 months following YAP1 activation (4M; HCC stage) with Wu et al.’s 1003 targets identified at Day 10 following YAP1 activation (a pre-malignant stage)^38^. This comparison resolved the targets into three distinct groups: 794 genes exhibiting demethylation changes only at Day 10; 209 genes exhibiting changes at both Day 10 and 4M HCC; and 916 novel target genes exhibiting changes only at the 4M HCC stage (Fig. 4e, and Supplementary Data 10). Gene Ontology (GO) analysis revealed that both the Day 10-specific and shared Day 10 & 4M HCC genes were primarily enriched in immune response-related events, such as leukocyte activation (Fig. 4f, and Supplementary Data 11 and 12). In contrast, the 4M HCC-specific genes were enriched in malignant features such as actin filament organization, establishment or maintenance of cell polarity, and cell-substrate adhesion (Supplementary Data 13). These results suggest that, as HCC progresses, the biological roles of the proteins encoded by YAP1-TET1-induced DNA demethylation target genes transition from early immune microenvironment modulation to the direct acquisition of malignant cellular phenotypes.

To determine if any of the demethylated target genes possessed oncogenic potential, we compared our three groups with established lists of driver genes, including those of COSMIC^45^, IntOgen^46^, Lawrence et al.^47^, and Fujimoto et al.^48^. (Fig. 4g). We identified multiple known oncogenes across all groups (Fig. 4h, and Supplementary Fig. 4). Specifically, the Day 10 group included *Ccnd1* and *Src*; the shared group (Day 10 & 4M HCC) included *Myc*, *Bcl2*, and *Pdgfb*; and the 4M HCC group included *Ctnnb1*, *Stat3*, *Braf*, and *Hras* (Supplementary Data 14, and Supplementary Fig. 5). Taken together, these data indicate that demethylation-mediated upregulation of multiple oncogenes may be involved in the initiation and progression of YAP1-TEAD-induced HCCs.

### YAP1-TEAD-induced HCCs are comprised of multiple distinct cell subpopulations

**Fig. 5:**
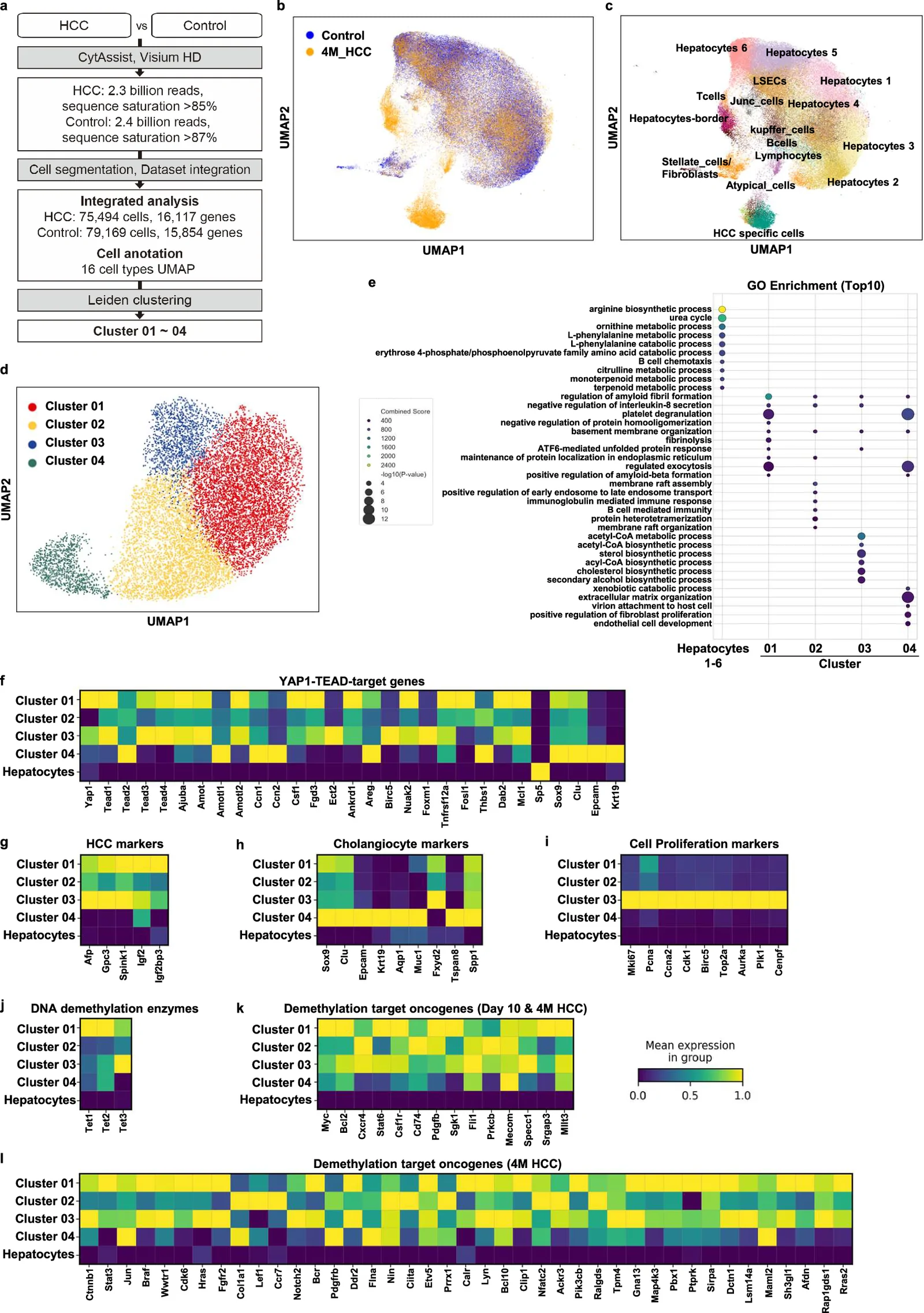
Single-cell spatial transcriptomics analysis of YAP1-TEAD-induced HCCs. **a** Flowchart of Visium HD analysis using normal liver (Control) and liver tissue containing YAP1-TEAD-induced HCC (HCC). Third row: Total reads and sequence saturation. Fifth row: Numbers of cells and genes identified in HCC vs. Control, and number of cell types after segmentation and integrated analysis. **b** UMAP of all cells identified in control liver (blue) and in HCC liver at 4 months post-transfection (orange). **c** Cell type annotation of the data in (**b**). **d** Leiden clustering analysis of HCC-specific cells. **e** GO pathway analysis for genes specific to HCC Clusters 01-04 and normal hepatocyte Clusters 1-6 (Hepatocytes). Matrix analysis results for: **f** the indicated YAP1-TEAD-target genes; **g** HCC markers; **h** cholangiocyte markers; **i** cell proliferation markers; **j** DNA demethylation enzymes; **k** Day 10 & 4M HCC DNA demethylation target oncogenes; and **l** 4M HCC DNA demethylation target oncogenes as determined for HCC Clusters 01-04 and normal hepatocyte Clusters 1-6 (Hepatocytes).

Natural HCCs are cancers displaying substantial intratumoral heterogeneity, but the molecular mechanism(s) driving this heterogeneity are not clear. To investigate whether the HCCs in our model were homogeneous or heterogeneous in their cell populations, we used Visium HD to perform comparative single-cell spatial transcriptomics on normal liver tissues (hereafter, “control liver”) and YAP1-TEAD-induced 4M HCC tissues (“HCC liver”) (Fig. 5a, and Supplementary Data 15). After quality control and batch effect correction, we analyzed 79,169 cells from control liver and 75,494 cells from HCC liver. UMAP visualization of the integrated dataset revealed that, while the transcriptomic profile of the majority of cells overlapped between control and HCC livers, distinct HCC-specific populations were clearly evident (Fig. 5b). Annotation based on established marker gene expression patterns combined with spatial localization classified the liver cells examined into 16 distinct cell types (Fig. 5c). Of these, the populations designated as “hepatocytes-border”, “atypical cells” and “HCC-specific cells” were found only in HCC liver and not in control liver. Sub-clustering of the population of “HCC-specific cells” using the Leiden algorithm unexpectedly resolved this population into four clusters (Cluster 01, Cluster 02, Cluster 03, and Cluster 04) (Fig. 5d). This finding indicated that our mouse HCCs exhibited intratumoral heterogeneity, just as occurs in human HCCs. To characterize these four clusters, we performed GO pathway analysis using the top 100 genes specifically expressed in each cluster (Fig. 5e). The results showed that each of the four HCC clusters displayed a unique gene expression signature that was distinct from that of any other HCC cluster and also from that of any normal hepatocyte cluster (Hepatocytes 1-6). These data indicate that malignant cells can generate considerable intratumoral heterogeneity during cancer development.

We next performed a matrix analysis to score each HCC cluster for the expression of key gene sets and discovered that these HCC cell subsets fell into several distinct functional states. All four clusters expressed *Tead* family genes and YAP–TEAD target genes, whereas elevated endogenous mouse *Yap1* expression was observed in Clusters 01, 03, and 04, but not in Cluster 02 (Fig. 5f). Among these clusters, Clusters 01, 02, and 03 expressed HCC markers (Fig. 5g), whereas Cluster 04 expressed cholangiocyte markers (Fig. 5h). This finding is consistent with a previous report showing that YAP1 activation can induce the dedifferentiation of hepatocytes and their subsequent differentiation into a cholangiocyte-like state^20,21^. Cluster 03 predominantly expressed cell proliferation markers (Fig. 5i), indicating that it contained the primary proliferating cell population. Of note, Cluster 01 was marked by higher *Tet1* expression than Clusters 02, 03, and 04 (Fig. 5j, and Supplementary Fig. 6a). Cluster 01 also expressed DNA demethylation target genes that function as oncogenes, both targets shared between Day 10 and 4M HCC (*Myc*, *Bcl2*, *Stat6*, *Csf1r*, *Pdgfb*, *Sgk1*, *Srgap3*, and *Mllt3*; Figs. 5k), and 4M HCC-specific targets (*Stat3*, *Braf*, *Wwtr1*, *Cdk6*, *Hras*, *Fgfr2*, *Bcr*, *Ddr2*, *Etv5*, *Calr*, *Lyn*, *Clip1*, *Pik3cb*, *Gna13*, *Map4k3*, *Pbx1*, *Ptprk*, *Sirpa*, *Dctn1*, *Sh3gl1*, *Afdn*, and *Rras2*; Fig. 5l, and Supplementary Data 16). Thus, the activation of the YAP1-TEAD-TET1 axis and the subsequent expression of multiple oncogenes occurred predominantly in Cluster 01.

**Fig. 6:**
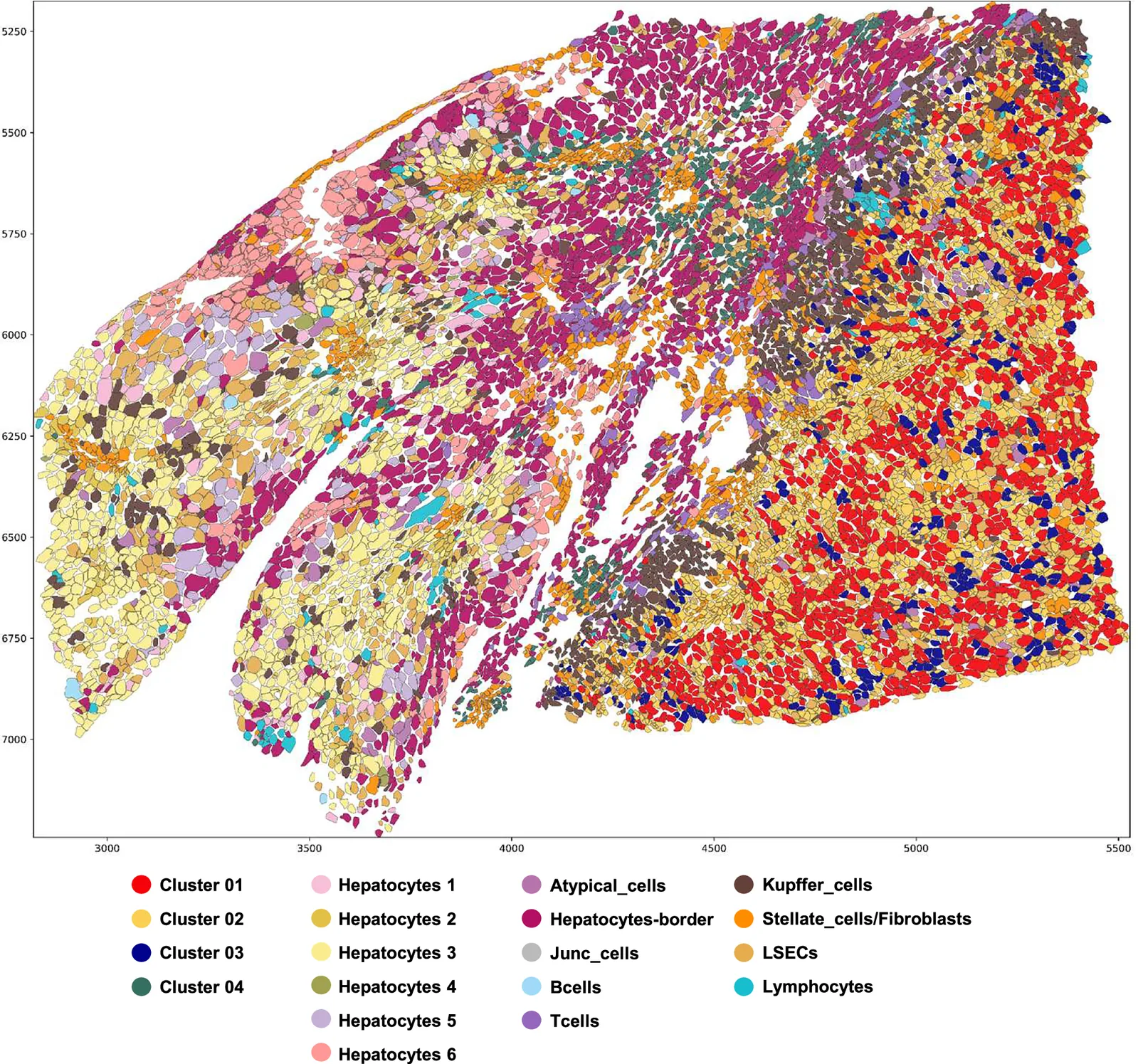
Spatial distribution of cell populations in YAP1–TEAD-induced HCCs. Spatial distribution of the indicated cell populations, shown by color, in YAP1–TEAD-induced HCC and surrounding tissue, based on the single-cell spatial transcriptomic data presented in Fig. 5b–l. The boundary line indicating the histologically defined HCC and non-HCC regions is shown in Supplementary Fig. 6b.

Our mosaic model enables the investigation of cellular interactions among cancer cells as well as between these cancer cells and normal non-parenchymal cells. We leveraged this system to perform advanced spatial analyses and visualize the architectural organization of the Cluster 01-04 subpopulations. To gain insights into how HCC tumors form, we mapped 19 major cell types back to their spatial locations within the original tissue section (Fig. 6, and Supplementary Fig. 6b). This analysis revealed that the HCC liver could be broadly divided into three architectural areas: a “normal region” predominantly composed of normal hepatocytes (Hepatocytes 1–6); a “border region” comprising “Hepatocytes-border” cells, Kupffer cells, lymphocytes, and Cluster 04 HCC cells; and an “HCC region” predominantly occupied by Cluster 01–03 HCC cells, alongside infiltrating Kupffer cells, stellate cells/fibroblasts, and liver sinusoidal endothelial cells (LSECs). Notably, these subpopulations were not randomly intermingled but tended to form spatial aggregates. Collectively, these observations suggest that a YAP1-activated hepatocyte(s) proliferates and differentiates into at least four molecularly and spatially distinct cell subpopulations to form an HCC.

### HCC subpopulations establish intercellular communication networks distinct from those in normal liver tissues

**Fig. 7:**
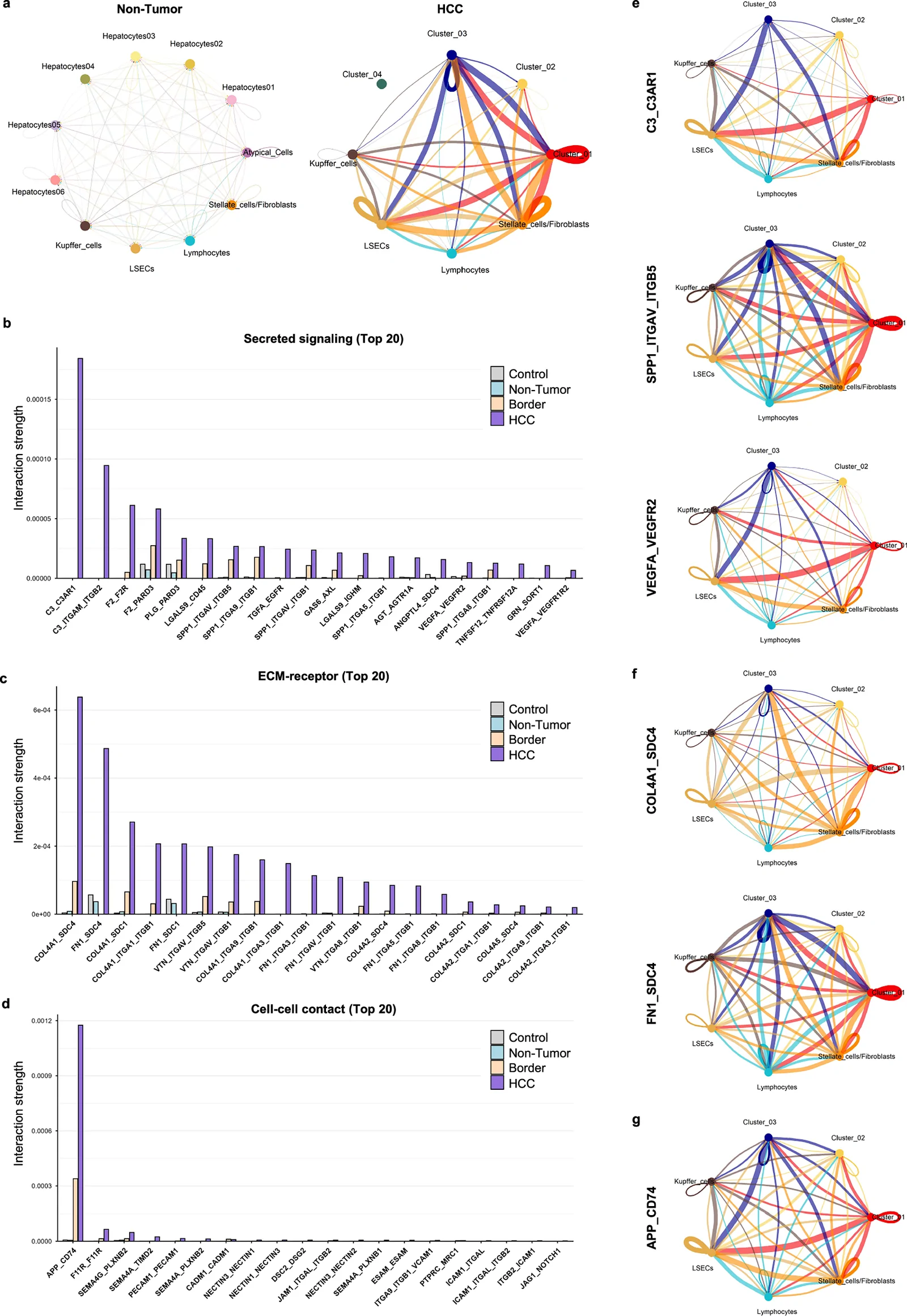
Ligand-receptor analysis of YAP1-TEAD-induced HCC. **a** Aggregated circle plots illustrating the total interaction strength of inferred communication networks between the indicated cell types and HCC clusters in Non-Tumor (left) and HCC (right) regions. Edge width represents the sum of communication probabilities, scaled based on the global maximum value across all regions. The top 20 signaling pathways enriched in the HCC region, categorized as either **b** “Secreted signaling”, **c** “ECM-receptor”, or **d** “Cell-cell contact”. The y-axis indicates the interaction strength (maximum communication probability) for each ligand-receptor pair. **e**–**g** Circle plots of representative ligand-receptor pairs that were significantly activated between the indicated cell types and HCC clusters in the HCC region. Shown are interactions for: **e** “Secreted signaling” (C3-C3AR1, SPP1-ITGAV-ITGB5, and VEGFA-VEGFR2), **f** “ECM-receptor” (COL4A1-SDC4 and FN1-SDC4), and **g “**Cell-cell contact” (APP-CD74). The complete ranked lists are provided in Supplementary Data 17–19.

To investigate whether the four HCC cell subpopulations identified above interacted during tumorigenesis, we examined intercellular communication networks within the tumor microenvironment (TME) by performing a CellChat analysis of non-tumor vs. border vs. HCC regions of liver tissue and compared these findings to a normal control liver^49^. Intercellular interactions were significantly increased in HCC regions compared to non-tumor areas (Fig. 7a). In particular, Cluster 01 emerged as a key signaling hub communicating with Cluster 03, LSECs, and stellate cells/fibroblasts (Fig. 7a). To elucidate which ligand-receptor (L-R) interactions were driving this network, we identified the most prominent L-R pairs within the HCC region and compared their interaction strengths in the non-tumor vs. border vs. HCC regions of HCC livers as compared to normal control livers (Fig. 7b–d). Three signaling categories were examined. In the “Secreted signaling” category, the top-ranked L-R pairs included soluble mediators associated with immune modulation and angiogenesis (Fig. 7b)^50–52^. For instance, Complement C3 signaling was highly prominent, with C3 from HCC Clusters 01 and 03 targeting LSECs (Fig. 7e, and Supplementary Data 17). Furthermore, these clusters engaged LSECs via the VEGFA–VEGFR2 and SPP1–Integrin axes, which are pathways linked to angiogenesis and poor patient prognosis^53,54^. In the “ECM-receptor” category, the identified L-R pairs were mainly structural proteins that build the tumor scaffold and orchestrate microenvironmental stiffness (Fig. 7c)^55^. For example, COL4A1 derived from fibroblasts and LSECs primarily targeted SDC4 on HCC clusters (Fig. 7f, and Supplementary Data 18). In the “Cell-cell contact” category, the APP–CD74 axis emerged as the sole prominent interaction (Fig. 7d). Specifically, APP on Cluster 01 targeted CD74 on LSECs with high probability (Fig. 7g, and Supplementary Data 19). Importantly, several key mediators of these interactions, including Spp1 and App, were previously identified as YAP1–TEAD–TET1-mediated DNA demethylation targets (Fig. 4d, and Supplementary Data 9). These results suggest that HCC cells, particularly Clusters 01 and 03, utilize a diverse repertoire of L-R interactions to engage with normal non-parenchymal cells and shape the HCC TME (Supplementary Fig. 7).

### *TET1* gene expression is elevated in FALD patient hepatocytes

**Fig. 8:**
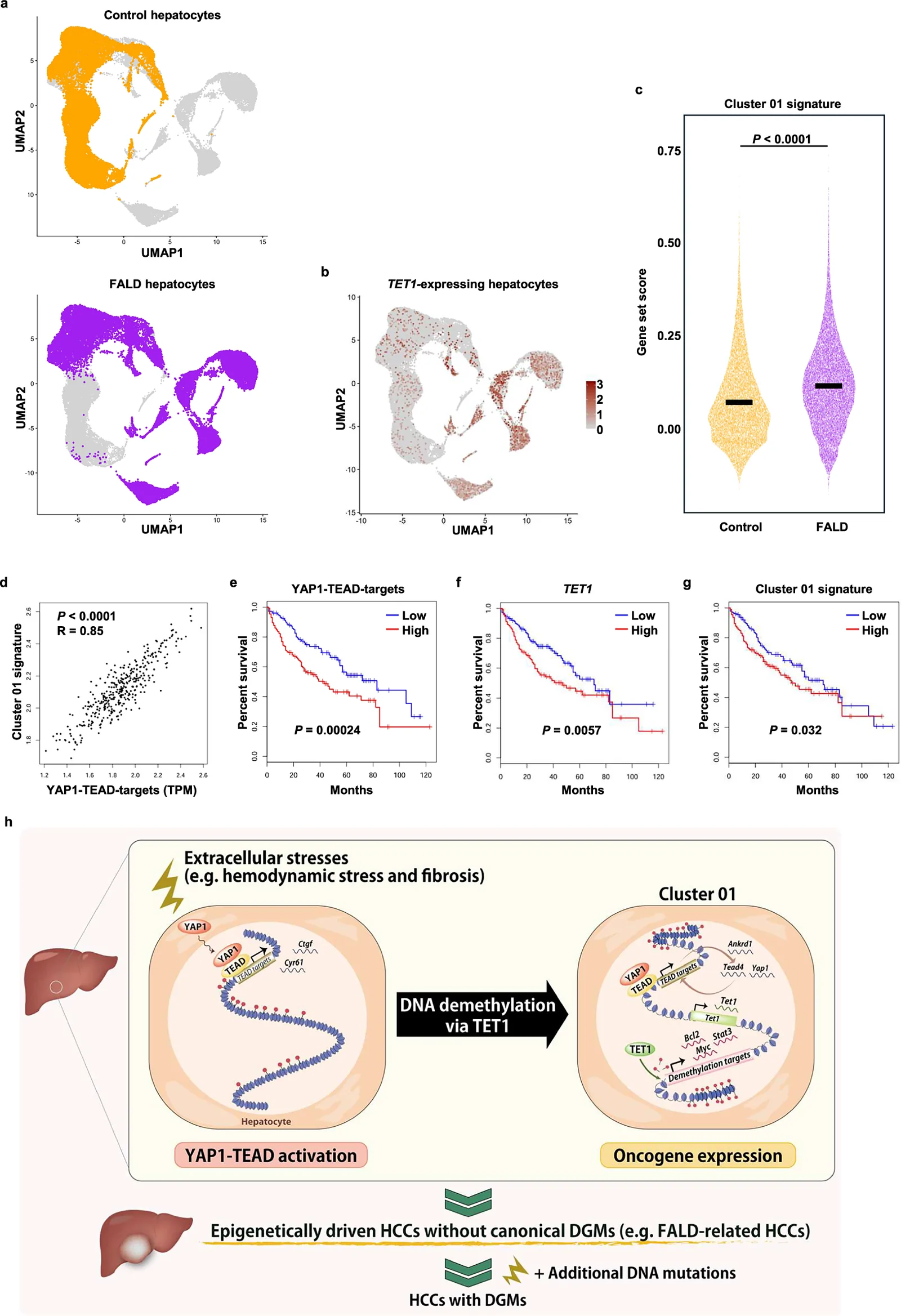
Detection of the YAP1-TEAD-TET1-associated demethylation gene signature in FALD hepatocytes and survival analysis of HCC patients. **a**–**c** Analyses were performed using hepatocytes from control livers (*n* = 2 biologically independent patient samples; GSM6997741 and GSM6997744) and FALD livers (*n* = 4 biologically independent patient samples; GSM6997746, GSM6997748, GSM6997750, and GSM6997752). **a** UMAP visualization of integrated hepatocytes from (top) Control and (bottom) FALD patient samples, colored by condition (Control: orange; FALD: purple). **b** UMAP visualization showing levels of *TET1* expression by the hepatocytes in (**a**). **c** Sina plots comparing the gene set scores of the Cluster 01 signature between Control and FALD hepatocytes. The distributions of Cluster 01 signature scores across individual hepatocytes were compared using a two-sided Wilcoxon rank-sum test. **d** Correlation between the expression of the YAP1–TEAD-target gene set and the Cluster 01 signature in human liver cancers from TCGA (*n* = 364). Correlation was assessed using Spearman’s rank correlation coefficient with a two-sided test. **e**–**g** Overall survival was analyzed using the Kaplan–Meier method, and differences between the high- and low-expression groups were assessed using the log-rank test. **e** Survival rates of HCC patients with high (*n* = 182) or low (*n* = 182) expression of YAP1-TEAD target genes in their liver cancers. **f** Survival rates of HCC patients with high (*n* = 179) or low (*n* = 179) expression of *TET1*. **g** Survival rates of HCC patients with high (*n* = 182) or low (*n* = 182) expression of the Cluster 01 signature. **h** Proposed model of canonical DGM-negative HCC induction, namely “epigenetic changes” followed by “DNA mutations”. YAP1-TEAD-TET1-mediated epigenetic reprogramming initially drives the transformation of hepatocytes in the absence of detectable mutations to canonical driver genes (DGMs) by demethylating oncogenes and thereby activating them. This mechanism may operate in at least some FALD-associated HCCs. Subsequently, additional somatic DNA mutations affecting DGMs may accumulate, giving rise to DGM-positive HCCs.

Fontan-associated liver disease (FALD) is associated with the development of HCCs characterized by lower frequencies of canonical HCC DGMs, but the mechanism underlying this association is unknown^8,10^. To determine whether this mechanism involves the HCC-associated YAP1-TEAD-TET1 axis that we identified in our mouse model, we leveraged a recently published transcriptomic atlas of human FALD (GSE223843)^56^ that comprised 20,933 control and 24,921 FALD-derived cells. From this dataset, we extracted hepatocytes based on lineage marker expression (Fig. 8a)^56^. Importantly, we found that *TET1* expression was specifically upregulated in this FALD-specific hepatocyte population (Fig. 8b), mimicking the epigenetic reprogramming seen in our mouse HCC model (Figs. 3b and 5j). Given our finding that Cluster 01 represents the predominant cell type characterized by *Tet1* expression (Fig. 5j), we evaluated Cluster 01 DNA demethylation target oncogenes identified in our mouse model (30 genes; Fig. 5k, l, hereafter referred to as the Cluster 01 signature) in our FALD hepatocytes. Notably, the Cluster 01 signature was significantly enriched in FALD hepatocytes compared to controls (Fig. 8c).

Next, to investigate the prognostic significance of these pathways in human HCCs, we analyzed data from a cohort of HCC patients (*n* = 364) in The Cancer Genome Atlas (TCGA). The expression of YAP1–TEAD target genes correlated significantly with the Cluster 01 signature (Fig. 8d). Lastly, Kaplan–Meier survival analysis revealed that high expression levels of YAP1-TEAD target genes and *TET1* were significantly associated with poor patient prognosis (Fig. 8e, f). High expression of the Cluster 01 signature was also a robust predictor of poor survival (Fig. 8g). Taken together, these results demonstrate that the Cluster 01 signature driven by the YAP1-TEAD-TET1 axis is not only activated in FALD, but can also serve as a valuable prognostic marker for established human HCCs (Fig. 8h).

## Discussion

In this study, we sought to elucidate the molecular mechanisms underlying the initiation of canonical DGM-negative cancers, a phenomenon frequently observed in human malignancies. To this end, we established a mouse model in which activated YAP1 was introduced into hepatocytes in mosaic fashion, followed by genomic, epigenomic, transcriptomic, and spatial transcriptomic analyses. This approach allowed only a short 4-month timeframe for cancer development, a strategy designed to avoid the widespread accumulation of passenger mutations that occurs over longer periods. Using this model, we successfully induced the development of canonical DGM-negative HCCs in male mice and identified the expression of YAP1-TEAD target genes, especially *Tet1*, as well as the expression of a demethylation gene set. Although Wu et al. previously showed that TET1-mediated DNA demethylation downstream of YAP1 activation contributes to liver cancer formation, they did not evaluate DGMs in this study of their model^38^. To our knowledge, ours is the first mammalian model of canonical DGM-negative carcinogenesis shown to be driven by epigenetic alterations that are induced by YAP1 activation.

To investigate the mutational landscape of our HCCs, we conducted WES rather than WGS. The WES approach achieves an approximately 50-fold greater read depth than WGS for the equivalent data, ensuring robust detection of low-frequency somatic mutations. In this study, the average read depth exceeded 330×, which permitted us to identify three canonical DGM-negative HCCs (HCC1, HCC2 and HCC5-2). It should be noted that no common driver mutations were shared across the other samples (HCC3, HCC4 and HCC5-1), ruling out the possibility that a single specific genetic alteration can drive HCC formation in this context. Furthermore, all somatic mutations detected in HCC3, HCC4 and HCC5-1 exhibited relatively low tumor allele frequencies (TAF values ranging from 8.2% to 18.2%; Supplementary Data 4–6) that appeared to be of little functional impact. Although these tumors were not classified as canonical DGM-negative under our stringent criteria, they were clearly not canonical DGM-positive HCCs.

Because we used short-read WES for our work, we cannot completely exclude the possibility that non-coding driver mutations or structural rearrangements may have contributed to the observed tumorigenesis. However, recent pan-cancer whole-genome analyses have shown that non-coding drivers are rare compared to protein-coding drivers^4,57,58^. In a comprehensive analysis of non-coding somatic drivers in whole genomes of 2658 cancers, it was predicted that more than 1475 true driver mutations occurred in the protein-coding sequences of cancer-related genes^4^. In contrast, only 96 driver mutations were estimated to have arisen in promoters (73 attributed to TERT), 22 in 5′ untranslated regions (5′UTRs), and 68 in 3′ untranslated regions (3′UTRs). None of our six HCCs showed elevated *TERT* expression (Supplementary Data 1), and no somatic mutations corresponding to reported non-coding driver candidates^4^ were detected among the WES-captured non-coding exonic regions, including UTRs (Supplementary Fig. 8). We also cannot exclude the possibility that the HTVi method used in our model might have induced plasmid integration into the genome. However, such integration-associated effects were unlikely to be the primary tumorigenic drivers because HTVi delivery of YAP1(WT) did not induce HCC (Fig. 1d). Most importantly, consistent gene expression and activation of DNA demethylation targets were observed in all of our YAP1–TEAD-induced HCCs, and TET1 knockdown attenuated tumorigenesis in our mice. Taken together, these findings indicate that HCC formation in our model is primarily mediated by epigenetic changes driven by a YAP1–TEAD-TET1 axis rather than by changes to the DNA sequence itself, including non-coding mutations and structural rearrangements.

Wu et al. previously utilized a transgenic mouse model in which the drug-inducible expression of active YAP in all hepatocytes led to hepatomegaly and tumorigenesis^38^. By crossing these transgenic mice with *Tet1*-deficient mice, this group demonstrated that the genetic ablation of *Tet1* significantly suppressed both liver enlargement and tumorigenesis. Furthermore, they identified specific downstream targets epigenetically regulated by the YAP1-TET1 axis at Day 10, an early pre-malignant stage. In contrast, we have established a mosaic mouse model of YAP1-induced HCC that enables the analysis of cell-cell interactions well into the process of HCC formation. Consistent with Wu et al.’s results, we found that TET1 knockdown attenuated YAP1-TEAD-induced HCC formation in our model (Fig. 3e). However, in addition to the epigenetic target genes identified by Wu et al., we uncovered additional DNA demethylation genes that were targets only in 4M HCC livers; i.e., much later during tumorigenesis. These temporal differences in DNA demethylation target gene expression likely reflect stage-specific events during the initiation, progression, and maintenance of HCC.

Given that our HCCs arose in a mosaic model (Fig. 1c), we initially hypothesized that the resulting tumors would be composed of a homogeneous cell population. However, contrary to this prediction, our spatial transcriptomic analysis revealed that our HCCs were composed of at least four distinct cell populations (Clusters 01–04). These data indicated the presence of significant intratumoral heterogeneity (Fig. 5d) and underscored the role of epigenetic modification in generating transcriptional diversity^59^. The elevated expression of YAP1-TEAD target genes was maintained across all four clusters despite differentiation or state transitions during tumor progression (Fig. 5f). Furthermore, our single-cell resolution analysis revealed that both *Tet1*-high and *Tet1*-low cells were present within the same tumor (Fig. 5j). This heterogeneity in *Tet1* expression implies that additional, as-yet-unidentified factors are required for the complete activation/function of the YAP1-TEAD-TET1 axis. Of note, the elevated expression of both *Yap1* and *Tead4* in Clusters 01 and 03 suggests the establishment of a positive feedback loop that may serve to sustain the oncogenic state (Fig. 5f and 8h).

Visualization of the intercellular network within the HCC TME confirmed that each cell cluster formed strong interactions with normal non-parenchymal cells (Figs. 6 and  7, and Supplementary Fig. 7). Specifically, cancer cells reprogrammed by the YAP1–TEAD-TET1 axis could actively drive TME remodeling by co-opting classical pathways (e.g., VEGF, SPP1, APP, FN), and by undertaking non-canonical complementary signaling to manipulate non-parenchymal cells for angiogenesis and immune evasion^51,60,61^. Cluster 01 also expressed multiple oncogenes, including SPP1 and APP, which are DNA demethylation targets of the YAP1–TEAD–TET1 axis and have previously been implicated as contributors to TME formation^50,53,54,62,63^. To our knowledge, our study presents the first spatially resolved gene expression map describing intratumoral heterogeneity and the functional interactome of canonical DGM-negative HCCs. Our work thus provides critical insights into the cellular dynamics of HCC formation.

In FALD patients, hemodynamic stress is considered to play a critical role in initiating carcinogenesis^6,64,65^. Recently, Hu et al. reported that hepatocytes near the hepatic central vein undergo metabolic reprogramming as an initial response to hepatic congestion^56^. Although the mechanical stresses caused by hepatic congestion are known to activate the YAP1 pathway^66,67^, the potential involvement of YAP1 in driving FALD-associated HCC has remained unresolved. In our study, we provide data supporting the idea that epigenetic changes induced by activation of the YAP1-TEAD-TET1 axis constitute the molecular basis of FALD-associated HCC. Indeed, our analysis of human FALD scRNA-seq data revealed specific upregulation of *TET1* and Cluster 01 target genes in FALD hepatocytes (Fig. 8b, c). Pertinently, FALD patients develop HCC at a younger age (median 30–34 years) than those with classical HCC^6,9^, leaving insufficient time for the accumulation of somatic driver mutations. This early onset may be one factor explaining the lower frequencies of canonical HCC DGMs reported in FALD-related HCCs^8–10^.

The prognostic value of our Cluster 01 signature in the TCGA cohort, which predominantly comprises DGM-positive cases, points to the broader applicability of our findings (Fig. 8g). In this vein, we propose a model in which HCCs arise due to “epigenetic changes” followed by “DNA mutations” (Fig. 8h). In this scenario, HCCs initiate as a result of YAP1-TET1-mediated epigenetic changes and so are in a canonical DGM-negative state. As these tumors expand over a long period, inherent genomic instability, which is a well-established hallmark of cancer^68^, promotes the accumulation of somatic driver mutations, resulting in HCCs in a canonical DGM-positive state. Furthermore, because YAP1 is frequently activated in a wide range of human solid tumors^14–16^, similar molecular mechanisms may be involved in the development of cancers other than HCC.

## Methods

### Mice

C57BL/6J mice were purchased from Sankyo Laboratory Service Corporation (Tokyo, Japan). Mice were housed under specific pathogen-free conditions at 23 ± 2 °C and 50 ± 10% relative humidity with a 12 h light/dark cycle and free access to water and radiation-sterilized diet products CE-2 diet (CLEA Japan, Tokyo, Japan). Male mice aged 8 weeks were used unless otherwise stated. Mice were acclimatized for at least 7 days before experimental procedures. Mice were assigned to experimental groups randomly. All mouse experiments were approved by the Institutional Animal Care and Use Committee of the Institute of Science Tokyo and were performed in accordance with relevant institutional guidelines and regulations.

### Plasmids and hydrodynamic tail vein injection (HTVi)

For hydrodynamic injection, plasmids (20 µg) were diluted in TransIT-EE Hydrodynamic Delivery Solution (Mirus Bio, Madison, WI, USA) to a volume equivalent to 10% of the mouse’s body weight and injected into the tail vein within 5–6 seconds (s) as previously described^69^. Plasmids utilized in this study were: pLIVE-Myc-YAP1 (WT), pLIVE-Myc-YAP1 (1SA: S127A), pLIVE-Myc-YAP1 (2SA: S127A, S397A), pLIVE-Myc-YAP1 (5SA: S61, 109, 127, 164, 397A), pLIVE-Myc-YAP1 (5SA/WW1, 2*), pLIVE-Myc-YAP1 (5SA/ΔC), pLIVE-Myc-YAP1 (5SA/TEAD*), pLIVE-Myc-KRAS(G12V), pLIVE-Myc-v-Src, and pLIVE-Empty. All plasmids used in this study were generated in-house using standard molecular cloning techniques. The series of YAP1 mutant plasmids, including the 5SA variant and its derivatives, has been described previously^69^.

### TET1 knockdown using shRNA

To perform Tet1 knockdown in mouse liver, we utilized a vector based on recombinant adeno-associated virus serotype 8 (AAV8) (pAAV-CBH-tRFP-shTet1; VectorBuilder). This vector encodes four distinct miR30-based shRNAs targeting *Tet1* (the specific targeting sequences are listed in Supplementary Data 20) plus a TurboRFP reporter under the control of the ubiquitously expressed chromatin opening element (CBh) promoter. C57BL/6J mice (8 weeks old) were injected intravenously via the tail vein with 1 × 10^11^ genome copies (GC) of the viral particles. At 2 weeks post-infection, 40 µg pLIVE-Myc-YAP1(2SA) plasmid was delivered to the liver via HTVi. At 4M post-plasmid injection, livers were harvested and numbers of macroscopic tumors and tumor nodules were counted to assess tumor burden.

### Paraffin histology and immunohistochemistry

Mouse livers were fixed in 4% paraformaldehyde (PFA) for 24 h, processed through an automated tissue processor (Excelsior ES, Thermo Fisher Scientific, Waltham, MA, USA), and embedded in paraffin. Sections (5 µm) were stained with hematoxylin and eosin (H&E) or Picro-Sirius Red using standard methods. For immunohistochemistry (IHC), deparaffinized sections underwent heat-induced antigen retrieval, and endogenous peroxidases were quenched with 0.3% H₂O₂. After blocking with 2.5% normal horse serum, sections were incubated with primary antibodies overnight at 4 °C. For chromogenic detection, sections were subsequently incubated with HRP-conjugated secondary antibodies, and signals were developed using an ABC-HRP kit (Vector Laboratories, Newark, CA, USA) and DAB reagent (Sigma-Aldrich, St. Louis, MO, USA). For fluorescent detection, sections were incubated with appropriate fluorophore-conjugated secondary antibodies and counterstained with DAPI. Antibodies used for IHC are listed in Supplementary Data 21.

### Cryosectioning and immunofluorescence

For immunofluorescence (IF), livers were fixed in 4% PFA for 24 h and cryoprotected in 30% sucrose before embedding in O.C.T. compound (Sakura Finetek, Tokyo, Japan). Cryosections (10 µm) were permeabilized and blocked using 0.1% Triton X-100 and 5% BSA in TBS. Sections were incubated with primary antibodies overnight at 4 °C, followed by incubation with fluorophore-conjugated secondary antibodies and DAPI (nuclear counterstaining). All sections were imaged using a BZ-X710 fluorescence microscope (Keyence, Osaka, Japan). Antibodies used for IF are listed in Supplementary Data 21.

### Quantitative real-time PCR (qPCR)

qPCR was performed as previously described^70^. Briefly, total RNA was extracted using Trizol Reagent (Invitrogen, Carlsbad, CA, USA) and the RNeasy Mini Kit (QIAGEN, Hilden, Germany). cDNA was synthesized with ReverTra Ace qPCR RT Master Mix (Toyobo, Osaka, Japan). qPCR was performed using THUNDERBIRD SYBR qPCR Mix (Toyobo, Osaka, Japan) on a CFX96 Real-Time System (Bio-Rad, Hercules, CA, USA). Thermal cycling conditions were 95 °C for 30 s, followed by 40 cycles of 95°C for 5 s and 60 °C for 30 s. All primer sequences are listed in Supplementary Data 22.

### Western blotting

Western blotting was performed as previously described^71^. Briefly, total protein was extracted using a lysis buffer [50 mM Tris-HCl (pH 7.5), 150 mM NaCl, 1 mM EDTA, 1% Triton X-100, 0.5% sodium deoxycholate, 0.1% SDS, and protease/phosphatase inhibitors]. Tissue samples were homogenized with a POLYTRON homogenizer, and protein concentrations were determined using a BCA Protein Assay Kit (Thermo Fisher Scientific, Waltham, MA, USA). Equal amounts of protein (5 µg) were separated by 8% or 10% SDS-PAGE and transferred to polyvinylidene difluoride (PVDF) membranes (Merck Millipore, Burlington, MA, USA). Membranes were blocked with Blocking ONE (Nacalai Tesque, Kyoto, Japan) for 1 h and then incubated overnight at 4 °C with the following primary antibodies: anti-Tet1 (1:500; Abcam, Cambridge, UK, ab191698) and anti-GAPDH (1:5000; Millipore, Burlington, MA, USA, MAB374). After washing with TBS, membranes were incubated for 1 h with HRP-conjugated secondary antibodies (anti-rabbit, 1:1000, Amersham, Buckinghamshire, UK; anti-mouse, 1:3000, Millipore, Burlington, MA, USA) and developed using West Pico or West Femto chemiluminescent substrates (Thermo Fisher Scientific, Waltham, MA, USA). Signals were acquired with a ChemiDoc MP system (Bio-Rad, Hercules, CA, USA), and band intensities were quantified using ImageJ software (v1.54g, National Institutes of Health, Bethesda, MD, USA).

### RNA-seq

Total RNA was extracted using RNeasy Mini Kits (QIAGEN, Hilden, Germany) according to the manufacturer’s instructions. RNA-sequencing analysis was entrusted to Takara Bio Inc. (Shiga, Japan). The SMART-Seq v4 Ultra Low Input RNA Kit for Sequencing (Clontech, Mountain View, CA, USA), Nextera XT DNA Library Prep Kit (Illumina), and Nextera XT Index Kit v2 (Illumina, San Diego, CA, USA) were used to amplify double-stranded cDNAs and prepare the sequencing library. RNA-seq was performed using a NovaSeq 6000 instrument plus NovaSeq Control Software v1.6.0, Real Time Analysis (RTA) v3.4.4, and Bcl2fastq2 v2.20. Sequence data were analyzed using the DRAGEN Bio-IT Platform v3.6.3 (Illumina) with GRCm38 Release m25 as the reference sequence. Analysis of differentially expressed genes was performed using scores of transcripts per million (TPM).

### Differential gene expression and pathway analysis

Further analysis of RNA-seq data was performed using v1.1 of iDEP (integrated Differential Expression and Pathway), a web-based tool from South Dakota State University (Brookings, SD, USA), as previously described^72^. Expression data, quantified as TPM, were log₂-transformed and normalized. Differentially expressed genes (DEGs) were identified based on a threshold of *P* < 0.05 and a log₂ fold change (FC) ≥ 1, and were visualized using principal component analysis (PCA) and volcano plots. Following hierarchical clustering of DEGs, functional enrichment analysis was conducted using the KEGG, Reactome, and Gene Ontology (GO) databases. A heatmap of the identified DEGs was generated using R (v4.4.2).

### Pathway and gene ontology enrichment analysis

Over-representation analysis was performed on the gene lists from each experimental group to identify functionally enriched pathways and GO terms. The analysis was conducted using the Python library gseapy (v1.1.8) against the KEGG (v2016), GO Biological Process (v2018), and Reactome (v2016) pathway databases. Pathways or terms with a Benjamini–Hochberg adjusted *P* value < 0.05 were considered significantly enriched. For visualization, the top 10 entries from each group, ranked by the ‘Combined Score’, were selected. These results were illustrated as bubble plots generated with seaborn (v0.13.2) and Matplotlib (v3.9.2).

### Whole exome sequencing (WES)

Genomic DNA was extracted using the NucleoSpin Tissue Kit (Macherey-Nagel, Düren, Germany). WES library preparation was performed by Takara Bio Inc. (Shiga, Japan) using the Twist Mouse Exome Panel and associated library preparation kits (Twist Bioscience, South San Francisco, CA, USA). Sequencing was performed on a NovaSeq 6000 system (Illumina). For analysis, reads were mapped to the mouse reference genome (GRCm38/mm25) and processed using the DRAGEN Bio-IT Platform (Illumina, San Diego, CA, USA). Somatic mutations (SNVs and indels) were identified through a comparative analysis of tumor vs. normal liver tissues.

### Gene list overlap analysis

Pairwise intersections among gene lists were quantified using a Python (v3.12.3, Python Software Foundation, Wilmington, DE, USA) script with the pandas library (v2.2.2), following a case-insensitive conversion of gene names. The resulting intersection matrix was visualized as a heatmap using seaborn (v0.13.2) and Matplotlib (v3.9.2). The color scale was logarithmically transformed to accommodate a wide range of values, and each cell was annotated with the overlap count. For single-gene overlaps, the gene name was also specified. Non-coding somatic variants with a tumor allele frequency (TAF) > 0.03 were extracted from WES data and compared with nine WES-assessable non-coding exonic driver candidate elements reported by Rheinbay et al.^4^. Overlap counts were summarized for each tumor sample and candidate element.

### RRBS preparation and sequencing

RRBS analysis was entrusted to Takara Bio Inc. (Shiga, Japan) and Active Motif Inc. (Carlsbad, CA, USA). Briefly, genomic DNA (100 ng) was digested with TaqI (New England Biolabs, Ipswich, MA, USA, R0149) at 65 °C for 2 h followed by digestion with MspI (New England Biolabs, R0106) at 37 °C overnight. Following enzymatic digestion, samples were employed for library generation using the Ovation RRBS Methyl-Seq System (Tecan, Männedorf, Switzerland, 0353-32) following manufacturer’s instructions. Digested DNA was randomly ligated, and, following fragment end repair, bisulfite-converted using the EpiTect Fast DNA Bisulfite Kit (Qiagen, Hilden, Germany, 59824) following the manufacturer’s protocol. After conversion and clean-up, samples were amplified using the Ovation RRBS Methyl-Seq System protocol for library amplification and purification. Libraries were measured using the Agilent 2200 TapeStation System (Agilent, Santa Clara, CA, USA) and quantified using Nanodrop 2000c (Thermo Fisher Scientific, Waltham, MA, USA). Libraries were sequenced on a NovaSeq 6000 instrument (Illumina, San Diego, CA, USA) to generate 75-bp single-end reads.

### DNA methylation analysis

The Illumina adapter sequence was trimmed from the single-end 75 bp reads generated above using Trim Galore (Babraham Institute, Cambridge, UK), followed by custom trimming of library-specific bases. Reads were mapped to the genome using Bismark (version 0.23.0, Babraham Institute, Cambridge, UK) with strict parameters (-N 0, -L 20). PCR duplicates were removed by retaining a single representative read among those with identical mapping coordinates and randomized 6-mer barcodes. Alignment information of the remaining reads was stored in BAM files. Methylation at CpG sites was extracted using Bismark (version 0.23.0) and saved in CpG report format.

### Differential methylation analysis and annotation

CpG report files generated by Bismark as above were used to identify “Differentially Methylated Cytosines” (DMCs) between two sample groups (e.g., Non-Tumor vs. Tumor). First, all CpG sites with a minimum read coverage of 10x in at least two samples were consolidated into a union site list for subsequent statistical testing. Differential methylation at each CpG site was assessed using a Generalized Linear Model (GLM) with a binomial error structure. This model was applied to the counts of methylated and unmethylated reads, with the Non-Tumor or HCC status set as the explanatory variable. The resulting *P*-values were adjusted using the Benjamini–Hochberg method to control the False Discovery Rate (FDR). A CpG site was classified as a DMC if it met two strict criteria: (1) a q-value less than 0.01, and (2) an absolute methylation difference between the groups of ≧20%. The overall distribution of the q-values and methylation differences across the genome was visualized in a Manhattan-style plot. DMCs were mapped to corresponding gene symbols using org.Mm.eg.db.

### Visium HD library preparation and sequencing

Visium HD sample preparation was performed on FFPE liver sections with a DV200 value > 90% (where DV200 is the percentage of RNA fragments >200 nucleotides) according to the manufacturer’s protocol (10x Genomics, Pleasanton, CA, USA, CG000684). Sections (5 µm) were stained with anti-ATP1A1 antibody (Abcam, Cambridge, UK, ab76020) and DAPI before immunofluorescence imaging on a BZ-X710 microscope (Keyence, Osaka, Japan). Following imaging, tissue sections were transferred to Visium HD slides using a CytAssist instrument (10x Genomics). Libraries were prepared and subsequently sequenced on an AVITI platform (Element Biosciences, San Diego, CA, USA).

### Visium HD data processing and analysis

Raw sequencing data were processed using Space Ranger (10× Genomics, Pleasanton, CA, USA) with the mm10-2020-A reference genome, and the output was converted to Zarr format via spatialdata-io (v0.1.dev815+g70f5060). Individual cells were segmented as regions of interest (ROIs) using Cellpose (v3.1.1.1, Janelia Research Campus, Ashburn, VA, USA) with pretrained models, which defined cell boundaries based on ATP1a1 immunofluorescence and nuclei based on DAPI staining. To precisely define the cell boundaries within the regions encompassing both HCC lesions and surrounding tissue, 12,094 cell contours were subsequently manually annotated. These ROI geometries were integrated into a SpatialData object (v0.3.1.dev17+gc2136b3). Gene expression was subsequently aggregated within each ROI boundary to quantify total transcript abundance per cell, with processing accelerated through parallel computing (n_jobs = 20). Individual sample datasets were concatenated into a single AnnData object, preserving sample identity via library keys. Standard quality control was performed, filtering out cells with fewer than 50 genes and genes detected in fewer than 100 cells.

To mitigate technical artifacts from sample preparation and sequencing, batch effects were corrected using the scVI model (v1.3.0, University of California, Berkeley, CA, USA). The model was configured with a variational autoencoder architecture (2 hidden layers, 10 latent dimensions) and a negative binomial gene likelihood, enabling simultaneous batch correction and low-dimensional representation learning. A neighborhood graph (*k* = 5 neighbors) was constructed from the batch-corrected scVI latent representations. This graph was used for cell clustering via the Leiden algorithm (resolution=0.5) and for visualization with UMAP (minimum distance=0.1). Cell types were subsequently identified through manual curation based on canonical marker gene expression. This process identified the following primary cell populations: hepatocytes (6 subtypes), liver sinusoidal endothelial cells (LSECs), Kupffer cells, lymphocytes, cholangiocytes, stellate cells/fibroblasts, HCC areas, and border regions.

To investigate heterogeneity within the malignant regions, a focused sub-analysis was performed on annotated HCC areas. Iterative Leiden clustering (resolution=0.3) was applied to further subdivide these cell populations, identifying four distinct HCC subtypes that were designated Cluster 01 through Cluster 04.

### Region-specific inference of cell-cell communication using CellChat

“Cell-Cell Communication Analysis” intercellular communication networks within the HCC TME were inferred using the R package CellChat (version 1.6.1)^49^ on R version 4.5.0. To define “Regions of Interest” and “Cell Groups”, we analyzed spatial transcriptomics data from a control mouse liver and an HCC-bearing liver. Four regions were defined: (1) Control; (2) Non-tumor area (normal region in HCC-bearing 4M liver); (3) Border area (tumor interface region); and (4) HCC area (tumor core). Separate CellChat objects were constructed for each region. To ensure robust analysis, raw gene expression data were normalized using library size normalization followed by logarithmic transformation (using the normalizeData function). Cells were grouped based on detailed annotations, with T cells and B cells merged into a single “Lymphocytes” group. The mouse database (CellChatDB.mouse) was utilized, targeting the “Secreted Signaling”, “ECM-Receptor”, and “Cell-Cell Contact” pathways. Overexpressed genes and interactions were identified using standard CellChat functions. To characterize region-specific signaling signatures, ligand-receptor pairs were ranked by their maximum communication probability within each functional category. The top 20 interactions for “Secreted Signaling,” “ECM-Receptor,” and “Cell-Cell Contact” were extracted and visualized as bar plots. The total interaction strength was computed using the aggregateNet function to aggregate probabilities across all ligand-receptor pairs. Visualizations were generated using the netVisual_circle function and ggplot2. To facilitate direct quantitative comparison across regions, visualization parameters—such as bar lengths and edge widths in aggregated circle plots—were unified based on the global maximum values observed across all four groups. For visualizations of individual ligand-receptor pairs, edge widths were scaled relative to the maximum communication probability specific to that pair across all samples.

### FALD single-cell RNA-seq analysis

We re-analyzed a public single-cell RNA sequencing dataset (GSE223843) comprising two control liver samples (Control 1: GSM6997741; Control 3: GSM6997744) and four FALD samples (Fontan 1: GSM6997746; Fontan 2: GSM6997748; Fontan 3: GSM6997750; Fontan 4: GSM6997752). Raw data were processed using Seurat, filtering out low-quality cells (>5% mitochondrial reads, <300 detected genes, or UMIs outside 800–20,000). Unsupervised clustering classified all cells into 12 distinct clusters based on gene expression profiles. Hepatocytes were identified based on positive marker expression and the absence of non-parenchymal cell markers (e.g., those of immune cells, fibroblasts, or endothelial cells). Accordingly, nine clusters (Clusters 0, 1, 2, 3, 5, 7, 8, 9, and 11) were extracted for downstream analysis. To identify robust transcriptomic changes associated with FALD, extracted hepatocytes were split by experimental condition (Control vs. FALD) and integrated using Canonical Correlation Analysis (CCA) in Seurat v4. Module scores for TET1 and specific DNA demethylation target gene signatures were calculated using the AddModuleScore function.

### TCGA data analysis

Analyses of TCGA RNA expression, correlation and overall survival data were performed with GEPIA2. Spearman’s correlation coefficient analysis was used to calculate gene expression relationships between target genes.

### Statistical analyses

Analyses of statistical significance were performed using GraphPad Prism 8 (GraphPad Software, San Diego, CA, USA). Student’s *t* test was used for comparisons between 2 groups. One-way analysis of variance (ANOVA) was used for comparisons among 3 or more groups. Two-way ANOVA was used for 2-factor analysis. Data were considered statistically significant at *P* < 0.05.

### Reporting summary

Further information on research design is available in the Nature Portfolio Reporting Summary linked to this article.

## Supplementary information

Transparent Peer Review file

Supplementary Figs. 1-8

Description of Additional Supplementary files

Supplementary Data 1

Supplementary Data 2

Supplementary Data 3

Supplementary Data 4

Supplementary Data 5

Supplementary Data 6

Supplementary Data 7

Supplementary Data 8

Supplementary Data 9

Supplementary Data 10

Supplementary Data 11

Supplementary Data 12

Supplementary Data 13

Supplementary Data 14

Supplementary Data 15

Supplementary Data 16

Supplementary Data 17

Supplementary Data 18

Supplementary Data 19

Supplementary Data 20

Supplementary Data 21

Supplementary Data 22

Reporting Summary

## Data Availability

All high-throughput sequencing data generated in this study have been deposited in the DDBJ Sequence Read Archive (DRA) under the BioProject accession number PRJDB35548. The Visium HD data have also been deposited in the Genomic Expression Archive (GEA) under the GEA Accession ID E-GEAD-1095. The processed data corresponding to the RNA-seq, RRBS, and WES analyses are also provided as Supplementary Data. The source data are provided in Supplementary Data 1 for Figs. 1 and 3, Supplementary Data 2–8 for Fig. 2, Supplementary Data 9–14 for Fig. 4, Supplementary Data 15–16 for Fig. 5, and Supplementary Data 17–19 for Fig. 7. Additional information is available from the corresponding authors upon reasonable request, with Yoshimi Okamoto-Uchida responsible for responding to such requests.
