# Supplementary material for: YAP1 induces hepatocellular carcinoma via DNA demethylation rather than by canonical driver gene mutations
Source: Commun Biol. 2026 Sep 17;9:1198. doi: 10.1038/s42003-026-10882-w (PMC13586348; doi:10.1038/s42003-026-10882-w)
Supplement: Supplementary file 1 [file 42003_2026_10882_MOESM1_ESM.pdf]

nature portfolio

Peer Review File

YAP1 induces hepatocellular carcinoma via DNA demethylation rather than by canonical driver gene mutations

Corresponding Author: Dr Yoshimi Okamoto-Uchid, Dr Teppei Shimamura, and Dr Hiroshi Nishina

This file contains all reviewer reports in order by version, followed by all author rebuttals in order by version.

Attachments originally included by the reviewers as part of their assessment can be found at the end of this file.

Version 0:

Reviewer comments:

Reviewer #1

(Remarks to the Author)
In this manuscript, the authors develop and characterize a mouse model of hepatocellular carcinoma (HCC) driven by transgenic expression of an activated form of YAP in hepatocytes. Comprehensive molecular profiling of HCCs generated with this model system is described, including whole exome sequencing, RNA-Seq, bisulfite sequencing, and single-cell spatial transcriptomics. The authors provide data supporting a model in which activated YAP leads to dysregulation of chromatin modifying and DNA methylating/demethylating machinery (most notably Tet1), leading to genome-wide effects on gene expression that promote neoplastic transformation. YAP1-TEAD targets and Tet1-demethylated gene sets are defined and shown to associate with survival outcomes in human HCC and mesothelioma cohorts. Overall, the manuscript is written in clear and concise language, and it covers an area of molecular cancer cell signaling that is timely and of broad interest to cancer researchers and clinicians. There are, however, several issues (detailed below) that should be addressed prior to acceptance for publication. Most importantly, language concerning the lack of “driver gene mutations” in patient cohorts and experimental HCCs should be softened, as it is in some cases inaccurate and in others not possible to conclude definitively based on available data. The model described is interesting and valuable in its own right but should not be described as free from driver gene mutations.

Major Issues

1. Claims of tumors lacking “driver gene mutations” need to be removed, revised, or significantly qualified. This is a major theme throughout the manuscript and even included in the title but is, in my opinion, unjustified. Several reasons are provided below.
a. The mouse model utilized to generate HCCs is driven by expression of a transgenic YAP construct that has been engineered with serine-to-alanine mutations that render it unaffected by phosphorylation-based regulatory mechanisms that would otherwise decrease its signaling activity. At a fundamental level, these tumors are essentially induced by “driver gene mutations” in the YAP transgene, which is expressed from a promoter with very high activity in the liver (Alb). As the authors state, mutations in YAP are very rare in human cancer, though activation of the pathway is fairly common. While the transgene is meant to mimic YAP-TEAD activation that can occur downstream of fibrosis or other disease states, it still represents a mutant form of YAP that is essential to the model. Notably, a wild-type YAP transgene delivered the same way did not produce tumors in any of the 22 mice listed in Table 1.
b. The hydrodynamic tail vein injection method used to deliver the YAP transgene results in stable incorporation of recombinant DNA into the genome of hepatocytes. Transgene integration can cause insertional mutagenesis, depending on integration sites. As integration sites were not assessed, it is possible that insertional mutagenesis could contribute to tumor formation. There is precedent for mouse tumors, including HCC, being driven by transgene-induced insertional mutation (e.g. PMID 17656716).
c. Although a great deal of genome-level characterization is provided for the HCC samples, it is based on short-read sequencing. No assessment of structural rearrangements is performed, so it is possible that this type of variation contributes to tumorigenesis in the model.
d. The absence of cancer-promoting genetic aberrations in tumor samples is much more difficult to definitively demonstrate

than their presence. Methodologies utilized may not be able to detect certain types of mutations (e.g. short-read sequencing cannot adequately assess structural rearrangements). Sampling bias may cause mutations to be missed. The absence of detectable mutations in known driver genes, as limited by methodologies utilized and depth of analysis, is not the same as a lack of driver gene mutations in a sample.
e. References to human tumors lacking driver gene mutations are discussed in the Introduction on page 5, with two main studies cited (References 3-4). Additionally, DGM-negative cancers are described as a "phenomenon often observed in human malignancies", which is not accurate based on the data provided.
i. Reference 3 does not make a claim that a subset of tumors analyzed lacked driver gene mutations. This interpretation by the authors of the current manuscript appears to come from the oncoplot shown in Figure 1b, which lists mutations in specific genes identified in the patient cohort and shows a group of 38 samples for which none of these mutations were detected. Supplemental Table 1b provides more comprehensive data on all 494 analyzed HCCs. Among the 38 samples for which no mutations were detected in the genes shown in the oncoplot, there are over 1,500 instances of structural variations, chromothripsis, kataegis, altered ploidy, and extrachromosomal DNA that could affect activity of cancer-promoting genes.
ii. In Reference 4, "driver genes" are defined relatively narrowly as known promoters of lung cancer. The authors of the current manuscript highlight the finding that "19% of these tumors in never-smokers had no detectable DGMs". This number is based on data shown in Figure 2 of Reference 4, for which "driver genes" were defined as "known activating mutations or fusions in RTK/RAS/RAF pathway genes". By this same classification, 51.5% of tumors from smokers fall in the same category, and it is well accepted that smoking-associated lung cancer is driven by accumulated mutations. Data presented in Figure S2 of Reference 4 uses a broader definition of "drivers" as "LUAD-related genes", finding alterations in 94% of tumors from never-smokers. This definition is still too narrow to claim absence of driver gene mutations, and other types of mutations (e.g. structural variations) were not comprehensively assessed.

2. Only protein-altering mutations were considered in the analysis depicted in Figure 3. It is unclear why this requirement was implemented when identifying "driver gene mutations". There are many examples of non-coding alterations known to be drivers of tumorigenesis (e.g. those analyzed in Reference 3).

3. From the single-cell spatial transcriptomics analysis, four HCC-specific clusters were identified. Clusters 1-3 each expressed HCC markers, yet only Clusters 1 and 3 expressed Yap1 and its downstream targets, according to wording on page 16, lines 264-267. Does this finding suggest non-cell-autonomous effects of the YAP transgene in Cluster 2 cells? Is there an alternative interpretation for this cluster of HCC cells that don't express Yap1 and its target genes? More discussion is warranted on this topic.
a. In the Discussion section, it is stated that "the same YAP1-TEAD target genes" are expressed across Clusters 1-4, which seems to contradict with the main text.

4. Figure 7a,e display p-value=0 for gene set correlations. A p-value of zero is not possible. As with other figures in the manuscript "$p < x$" should be used instead of $p = 0$.

Minor Issues

1. TAZ is not mentioned at all in the manuscript. YAP and TAZ function at the same mechanistic level in the Hippo signaling pathway. They are often treated as interchangeable/redundant mediators of Hippo signaling. While this characterization is an oversimplification, it would be beneficial to include some discussion of TAZ, at least in the Introduction and/or Discussion section. Is it expected that transgenic expression of active TAZ would produce a similar model of HCC?

2. In Table 1, several constructs are shown that are not described in the manuscript. In particular, the 5SA/WW1,2* construct (which according to Reference 37 is defective in binding transcription factors, such as p73) drives robust HCC formation in all mice analyzed. What is the interpretation of this result? Any of the constructs shown in Table 1 should be discussed in the text of the manuscript.

3. Page 10, lines 151-153: The authors discuss significantly upregulated and downregulated genes identified by RNA-Seq. It would be valuable to perform gene set enrichment analysis (GSEA) to determine whether YAP-TEAD target gene sets are significantly enriched in tumors versus normal liver samples. Several such gene sets have been described (e.g. in PMID 38473214).

4. Did the authors analyze RNA-Seq data for altered splicing or RNA editing in HCCs versus normal liver samples? This data could be informative.

5. Figures 4b and 5e show data from 11 HCCs and 10 non-tumor samples, while Figure 2e-g only shows the 6 HCCs and 5 non-tumor samples that were the focus of most of the manuscript. It is unclear which additional samples were utilized for the analyses in Figures 4-5.

6. Page 14, Line 222: It is unclear what is meant by a fold-change of $\leq 1/1.5$.

7. Page 16, Lines 260-262: Do any of the gene expression signatures identified in HCC clusters 1-4 overlap with findings from the bulk sequencing analysis?

8. In Figure 1c-d, it is unclear whether an anti-YAP antibody (as used in Figure 1c) or an anti-Myc antibody was used for immunofluorescence. This point is important, as it determines whether total YAP or only transgenic YAP is detected.

9. In Figure 6m, it would improve clarity if a border could be drawn around the portion of the tissue section that is histologically HCC.

10. Page 18, lines 301-303: The authors suggest that the gene expression signature may be applicable to additional cancer types. It would be simple to do similar assessments as shown in Figure 7 on additional TCGA datasets.

11. Page 19, Lines 307-308: The statement “YAP1 was introduced into single hepatocytes, followed by mosaic analysis” is strangely phrased. Something like “YAP1 was introduced mosaically into hepatocytes” would be more clear.

Reviewer #2

(Remarks to the Author)
In this manuscript, Misaki Kosaka et.al reported a HCC model induced by sustained YAP1 activation was successfully established and found that YAP1–TEAD–TET1 axis as a possible cause of DGM-negative HCC formation,and uncoverd a potential molecular mechanism that may be applicable to common human cancers.
My main criticism of the manuscript relates to the fact that YAP1–TEAD–TET1 axis drives liver cancer growth have to be reported (Wu, B.K et al Nat Genet, 2022). And the data seem like to be preliminary and need to be further analyzed. It may be necessary to address whether a novel mechanism discovered by the authors, although the other novel targeted genes were identified except YAP1–TEAD–TET1 axis. And how and why other novel targeted genes regulating HCC tumor growth showed be ananlyzed and discussed in the manuscript. Overall, the manuscript does not meet the high quality of the journal, I recommend the manuscript currently could not be published.
Major points:
1. In the manuscript, gender difference was observed in that female mice transfected with the same plasmids developed liver cancer less frequently. The potential roles of ER and AR in the hepatocytes for HCC progession need to be considered in future.
2. The potential clinical features in human liver cancer related to DGM-negative HCCs should be analyzed and discussed.

Reviewer #3

(Remarks to the Author)
In this study, Kosaka et. al. aimed to understand how hepatocellular carcinomas (HCC) lacking driver gene mutations (DGMs) may arise. YAP1 activation is frequently observed in HCC patients with poor prognosis yet is typically not mutated in these cancers. The authors hypothesize a 2-step process in which fibrosis/inflammation induces YAP1 activation which subsequently activates pro-survival, oncogenic pathways with the transcription factor TEAD through DNA demethylation of YAP1-TEAD target genes. Using CCl4 treatment in C57BL/6J mice, liver fibrosis was induced and coincided with expression of YAP1 over a period of 15 weeks. In a separate model, YAP1(2SA) sustained expression induced HCC, both DMG(+) and DMG(-), in male mice over a period of 4 months while WT YAP1 did not. Profiling of the resultant HCC transcriptomes revealed conserved gene expression profiles among the HCC tumors and demonstrated upregulation of epigenetic modifiers, including a known target of YAP1-TEAD, the DNA demethylase Tet1. RRBS DNA methylation analysis revealed both hypermethylation and hypomethylation of promoter CpG sites, with demethylation correlating with upregulation of several target genes. Spatial transcriptomics of a normal and YAP1-TEAD induced HCC revealed cell clusters with unique gene expression profiles, with Cluster01 expressing YAP-TEAD target genes, Tet1, and the identified DNA demethylation target genes. Indeed, these markers demonstrate poor prognosis in liver cancer patients.

While the end conclusions of this work agree with published literature regarding YAP1 activation in HCC, the observations made are correlative rather than causative. Indeed, the 2-step hypothesis is uncoupled experimentally and does not support the hypothesis that environmentally induced YAP1 activation promotes the development of DGM- HCC.

1. In Figure 1, while CCl4 induced fibrosis, it is unclear from the images presented if YAP1 is indeed adjacent to the fibrotic tissue. The authors state at Line 110 “robust upregulation and clear nuclear accumulation of YAP1”, yet overexpression of YAP1 is observed in the cytoplasm as well as the nucleus. Given that YAP1 cellular localization is dependent on phosphorylation, the overexpression of YAP1 in the cytoplasm should also be considered.

2. No definition for the consequence of the YAP1 (S2A) mutation is provided other than at Line 120 – “constitutive YAP1 activation”. Why do these mutations cause constitutive YAP1 activation? There is no reference provided for this statement. Published literature provides an explanation regarding phosphorylation of these sites, and that mutation of Serine127 prevents sequestering of YAP1 to the cytoplasm. Indeed, this highlights a disconnect between the model for Step #1 versus Step #2 as cytoplasmic YAP1 is observed in the model for Step #1 while the model in step #2 enforces YAP1 localization to the nucleus.

3. The authors hypothesized that YAP1 activation induces DGM- HCCs. While DGM- HCCs are observed in the model, so are DGM+ HCCs. The conclusion regarding this data should be modified as it appears YAP1 (S2A) induces HCC, both with and without DGMs.

4. Line 135, “AFP, CK19, Ki67 or aSMA showed these liver cancers were HCCs”. No references are provided for what these markers indicate, and it is not apparent to a more general audience that may not be experts in liver cancer.

5. Several of the epigenetic modifiers profiled as upregulated by gene expression are cell cycle regulated genes (such as the maintenance methyltransferase Dnmt1). As shown in Figure1d, HCC has a higher proliferative index (Ki67 staining). This is validated in the spatial transcriptomics as Cluster03 demonstrates the highest expression of cell proliferation genes (Fig 6h) coinciding with expression of DNA methylation modifiers (Figure 6j). Normalization of gene expression by proliferative index should be considered to determine if this is indeed “overexpression” in HCC or a consequence of increased cell proliferation relative to the controls.

6. Line 220 – “Our analysis focused on 51,414 promoter regions (out of 122,460 total)”. This statement is confusing as that is more promoters than are observed in the mouse genome. Figure 5 indicates this is CpG sites within promoters. This point needs clarification.

7. Previous work conducted on the YAP1-TEAD-TET1 axis in HCC demonstrated demethylation at enhancers for modulating gene expression rather than promoters (PMID: 35835915); however, the analysis for this work was focused on promoters. If RRBS coverage is available, enhancers should be considered in this analysis as well.

8. “Control” livers are not discussed until line 243 yet is used throughout the gene expression analysis with no definition provided for the source of these controls. “Control” livers need to be clarified in the text.

Version 1:

Reviewer comments:

Reviewer #1

(Remarks to the Author)
In this revised manuscript, the authors have adequately addressed nearly all of the issues raised in my review of the initial submission. In some cases, however; some slight additional modifications should be made, as detailed below. Additionally, the authors have added a substantial amount of new data and discussion to the revised manuscript. Based on this new information, one major issue and 5 minor issues should be addressed prior to acceptance for publication.

Remaining Major Issues (Initial Submission)

1. Claims of tumors lacking “driver gene mutations” need to be removed, revised, or significantly qualified. This is a major theme throughout the manuscript and even included in the title but is, in my opinion, unjustified. Several reasons are provided below.
a. The mouse model utilized to generate HCCs is driven by expression of a transgenic YAP construct that has been engineered with serine-to-alanine mutations that render it unaffected by phosphorylation-based regulatory mechanisms that would otherwise decrease its signaling activity. At a fundamental level, these tumors are essentially induced by “driver gene mutations” in the YAP transgene, which is expressed from a promoter with very high activity in the liver (Alb). As the authors state, mutations in YAP are very rare in human cancer, though activation of the pathway is fairly common. While the transgene is meant to mimic YAP-TEAD activation that can occur downstream of fibrosis or other disease states, it still represents a mutant form of YAP that is essential to the model. Notably, a wild-type YAP transgene delivered the same way did not produce tumors in any of the 22 mice listed in Table 1.

We thank the reviewer for this important comment. In this study, we used a mutant form of the oncogene YAP1, YAP1(2SA), to induce hepatocellular carcinoma (HCC) in mice. The rationale for using this system was to induce HCC within a short period (4 months). In contrast, sustained activation of endogenous YAP1–TEAD by mechanical stress (without genetic alteration) requires a prolonged period to induce HCC. Indeed, as the reviewer points out, expression of wild-type (WT) YAP1 did not induce HCC even after 12 months. Therefore, we consider that this experimental system using YAP1(2SA) represents an appropriate model to mimic the results of sustained YAP1– TEAD activation that would otherwise require a long time to induce malignancy.

I agree that the model is appropriate and justified. Transformation and tumor growth is, however, still induced by expression of a driver gene harboring an activating mutation. The authors’ modifications to the text are appreciated and have sufficiently clarified and qualified conclusions in almost all sections.

There are some remaining instances where “DGM-free” should be changed to “canonical DGM-free”, as listed below.

Page 13, Line 11
Page 26, Lines 3-4, 9, 11, 17
Page 30, Line 8

b. The hydrodynamic tail vein injection method used to deliver the YAP transgene results in stable incorporation of recombinant DNA into the genome of hepatocytes. Transgene integration can cause insertional mutagenesis, depending on integration sites. As integration sites were not assessed, it is possible that insertional mutagenesis could contribute to tumor formation. There is precedent for mouse tumors, including HCC, being driven by transgene-induced insertional mutation (e.g. PMID 17656716).

We agree with the reviewer on this important point. Genes introduced by hydrodynamic tail vein injection (HTVi) can be integrated into the genome, and insertional mutagenesis may occur depending on the integration sites. However, when WT YAP was introduced in this manner, no tumor formation was observed at 6 months (0/10 mice), and no tumors were detected even at 12 months (0/3 mice) (revised Fig. 1d). In contrast, when YAP1(2SA) was introduced, tumor formation was observed in all mice at 6 months (3/3 mice). Based on these results, we consider that tumor formation in this model is unlikely to be driven by insertional mutagenesis.

The delivery method depends on plasmid integration for maintained expression of the transgenic cargo. A lack of tumor formation with WT YAP constructs in 10 mice does not exclude the possibility that transgene insertions in YAP1(2SA)-injected mice produce oncogenic effects that cooperate with the transgene, which is clearly the primary driver of tumorigenesis. I'm not implying that this is likely to be the case, but it's a possibility that cannot be excluded.

Some mention of the nature of the HTVi model (i.e. genomic integration of injected plasmids) should be included somewhere in the manuscript.

2. Only protein-altering mutations were considered in the analysis depicted in Figure 3. It is unclear why this requirement was implemented when identifying "driver gene mutations". There are many examples of non-coding alterations known to be drivers of tumorigenesis (e.g. those analyzed in Reference 3).

We would like to clarify our rationale for focusing on protein-altering mutations. In our study, somatic mutation analysis was performed using whole-exome sequencing (WES). Compared with whole- genome sequencing (WGS), WES enables greater sequencing depth for a given sequencing capacity and is therefore suitable for sensitive detection of low-frequency mutations. In our analysis, sequencing was performed at a mean depth of over 330×, allowing robust evaluation of protein-altering mutations in known cancer-related genes. As the reviewer points out, non-coding alterations can contribute to tumorigenesis. However, recent large-scale pan-cancer whole-genome analyses have shown that such alterations are less frequent than protein-coding driver mutations. For example, a comprehensive analysis of 2,658 cancer genomes (Rheinbay et al., Nature, 2020, Revised reference # 4) identified more than 1,475 driver mutations in protein-coding regions, whereas substantially fewer were identified in non-coding regions. The latter included 96 in promoters (73 in the TERT promoter), 22 in 5′ untranslated regions (5′UTRs), and 68 in 3′ untranslated regions (3′UTRs). Although non-coding regions were not directly analyzed in our study, we did not observe increased expression of TERT, a representative non- coding driver-associated gene, in the six HCCs analyzed (Supplementary Table 1). Given these considerations, we feel we are justified in focusing on protein-altering mutations in our study. Nevertheless, because we cannot exclude the possibility that non-coding alterations may have occurred, we have added the following sentences to our revised Discussion:
"Because we used short-read WES for our work, we cannot completely exclude the possibility that non-coding driver mutations or structural rearrangements may have contributed to the observed tumorigenesis. However, recent pan-cancer whole-genome analyses have shown that non-coding drivers are rare compared to protein-coding drivers 4,56,57. In a comprehensive analysis of non-coding somatic drivers in whole genomes of 2,658 cancer cells, it was predicted that more than 1,475 true driver mutations occurred in the protein-coding sequences of cancer-related genes 4. In contrast, only 96 driver mutations were estimated to have arisen in promoters (73 attributed to TERT), 22 in 5′ untranslated regions (5′UTRs), and 68 in 3′ untranslated regions (3′UTRs). None of our six HCCs showed elevated TERT expression (Supplementary Table 1), and consistent gene expression and activation of DNA demethylation targets were observed in all of our YAP1–TEAD-induced HCCs. Furthermore, TET1 knockdown suppressed tumorigenesis in our mice. Taken together, these findings indicate that HCC formation in our model is primarily mediated by epigenetic changes driven by a YAP1–TEAD-TET1 axis rather than by changes to the DNA sequence itself, including non- coding mutations or structural rearrangements." （Discussion section, Page 27, Lines 7- Page 28, Lines 4）

The authors' point is well taken. However, WES data typically includes coverage of non-coding exons (i.e. UTRs).

If that data is available, it should be included in the mutation analysis.

3. From the single-cell spatial transcriptomics analysis, four HCC-specific clusters were identified. Clusters 1-3 each expressed HCC markers, yet only Clusters 1 and 3 expressed Yap1 and its downstream targets, according to wording on page 16, lines 264-267. Does this finding suggest non-cell-autonomous effects of the YAP transgene in Cluster 2 cells? Is there an alternative interpretation for this cluster of HCC cells that don't express Yap1 and its target genes? More discussion is warranted on this topic.
a. In the Discussion section, it is stated that "the same YAP1-TEAD target genes" are expressed across Clusters 1-4, which seems to contradict with the main text.

We apologize for this inconsistency between the main text and the Discussion. In our revised manuscript, we have corrected the description to clarify that YAP–TEAD target gene expression was upregulated across all HCC clusters (Clusters 01–04) compared with normal hepatocyte clusters (Hepatocytes 1–6). Therefore, there is no need to discuss non-cell-autonomous effects in Cluster 02.

I was initially confused, because Figure 5f (previously 6i) shows very low Yap1 expression in Cluster 02 and Cluster 04, which I interpreted as a lack of transgene expression. I realize now that the transgene encodes human YAP1 (as indicated by capitalization in the manuscript and designation in the cited reference), which would generally not map to the mouse genome based on the degree of exact sequence homology. However, given that detected Yap1 expression is lower in Cluster 02 than in the hepatocyte control, the statement on Page 20, Line 9 regarding Yap1 expression is inaccurate.

This statement should be revised to reflect the experimental data

New Major Issue

1. In the Introduction, References 8 and 9 are used to support the statement "In FALD patients, the liver is subjected to persistent hemodynamic stress due to chronically elevated central venous pressure, leading to the development of both intrahepatic cholangiocarcinoma (ICC) and HCCs that are canonical DGM-negative". This statement is not supported by the references. Reference 8 describes molecular analysis of 5 FALD-associated liver tumors. In all 5 tumors, mutations altering canonical cancer driver genes are reported, including variants in MAP2K2, NRAS, GNAS, MSH3, CTNNB1, NOTCH1, and others. In many cases, the same genes are recurrently mutated across the small cohort. The basis for the authors' statement regarding a lack of canonical DGMs is unclear. Reference 9 is a Review article that mentions genetic/molecular profiling of FALD-associated liver cancer only by referencing Reference 8. Another reference (PMID 41587344, not cited here) performed WGS and transcriptome profiling on liver tumors from 10 FALD patients, finding rampant mutation and alteration of cancer driver genes.

a. On Page 24, Line 1, a similar statement with the same references is included. Reference 55 also includes analysis of FALD-associated liver cancer samples, but it is transcriptomics based and makes no mention of a paucity of canonical driver gene mutations.
b. Page 25, Line 6 also references FALD-associated HCC as "canonical DGM-negative".
c. Page 31, Line 4 also references a "paucity of canonical DGMs" in FALD-associated liver cancer.

New Minor Issues

1. Page 16, Lines 13-14. The statement "These results indicate that TET1 is essential for YAP1-TEAD-induced HCC formation." is too strong, given the supporting evidence. In the Tet1 knockdown cohort, 5 of 11 mice still developed liver tumors. If the tumors in this cohort have restored levels of Tet1, which was not assessed, the statement is justified. Otherwise, it's clear that the activated YAP1 transgene can still drive tumor formation in the face of Tet1 knockdown, just with lower penetrance and multiplicity.

2. Page 19, Line 3; Page 20, Line 5; Page 21, Line 18; Page 29, Line 1. It's unclear what experimental evidence supports the conclusion that HCCs induced in this model system are derived from expansion of a single progenitor hepatocyte. Constitutively-active YAP seems to be a strong oncogenic driver, so it seems plausible that multiple hepatocytes that initially integrated the transgenic plasmid following hydrodynamic injection could clonally expand and contribute to the same tumor mass, even given the mosaic nature of the model. Clonality could be assessed by mapping insertion sites for the transgenic plasmid. I don't think that's necessary for this study, but without direct evidence conclusions about monoclonality can't be made.

3. The "4M" designation should be clarified throughout the manuscript, particularly when compared to the "Day 10" data. "Day 10" data is from the Wu et al. study's pre-malignant samples, while "4M" data is from the current study's HCC samples. Simply referring to these data sets as "Day 10" and "4M" does not provide the adequate context that one is tumor and the other is non-tumor. I recommend changing "4M" to "4M_HCC" throughout the manuscript, as was done for Fig. 5B.

4. Page 26, Lines 10-12. Claims that the current model is the "first mammalian model of DGM-negative carcinogenesis shown to be driven by epigenetic alterations that are induced by YAP1 activation" are undercut somewhat by Reference 37, which is accurately described on Page 28, Lines 5-10. Given that Wu et al. demonstrated YAP1 activation-driven HCC in mice that was dependent on Tet1 and associated with epigenetic alteration of Yap1 targets, some additional clarification of would be helpful. Is the statement based on the lack of detected canonical DGMs in the current study, which Wu et al. didn't assess?

5. Page 59, Line 8. The Fig. 4 legend should have the word "promoter" removed from this line, as the authors extended analysis beyond promoter regions for the revised manuscript.

Reviewer #2

(Remarks to the Author)
The authors have addressed all the comments.The quality of the manuscript was significantly improved.

Reviewer #3

(Remarks to the Author)
The authors have provided a substantially improved manuscript. All of my concerns have been addressed sufficiently, and I now approve publication of this work.

Version 2:

Reviewer comments:

Reviewer #1

(Remarks to the Author)
The authors have addressed all of the issues raised during review. Their efforts have significantly improved clarity of the manuscript and are appreciated. I recommend publication at this stage.

Open Access This Peer Review File is licensed under a Creative Commons Attribution 4.0 International License, which permits use, sharing, adaptation, distribution and reproduction in any medium or format, as long as you give appropriate credit to the original author(s) and the source, provide a link to the Creative Commons license, and indicate if changes were made.
In cases where reviewers are anonymous, credit should be given to 'Anonymous Referee' and the source.
The images or other third party material in this Peer Review File are included in the article's Creative Commons license, unless indicated otherwise in a credit line to the material. If material is not included in the article's Creative Commons license and your intended use is not permitted by statutory regulation or exceeds the permitted use, you will need to obtain permission directly from the copyright holder.
To view a copy of this license, visit https://creativecommons.org/licenses/by/4.0/

Point-by-Point Rebuttal to the Reviewers for Manuscript # COMMSBIO-25-7657-T

Reviewer #1 (Remarks to the Author):

In this manuscript, the authors develop and characterize a mouse model of hepatocellular carcinoma (HCC) driven by transgenic expression of an activated form of YAP in hepatocytes. Comprehensive molecular profiling of HCCs generated with this model system is described, including whole exome sequencing, RNA-Seq, bisulfite sequencing, and single-cell spatial transcriptomics. The authors provide data supporting a model in which activated YAP leads to dysregulation of chromatin modifying and DNA methylating/demethylating machinery (most notably Tet1), leading to genome-wide effects on gene expression that promote neoplastic transformation. YAP1-TEAD targets and Tet1-demethylated gene sets are defined and shown to associate with survival outcomes in human HCC and mesothelioma cohorts. Overall, the manuscript is written in clear and concise language, and it covers an area of molecular cancer cell signaling that is timely and of broad interest to cancer researchers and clinicians. There are, however, several issues (detailed below) that should be addressed prior to acceptance for publication. Most importantly, language concerning the lack of "driver gene mutations" in patient cohorts and experimental HCCs should be softened, as it is in some cases inaccurate and in others not possible to conclude definitively based on available data. The model described is interesting and valuable in its own right but should not be described as free from driver gene mutations.

Major Issues

1. Claims of tumors lacking "driver gene mutations" need to be removed, revised, or significantly qualified. This is a major theme throughout the manuscript and even included in the title but is, in my opinion, unjustified. Several reasons are provided below.

a. The mouse model utilized to generate HCCs is driven by expression of a transgenic YAP construct that has been engineered with serine-to-alanine mutations that render it unaffected by phosphorylation-based regulatory mechanisms that would otherwise decrease its signaling activity. At a fundamental level, these tumors are essentially induced by "driver gene mutations" in the YAP transgene, which is expressed from a promoter with very high activity in the liver (Alb). As the authors state, mutations in YAP are very rare in human cancer, though activation of the pathway is fairly common. While the transgene is meant to mimic YAP-TEAD activation that can occur downstream of fibrosis or other disease states, it still represents a mutant form of YAP that is essential to the model. Notably, a wild-type YAP transgene delivered the same way did not produce tumors in any of the 22 mice listed in Table 1.

We thank the reviewer for this important comment. In this study, we used a mutant form of the

oncogene YAP1, YAP1(2SA), to induce hepatocellular carcinoma (HCC) in mice. The rationale for using this system was to induce HCC within a short period (4 months). In contrast, sustained activation of endogenous YAP1–TEAD by mechanical stress (without genetic alteration) requires a prolonged period to induce HCC. Indeed, as the reviewer points out, expression of wild-type (WT) YAP1 did not induce HCC even after 12 months. Therefore, we consider that this experimental system using YAP1(2SA) represents an appropriate model to mimic the results of sustained YAP1–TEAD activation that would otherwise require a long time to induce malignancy.

b. The hydrodynamic tail vein injection method used to deliver the YAP transgene results in stable incorporation of recombinant DNA into the genome of hepatocytes. Transgene integration can cause insertional mutagenesis, depending on integration sites. As integration sites were not assessed, it is possible that insertional mutagenesis could contribute to tumor formation. There is precedent for mouse tumors, including HCC, being driven by transgene-induced insertional mutation (e.g. PMID 17656716).

We agree with the reviewer on this important point. Genes introduced by hydrodynamic tail vein injection (HTVi) can be integrated into the genome, and insertional mutagenesis may occur depending on the integration sites. However, when WT YAP was introduced in this manner, no tumor formation was observed at 6 months (0/10 mice), and no tumors were detected even at 12 months (0/3 mice) (revised Fig. 1d). In contrast, when YAP1(2SA) was introduced, tumor formation was observed in all mice at 6 months (3/3 mice). Based on these results, we consider that tumor formation in this model is unlikely to be driven by insertional mutagenesis.

c. Although a great deal of genome-level characterization is provided for the HCC samples, it is based on short-read sequencing. No assessment of structural rearrangements is performed, so it is possible that this type of variation contributes to tumorigenesis in the model.

We agree with the reviewer to some degree. In this study, genome analysis was performed using short-read whole-exome sequencing (WES) and structural rearrangements were not assessed. Therefore, we cannot completely exclude the possibility that such alterations contributed to tumorigenesis, and we recognize this issue as a limitation of our study. However, across multiple independently-generated HCCs, we observed highly consistent gene expression patterns and shared activation of DNA demethylation target genes (revised Fig. 1h, i; Fig. 4b, c). Furthermore, we

have now added TET1 knockdown experiments demonstrating that TET1 is required for YAP1–TEAD–induced HCC formation (revised Fig. 3d, e). Taken together, these findings indicate that tumor formation in this model is primarily driven by epigenetic changes mediated by the YAP1–TEAD–TET1 axis, rather than by driver gene mutations (DGMs). Based on these considerations, we have revised our Title, Abstract, Introduction, Results, and Discussion sections as follows:

Title:

YAP1–TEAD–TET1 axis induces hepatocellular carcinoma via DNA demethylation rather than driver gene mutations

Abstract:

1. However, a small fraction of cancers are not positive for these canonical DGMs, leaving the mechanisms underpinning their formation a mystery. We hypothesized that canonical DGM-negative cancers might be driven by activation of the transcriptional coactivator YAP1 that led to the induction of epigenetic changes.
2. Whole-exome sequencing did not identify canonical DGMs in HCCs

Introduction:

1. A subsequent high-depth WGS analysis of 494 hepatocellular carcinoma (HCC) samples revealed the existence of canonical DGM-negative HCCs [5]. (Page 6, Lines 11–12)
2. These findings imply that HCCs can be induced by alternative mechanisms that can drive carcinogenesis independently of genomic canonical driver DNA mutations [10]. (Page 6, Line 18-Page 7, Line 1)

Results:

1. None of the coding mutations identified in HCC1, HCC2, or HCC5-2 corresponded to known canonical DGMs in the databases analyzed. (Page 14, Lines 14-16)
2. Thus, YAP1-TEAD activation led to HCC formation in the absence of canonical DGMs in about 50% of cases. (Page 15, Lines 4–5)

Discussion:

1. Because we used short-read WES for our work, we cannot completely exclude the possibility that non-coding driver mutations or structural rearrangements may have contributed to the observed tumorigenesis. (Page 27, Lines 7-9)

2. None of our six HCCs showed elevated *TERT* expression (Supplementary Table 1), and consistent gene expression and activation of DNA demethylation targets were observed in all of our YAP1–TEAD-induced HCCs. Furthermore, TET1 knockdown suppressed tumorigenesis in our mice. Taken together, these findings indicate that HCC formation in our model is primarily mediated by epigenetic changes driven by a YAP1–TEAD-TET1 axis rather than by changes to the DNA sequence itself, including non-coding mutations or structural rearrangements. (Page 27, Line 15- Page 28, Line 4)

d. The absence of cancer-promoting genetic aberrations in tumor samples is much more difficult to definitively demonstrate than their presence. Methodologies utilized may not be able to detect certain types of mutations (e.g. short-read sequencing cannot adequately assess structural rearrangements). Sampling bias may cause mutations to be missed. The absence of detectable mutations in known driver genes, as limited by methodologies utilized and depth of analysis, is not the same as a lack of driver gene mutations in a sample.

We agree with the reviewer on this matter. The potential limitation of short-read sequencing in detecting structural rearrangements has been addressed above in our response to concern (c). In human tumors, it is common to analyze a very limited portion of the tumor, such as that provided by a needle biopsy specimen or small tissue fragment. In contrast, in our mouse study, we analyzed approximately 20% of the malignant tissue obtained from each tumor. Therefore, we think it unlikely that sampling bias has affected our analyses.

e. References to human tumors lacking driver gene mutations are discussed in the Introduction on page 5, with two main studies cited (References 3-4). Additionally, DGM-negative cancers are described as a "phenomenon often observed in human malignancies", which is not accurate based

on the data provided.

We apologize for this error and recognize that our previous description of references "lacking driver gene mutations" was not accurate. We have revised the text accordingly.

i. Reference 3 does not make a claim that a subset of tumors analyzed lacked driver gene mutations. This interpretation by the authors of the current manuscript appears to come from the oncoplot shown in Figure 1b, which lists mutations in specific genes identified in the patient cohort and shows a group of 38 samples for which none of these mutations were detected. Supplemental Table 1b provides more comprehensive data on all 494 analyzed HCCs. Among the 38 samples for which no mutations were detected in the genes shown in the oncoplot, there are over 1,500 instances of structural variations, chromothripsis, kataegis, altered ploidy, and extrachromosomal DNA that could affect activity of cancer-promoting genes.

We agree with the reviewer that we overinterpreted the oncoplot data. As the reviewer points out, Supplementary Table 1b reports various cancer-associated genomic alterations, including structural variations (SVs). These cases therefore harbor genomic alterations suggestive of genomic instability. However, these alterations have not been necessarily demonstrated to affect cancer-promoting genes. Therefore, the possibility of there being cancers negative for canonical DGMs remains.

ii. In Reference 4, "driver genes" are defined relatively narrowly as known promoters of lung cancer. The authors of the current manuscript highlight the finding that "19% of these tumors in never-smokers had no detectable DGMs". This number is based on data shown in Figure 2 of Reference 4, for which "driver genes" were defined as "known activating mutations or fusions in RTK/RAS/RAF pathway genes". By this same classification, 51.5% of tumors from smokers fall in the same category, and it is well accepted that smoking-associated lung cancer is driven by accumulated mutations. Data presented in Figure S2 of Reference 4 uses a broader definition of "drivers" as "LUAD-related genes", finding alterations in 94% of tumors from never-smokers. This definition is still too narrow to claim absence of driver gene mutations, and other types of mutations (e.g. structural variations) were not comprehensively assessed.

We agree that the previously published definition of "driver genes" was limited to the RTK/RAS/RAF pathway in lung cancer, and therefore this reference was not appropriate for supporting our statement. We have replaced Reference 4 with a study of HCCs developing in Fontan-associated liver disease (FALD) patients. FALD is a congestive liver disease caused by the chronically elevated

central venous pressure that results following the use of the Fontan procedure to treat congenital heart disease. FALD can progress to fibrosis, cirrhosis, and/or HCC under conditions of persistent hemodynamic stress. FALD-associated HCC is an example of HCC characterized by a paucity of canonical DGMs. We have added the following statement to our revised Results:

"In particular, a paucity of canonical DGMs was found in HCCs linked to Fontan-associated liver disease (FALD), which arise after the Fontan procedure is used to treat congenital heart disease [6,7]. In FALD patients, the liver is subjected to persistent hemodynamic stress due to chronically elevated central venous pressure, leading to the development of both intrahepatic cholangiocarcinoma (ICC) and HCCs that are canonical DGM-negative [8,9]." (Page 6, Lines 12-17)

2. Only protein-altering mutations were considered in the analysis depicted in Figure 3. It is unclear why this requirement was implemented when identifying "driver gene mutations". There are many examples of non-coding alterations known to be drivers of tumorigenesis (e.g. those analyzed in Reference 3).

We would like to clarify our rationale for focusing on protein-altering mutations. In our study, somatic mutation analysis was performed using whole-exome sequencing (WES). Compared with whole-genome sequencing (WGS), WES enables greater sequencing depth for a given sequencing capacity and is therefore suitable for sensitive detection of low-frequency mutations. In our analysis, sequencing was performed at a mean depth of over 330×, allowing robust evaluation of protein-altering mutations in known cancer-related genes.

As the reviewer points out, non-coding alterations can contribute to tumorigenesis. However, recent large-scale pan-cancer whole-genome analyses have shown that such alterations are less frequent than protein-coding driver mutations. For example, a comprehensive analysis of 2,658 cancer genomes (Rheinbay et al., *Nature*, 2020, Revised reference # 4) identified more than 1,475 driver mutations in protein-coding regions, whereas substantially fewer were identified in non-coding regions. The latter included 96 in promoters (73 in the TERT promoter), 22 in 5′ untranslated regions (5′UTRs), and 68 in 3′ untranslated regions (3′UTRs). Although non-coding regions were not directly analyzed in our study, we did not observe increased expression of TERT, a representative non-coding driver-associated gene, in the six HCCs analyzed (Supplementary Table 1). Given these considerations, we feel we are justified in focusing on protein-altering mutations in our study. Nevertheless, because we cannot exclude the possibility that non-coding alterations may have occurred, we have added the following sentences to our revised Discussion:

“Because we used short-read WES for our work, we cannot completely exclude the possibility that non-coding driver mutations or structural rearrangements may have contributed to the observed tumorigenesis. However, recent pan-cancer whole-genome analyses have shown that non-coding drivers are rare compared to protein-coding drivers [4,56,57]. In a comprehensive analysis of non-coding somatic drivers in whole genomes of 2,658 cancer cells, it was predicted that more than 1,475 true driver mutations occurred in the protein-coding sequences of cancer-related genes [4]. In contrast, only 96 driver mutations were estimated to have arisen in promoters (73 attributed to TERT), 22 in 5′ untranslated regions (5′UTRs), and 68 in 3′ untranslated regions (3′UTRs). None of our six HCCs showed elevated TERT expression (Supplementary Table 1), and consistent gene expression and activation of DNA demethylation targets were observed in all of our YAP1–TEAD-induced HCCs. Furthermore, TET1 knockdown suppressed tumorigenesis in our mice. Taken together, these findings indicate that HCC formation in our model is primarily mediated by epigenetic changes driven by a YAP1–TEAD-TET1 axis rather than by changes to the DNA sequence itself, including non-coding mutations or structural rearrangements.”（Discussion section, Page 27, Lines 7- Page 28, Lines 4）

3. From the single-cell spatial transcriptomics analysis, four HCC-specific clusters were identified. Clusters 1-3 each expressed HCC markers, yet only Clusters 1 and 3 expressed Yap1 and its downstream targets, according to wording on page 16, lines 264-267. Does this finding suggest non-cell-autonomous effects of the YAP transgene in Cluster 2 cells? Is there an alternative interpretation for this cluster of HCC cells that don’t express Yap1 and its target genes? More discussion is warranted on this topic.

a. In the Discussion section, it is stated that “the same YAP1-TEAD target genes” are expressed across Clusters 1-4, which seems to contradict with the main text.

We apologize for this inconsistency between the main text and the Discussion. In our revised manuscript, we have corrected the description to clarify that YAP–TEAD target gene expression was upregulated across all HCC clusters (Clusters 01–04) compared with normal hepatocyte clusters (Hepatocytes 1–6). Therefore, there is no need to discuss non-cell-autonomous effects in Cluster 02.

4. Figure 7a,e display p-value=0 for gene set correlations. A p-value of zero is not possible. As with other figures in the manuscript “$p < x$” should be used instead of $p = 0$.

We apologize for this obvious error and have revised our statistical significance notation to read “$p < 0.0001$”.

Minor Issues

1. TAZ is not mentioned at all in the manuscript. YAP and TAZ function at the same mechanistic level in the Hippo signaling pathway. They are often treated as interchangeable/redundant mediators of Hippo signaling. While this characterization is an oversimplification, it would be beneficial to include some discussion of TAZ, at least in the Introduction and/or Discussion section. Is it expected that transgenic expression of active TAZ would produce a similar model of HCC?

We thank the reviewer for this helpful suggestion. We have added a description of TAZ in the Introduction and clarified the rationale for focusing on YAP1 in this study, as follows:

“While TAZ (WWTR1) is a closely related paralog of YAP1 and also a Hippo pathway effector, previous studies have indicated that liver tumor phenotypes are mostly dependent on YAP1 and only partially dependent on TAZ [20,27]. These observations prompted us to focus on YAP1 in the present study.”（Introduction section, Page 8, Lines 8-12）

2. In Table 1, several constructs are shown that are not described in the manuscript. In particular, the 5SA/WW1,2* construct (which according to Reference 37 is defective in binding transcription factors, such as p73) drives robust HCC formation in all mice analyzed. What is the interpretation of this result? Any of the constructs shown in Table 1 should be discussed in the text of the manuscript.

As the reviewer points out, the YAP1 constructs shown in Table 1 were not sufficiently described in the original text. In our revised manuscript, we have added a description of this mutant, as follows:

“The YAP1(5SA/WW1,2) mutant, which is defective only in WW domain–mediated protein-protein interactions (including binding to transcription factors such as p73), retained the ability to induce HCC in male mice.”（Page 11, Lines 5-8）

3. Page 10, lines 151-153: The authors discuss significantly upregulated and downregulated genes identified by RNA-Seq. It would be valuable to perform gene set enrichment analysis (GSEA) to determine whether YAP-TEAD target gene sets are significantly enriched in tumors versus normal liver samples. Several such gene sets have been described (e.g. in PMID 38473214).

We thank the reviewer for this valuable suggestion. We have performed gene set enrichment analysis (GSEA) using the YAP/TAZ target gene set identified in the study cited by the reviewer (PMID: 38473214). The results showed that the YAP/TAZ gene signature was significantly enriched in HCC samples compared with non-tumor tissues (NES = 1.8, FDR = 0.0001). These findings support our conclusion that YAP1 signaling is activated in our HCC model. These GSEA results have been added as revised Supplementary Fig. 2c.

4. Did the authors analyze RNA-Seq data for altered splicing or RNA editing in HCCs versus normal liver samples? This data could be informative.

We agree with the reviewer that altered splicing and RNA editing may represent important mechanisms in HCC progression. However, this particular study focused on genomic DNA alterations and DNA methylation changes. Therefore, we feel proper analyses of splicing and RNA editing are beyond the scope of the present study, but they will be considered in future work.

5. Figures 4b and 5e show data from 11 HCCs and 10 non-tumor samples, while Figure 2e-g only shows the 6 HCCs and 5 non-tumor samples that were the focus of most of the manuscript. It is unclear which additional samples were utilized for the analyses in Figures 4-5.

We apologize for our lack of clarity. In our revised manuscript, we have addressed this point by adding the following statement to the revised Results section:

"Among members of the Tet gene family, only Tet1 was significantly upregulated at the mRNA level compared to non-tumor controls in a total of 11 HCCs (HCC1, 2, 3, 4, 5-1, 5-2 and an additional 5 YAP1-TEAD HCCs that were separately induced) (Fig. 3b)." (Page 15, Line 16- Page 16, Lines 1)

6. Page 14, Line 222: It is unclear what is meant by a fold-change of ≤1/1.5.

We agree that the expression "fold-change ≤1/1.5" is unclear. In our original analysis, a fold-change threshold of 1.5 was used to evaluate changes to DNA methylation. In our revised manuscript, we have reanalyzed the data using a more widely adopted and statistically robust method, namely a generalized linear model (GLM) with a binomial error structure. In our revised Methods section, we have removed "fold-change ≤1/1.5" and replaced it with the details of our GLM-based analysis, as follows:

"Differential methylation analysis and annotation. CpG report files generated by Bismark as above were used to identify "Differentially Methylated Cytosines" (DMCs) between two sample groups (e.g., Non-Tumor vs. Tumor). First, all CpG sites with a minimum read coverage of 10x in at least two samples were consolidated into a union site list for subsequent statistical testing. Differential methylation at each CpG site was assessed using a Generalized Linear Model (GLM) with a binomial error structure. This model was applied to the counts of methylated and unmethylated reads, with the Non-Tumor or HCC status set as the explanatory variable. The resulting P-values were adjusted using the Benjamini-Hochberg method to control the False Discovery Rate (FDR). A CpG site was classified as a DMC if it met two strict criteria: (1) a q-value less than 0.01, and (2) an absolute methylation difference between the groups of ≧20%. The overall distribution of the q-values and methylation differences across the genome was visualized in a Manhattan-style plot. DMCs were mapped to corresponding gene symbols using org.Mm.eg.db." (Page 41, Lines 3-16)

7. Page 16, Lines 260-262: Do any of the gene expression signatures identified in HCC clusters 1-4 overlap with findings from the bulk sequencing analysis?

We thank the reviewer for this important question. Overlap was observed between the bulk RNA-seq analysis and the Visium HD spatial transcriptomics analysis. For example, the signature related to "movement of cell or subcellular component" that was identified by bulk RNA-seq corresponds to the "positive regulation of early endosome to late endosome transport" signature identified by Visium HD analysis (revised Fig. 5e and Supplementary Fig. 2a). On the other hand, these results are not completely identical, which is likely due to the differences in analytical resolution. The bulk RNA-seq method captured averaged gene expression levels across heterogeneous cell populations, whereas the Visium HD analysis evaluated gene expression within individual HCC clusters.

8. In Figure 1c-d, it is unclear whether an anti-YAP antibody (as used in Figure 1c) or an anti-Myc antibody was used for immunofluorescence. This point is important, as it determines whether total YAP or only transgenic YAP is detected.

We apologize for our lack of clarity. In original Figures 1c and 1d, immunofluorescence was performed using an anti-Myc antibody to detect exogenous Myc-YAP1(2SA). In the revised manuscript, we have clarified this point by adding "using anti-Myc tag antibody" to the revised Fig. 1c and e legend.

9. In Figure 6m, it would improve clarity if a border could be drawn around the portion of the tissue section that is histologically HCC.

We thank the reviewer for this helpful suggestion. We have added an annotated image with a boundary outlining the HCC region in revised Supplementary Fig. 6b.

10. Page 18, lines 301-303: The authors suggest that the gene expression signature may be applicable to additional cancer types. It would be simple to do similar assessments as shown in Figure 7 on additional TCGA datasets.

We thank the reviewer for this valuable comment but have chosen to focus on the mechanism of HCC formation for this manuscript. We have removed the original mesothelioma analysis from the text and have replaced it with an analysis of Fontan-associated liver disease (FALD)-related HCC, which is characterized by a dearth of canonical DGMs (revised Fig. 7a–c). Evaluation of other cancer types by examining additional TCGA datasets will be considered in future work.

11. Page 19, Lines 307-308: The statement "YAP1 was introduced into single hepatocytes, followed by mosaic analysis" is strangely phrased. Something like "YAP1 was introduced mosaically into hepatocytes" would be more clear.

We apologize for this awkwardness and have revised the phrasing to read: "activated YAP1 was introduced into hepatocytes in mosaic fashion". (Page 26, Lines 4-5)

Reviewer #2 (Remarks to the Author):

In this manuscript, Misaki Kosaka et.al reported a HCC model induced by sustained YAP1 activation was successfully established and found that YAP1–TEAD–TET1 axis as a possible cause of DGM-negative HCC formation,and uncoverd a potential molecular mechanism that may be applicable to common human cancers.

My main criticism of the manuscript relates to the fact that YAP1–TEAD–TET1 axis drives liver cancer growth have to be reported (Wu, B.K et al Nat Genet, 2022). And the data seem like to be preliminary and need to be further analyzed. It may be necessary to address whether a novel mechanism discovered by the authors, although the other novel targeted genes were identified except YAP1–TEAD–TET1 axis. And how and why other novel targeted genes regulating HCC tumor growth showed be ananlyzed and discussed in the manuscript. Overall, the manuscript does not meet the high quality of the journal, I recommend the manuscript currently could not be published.

We thank the reviewer for these comments and address them as follows:

A. Response to the concern regarding the novelty of the YAP1–TEAD–TET1 axis

1. One of the key novelties of our study is that we focused on the paradox of the existence of HCCs negative for canonical DGMs and the lack of DGMs detected in the Hippo–YAP1 research field. We have demonstrated that a YAP1–TEAD-TET1 axis can induce HCCs without canonical DGMs (revised Fig. 1 and Fig. 2).

2. By comparing the results of Wu *et al*. with our findings, we identified novel target genes of the YAP1–TEAD-TET1 axis that were related to disease stage**.** Whereas Wu *et al.* identified immune-related target genes in the initiation stage of HCC formation, we identified target genes, including cytoskeletal regulation-related and multiple oncogenes, in the progression stage of HCC formation (revised Fig. 4).

3. We have performed a single-cell spatial transcriptomics analysis and demonstrated that clonally expanded hepatocytes ultimately form an HCC composed of heterogeneous cell populations (revised Fig. 5).

4. Using CellChat analysis, we have identified intercellular interactions between heterogeneous HCC cells and normal non-parenchymal cells, including liver sinusoidal endothelial cells (LSECs) and hepatic stellate cells (revised Fig. 6).

5. We have analyzed single-cell RNA-seq data from FALD liver tissues, which cause HCCs characterized by a paucity of canonical DMGs. FALD is a congestive liver disease that develops

after the Fontan procedure is used to treat congenital heart disease and is characterized by chronically elevated central venous pressure. Our analysis revealed activation of the YAP1–TEAD–TET1 signaling pathway and upregulation of its target genes in FALD hepatocytes, supporting the relevance of our findings to human disease (revised Fig. 7).

Based on these points, we believe that our study definitely provides novel insights.

B. Response to the concern that the data are preliminary and require further analysis

To address this comment, we have performed the following additional analyses now included in the revised manuscript, as follows:

1. We have added *in vivo* Tet1 knockdown experiments which have demonstrated that TET1 plays an essential role in YAP1–TEAD–induced HCC formation (revised Fig. 3).

2. We have expanded our RRBS experiments from covering only promoter regions to a genome-wide analysis. As a result, the number of YAP1–TEAD–TET1 target genes increased from 26 to 1,125, leading to the identification of 55 oncogenes that can explain the molecular basis of HCC development (revised Fig. 4).

3. We have performed CellChat analysis and identified intercellular interactions among HCC clusters and normal non-parenchymal cells (e.g. LSECs and hepatic stellate cells). We found that Cluster 01 cells, which show high Tet1 expression, play central roles in HCC formation (revised Fig. 6).

4. We have analyzed single-cell RNA-seq data from human FALD liver tissues and have identified activation of the YAP1–TEAD–TET1 signaling pathway (revised Fig. 7).

Based on these additions, we believe that our data are substantive and far from preliminary.

C. Response to the concern regarding the functional relevance of target genes

In our revised manuscript, we have analyzed 1,125 newly identified YAP1–TEAD–TET1 target genes to clarify their roles in tumorigenesis. Firstly, by comparing these target genes with known driver gene databases, we identified 55 oncogenes that can explain HCC development (Fig. 4g). These results suggest that the YAP1–TEAD–TET1 axis induces HCC through activation of multiple oncogenes via DNA demethylation. Secondly, to investigate the roles of these target genes in the

formation of the tumor microenvironment (TME), we performed CellChat analysis and found that SPP1 and APP, which are encoded by target genes upregulated in HCC cells, were secreted and interacted with receptors expressed on non-parenchymal cells such as liver sinusoidal endothelial cells (LSECs) and hepatic stellate cells (Fig. 6, Supplementary Table 10). These findings suggest that the YAP1–TEAD–TET1 axis may activate genes whose protein products contribute to HCC TME formation. We have added the following descriptions to the revised manuscript:

"We identified multiple known oncogenes across all groups (Fig. 4h; Supplementary Fig. 4). Specifically, the Day 10 group included *Ccnd1* and *Src*; the shared group (Day 10 & 4M) included *Myc*, *Bcl2*, and *Pdgfb*; and the 4M group included *Ctnnb1*, *Stat3*, *Braf*, and *Hras* (Supplementary Table 14; Supplementary Fig. 5). Taken together, these data indicate that demethylation-mediated upregulation of multiple oncogenes may be involved in the initiation and progression of YAP1-TEAD-induced HCCs."（Page 18, line 10-16）

"Notably, Cluster 01 also expressed multiple oncogenes, including SPP1 and APP. These genes are DNA demethylation targets of the YAP1–TEAD–TET1 axis and have previously been implicated as contributors to TME formation [49,52,53,61,62]." (Page 30, Lines 3-6)

Major points:

1. In the manuscript, gender difference was observed in that female mice transfected with the same plasmids developed liver cancer less frequently. The potential roles of ER and AR in the hepatocytes for HCC progession need to be considered in future.

The reviewer points out an important sex difference in our model. Sex differences in HCC incidence in both mice and humans have been reported in multiple studies (e.g., Naugler et al., *Science*, 2007; Kanda et al., *J Hepatocell Carcinoma*, 2015), and have been attributed, at least in part, to differences in transcriptional regulation mediated by estrogen receptor (ER) and androgen receptor (AR) signaling. Accordingly, we have added the following statement to the revised Results section, along with the relevant references:

"As expected, we observed a sex difference in that female mice transfected with the YAP1(2SA) plasmid developed HCC less frequently than males. This sex difference is in line with the well-known male predominance of HCC in both mice and humans [40,41]." (Page 11, Lines 12-14)

2. The potential clinical features in human liver cancer related to DGM-negative HCCs should be

analyzed and discussed.

We thank the reviewer for this important comment. As described above, in our revised manuscript, we present a new analysis of Fontan-associated liver disease (FALD)-associated HCC as an example of HCC characterized by very few DGMs (Fig. 7a–c). The clinical features of FALD-associated HCC include persistent hemodynamic stress, which may constitute a persistent mechanical stress that induces sustained YAP1 activation. FALD-associated HCC is also known to develop in patients at a relatively young age, with a median age of onset of 30–34 years. This relatively short period available for the accumulation of somatic DGMs may be one factor contributing to their scarcity in these HCCs. We have commented on the relationship between DGM-negative and DGM-positive HCCs in our revised Discussion (see below) and have revised the model shown in Fig. 7h.

“ In FALD patients, hemodynamic stress is considered to play a critical role in initiating carcinogenesis [6,63,64]. Recently, Hu et al. reported that hepatocytes near the hepatic central vein underwent metabolic reprogramming as an initial response to hepatic congestion [55]. Although the mechanical stresses caused by hepatic congestion are known to activate the YAP1 pathway [65,66], the potential involvement of YAP1 in driving FALD-associated HCC has remained unresolved. In our study, we provide novel data supporting the idea that epigenetic changes induced by activation of the YAP1-TEAD-TET1 axis constitute the molecular basis of FALD-associated HCC. Indeed, our analysis of human FALD scRNA-seq data revealed specific upregulation of TET1 and Cluster 01 target genes in FALD hepatocytes (Fig. 7b, c). Notably, FALD patients develop HCC at a younger age (median 30–34 years) than those with classical HCC [6,9], leaving insufficient time for the accumulation of somatic driver mutations. This early onset may be one factor explaining the paucity of canonical DGMs in FALD-related HCCs [8,9].” (Discussion, Page 30, Line 10- Page 31, Line 4)

Reviewer #3 (Remarks to the Author):

In this study, Kosaka et. al. aimed to understand how hepatocellular carcinomas (HCC) lacking driver gene mutations (DGMs) may arise. YAP1 activation is frequently observed in HCC patients with poor prognosis yet is typically not mutated in these cancers. The authors hypothesize a 2-step process in which fibrosis/inflammation induces YAP1 activation which subsequently activates pro-survival, oncogenic pathways with the transcription factor TEAD through DNA demethylation of YAP1-TEAD target genes. Using CCl4 treatment in C57BL/6J mice, liver fibrosis was induced and coincided with expression of YAP1 over a period of 15 weeks. In a separate model, YAP1(2SA) sustained expression induced HCC, both DMG(+) and DMG(-), in male mice over a period of 4 months while WT YAP1 did not. Profiling of the resultant HCC transcriptomes revealed conserved gene expression profiles among the HCC tumors and demonstrated upregulation of epigenetic modifiers, including a known target of YAP1-TEAD, the DNA demethylase Tet1. RRBS DNA methylation analysis revealed both hypermethylation and hypomethylation of promoter CpG sites, with demethylation correlating with upregulation of several target genes. Spatial transcriptomics of a normal and YAP1-TEAD induced HCC revealed cell clusters with unique gene expression profiles, with Cluster01 expressing YAP-TEAD target genes, Tet1, and the identified DNA demethylation target genes. Indeed, these markers demonstrate poor prognosis in liver cancer patients.

We thank the reviewer for this accurate summary of our study.

While the end conclusions of this work agree with published literature regarding YAP1 activation in HCC, the observations made are correlative rather than causative. Indeed, the 2-step hypothesis is uncoupled experimentally and does not support the hypothesis that environmentally induced YAP1 activation promotes the development of DGM- HCC.

As the reviewer correctly points out, our original data supporting Step 1 of the proposed two-step hypothesis—sustained activation of YAP1 induced by environmental factors—were insufficient. Therefore, we have removed the fibrosis data (Fig. 1b) and the corresponding description from our revised manuscript. Instead, we have added examples of HCC characterized by persistent hemodynamic stress, which is considered to induce sustained YAP1 activation (Fig. 7a-c). Fontan-associated liver disease (FALD) is a congestive liver disease caused by the chronically elevated central venous pressure that follows the use of the Fontan procedure to treat congenital heart disease. FALD is known to progress to fibrosis, cirrhosis, and/or HCC under conditions of persistent hemodynamic stress. Notably, FALD-associated HCC has been reported to exhibit very few

canonical DGMs.

Furthermore, to move beyond correlative observations and provide evidence supporting causality, we have added new TET1 knockdown experiments to our revised Results. These experiments have demonstrated that TET1 is required for YAP–TEAD–dependent tumor formation (Fig. 3). Together, these results support a model in which the YAP1–TEAD–TET1 axis contributes to HCC development through DNA demethylation.

1. In Figure 1, while CCl4 induced fibrosis, it is unclear from the images presented if YAP1 is indeed adjacent to the fibrotic tissue. The authors state at Line 110 "robust upregulation and clear nuclear accumulation of YAP1", yet overexpression of YAP1 is observed in the cytoplasm as well as the nucleus. Given that YAP1 cellular localization is dependent on phosphorylation, the overexpression of YAP1 in the cytoplasm should also be considered.

We thank the reviewer for this insightful comment. However, as described above, the corresponding data (Fig. 1b) have been removed from the revised manuscript. Therefore, this point is no longer relevant.

2. No definition for the consequence of the YAP1 (S2A) mutation is provided other than at Line 120 – "constitutive YAP1 activation". Why do these mutations cause constitutive YAP1 activation? There is no reference provided for this statement. Published literature provides an explanation regarding phosphorylation of these sites, and that mutation of Serine127 prevents sequestering of YAP1 to the cytoplasm. Indeed, this highlights a disconnect between the model for Step #1 versus Step #2 as cytoplasmic YAP1 is observed in the model for Step #1 while the model in step #2 enforces YAP1 localization to the nucleus.

We apologize that our original explanation and references regarding the functional consequences of the YAP1(2SA) mutant were insufficient. We have added the following description to the revised Results section:

"To create active YAP1, we took advantage of the YAP1(2SA) mutant (Fig. 1b) in which phosphorylation-dependent cytoplasmic retention (S127) and proteasomal degradation (S397) are impaired, resulting in constitutive nuclear localization and transcriptional activation [38,39]." （Page 10, Lines 8-11）

In addition, as noted above, the data corresponding to Step 1 were insufficient, and therefore the

relevant figure panel (Fig. 1b) has been removed from the revised manuscript.

3. The authors hypothesized that YAP1 activation induces DGM- HCCs. While DGM- HCCs are observed in the model, so are DGM+ HCCs. The conclusion regarding this data should be modified as it appears YAP1 (S2A) induces HCC, both with and without DGMs.

As the reviewer correctly points out, both DGM-negative and DGM-positive HCCs were observed in this model. Our main conclusion is that, regardless of DGM status, the primary mechanism driving tumorigenesis is epigenetic reprogramming mediated by the YAP1-TEAD-TET1 axis. To clarify this causal relationship, we have added TET1 knockdown experiments, which have demonstrated that TET1 is required for YAP-TEAD-dependent HCC formation. In addition, we now include a model illustrating the relationship between DGM-negative and DGM-positive HCCs (Fig. 7h).

4. Line 135, "AFP, CK19, Ki67 or aSMA showed these liver cancers were HCCs". No references are provided for what these markers indicate, and it is not apparent to a more general audience that may not be experts in liver cancer.

We apologize that the references for these markers were not provided and that their biological significance was not adequately explained. We have added descriptions of the biological characteristics of each marker along with the relevant references, as follows:

"Tissue staining with H&E or Sirius Red, or with antibodies to detect AFP (HCC marker), Ki67 (proliferation marker), CK19 (cholangiocyte marker), or αSMA (activated hepatic stellate cells), showed that these liver cancers were HCCs (Fig. 1e, right) [42]." （Page 11, Line 17- Page 12, Line 2）

5. Several of the epigenetic modifiers profiled as upregulated by gene expression are cell cycle regulated genes (such as the maintenance methyltransferase Dnmt1). As shown in Figure1d, HCC has a higher proliferative index (Ki67 staining). This is validated in the spatial transcriptomics as Cluster03 demonstrates the highest expression of cell proliferation genes (Fig 6h) coinciding with expression of DNA methylation modifiers (Figure 6j). Normalization of gene expression by proliferative index should be considered to determine if this is indeed "overexpression" in HCC or a consequence of increased cell proliferation relative to the controls.

We thank the reviewer for this important comment. As shown in Fig. 6, the Visium HD data are already normalized, and we consider that additional normalization by a proliferative index is not statistically appropriate. In Cluster 03, both cell proliferation–related genes and DNA methylation–related genes, including *Tet3*, *Uhrf1*, *Dnmt1*, and *Mbd2*, are upregulated. However, it remains unclear whether the increased expression of these genes reflects intrinsic upregulation or is a consequence of enhanced cell proliferation.

6. Line 220 – "Our analysis focused on 51,414 promoter regions (out of 122,460 total)". This statement is confusing as that is more promoters than are observed in the mouse genome. Figure 5 indicates this is CpG sites within promoters. This point needs clarification.

We apologize for this confusion. The numbers referred to CpG sites within the promoter regions rather than the number of promoters. In the revised manuscript, we have clarified the distinction between CpG sites and gene numbers (revised Fig. 4).

7. Previous work conducted on the YAP1-TEAD-TET1 axis in HCC demonstrated demethylation at enhancers for modulating gene expression rather than promoters (PMID: 35835915); however, the analysis for this work was focused on promoters. If RRBS coverage is available, enhancers should be considered in this analysis as well.

We thank the reviewer for this valuable suggestion. In our original analysis, RRBS was limited to promoter regions. In the revised manuscript, we have expanded the analysis to include broader genomic regions, including enhancers (revised Fig. 4a). As a result, the number of DNA demethylation target genes increased from 26 to 1,125, and we identified 55 oncogenes as potential targets (revised Fig. 4b–d).

8. "Control" livers are not discussed until line 243 yet is used throughout the gene expression analysis with no definition provided for the source of these controls. "Control" livers need to be clarified in the text.

We apologize for our lack of clarity. In the revised manuscript, we have defined "control liver" at its first occurrence in the Results section as follows:

"Livers from three normal male mice were used as controls."（Page 12, Lines 9-10）

Point-by-Point Rebuttal to the Reviewers for Manuscript # COMMSBIO-25-7657A-Z

Reviewer #1 (Remarks to the Author):

In this revised manuscript, the authors have adequately addressed nearly all of the issues raised in my review of the initial submission. In some cases, however; some slight additional modifications should be made, as detailed below. Additionally, the authors have added a substantial amount of new data and discussion to the revised manuscript. Based on this new information, one major issue and 5 minor issues should be addressed prior to acceptance for publication.

We sincerely thank the reviewer for carefully evaluating our manuscript. In this revision, we have addressed the major issues remaining from the initial submission, as well as the newly raised major and minor issues.

Remaining Major Issues (Initial Submission)

Reviewer #1 (R1): 1. Claims of tumors lacking driver gene mutations" need to be removed, revised, or significantly qualified. This is a major theme throughout the manuscript and even included in the title but is, in my opinion, unjustified. Several reasons are provided below.

a.The mouse model utilized to generate HCCs is driven by expression of a transgenic YAP construct that has been engineered with serine-to-alanine mutations that render it unaffected by phosphorylation-based regulatory mechanisms that would otherwise decrease its signaling activity. At a fundamental level, these tumors are essentially induced by driver gene mutations" in the YAP transgene, which is expressed from a promoter with very high activity in the liver (Alb). As the authors state, mutations in YAP are very rare in human cancer, though activation of the pathway is fairly common. While the transgene is meant to mimic YAP-TEAD activation that can occur downstream of fibrosis or other disease states, it still represents a mutant form of YAP that is essential to the model. Notably, a wild-type YAP transgene delivered the same way did not produce tumors in any of the 22 mice listed in Table 1.

Authors (A): We thank the reviewer for this important comment. In this study, we used a mutant form of the oncogene YAP1, YAP1(2SA), to induce hepatocellular carcinoma (HCC) in mice. The rationale for using this system was to induce HCC within a short period (4 months). In contrast, sustained activation of endogenous YAP1–TEAD by mechanical stress (without genetic alteration) requires a

prolonged period to induce HCC. Indeed, as the reviewer points out, expression of wild-type (WT) YAP1 did not induce HCC even after 12 months. Therefore, we consider that this experimental system using YAP1(2SA) represents an appropriate model to mimic the results of sustained YAP1–TEAD activation that would otherwise require a long time to induce malignancy.

R1: I agree that the model is appropriate and justified. Transformation and tumor growth is, however, still induced by expression of a driver gene harboring an activating mutation. The authors' modifications to the text are appreciated and have sufficiently clarified and qualified conclusions in almost all sections. There are some remaining instances where DGM-free" should be changed to Cnonical DGM-free", as listed below.

Page 13, Line 11
Page 26, Lines 3-4, 9, 11, 17
Page 30, Line 8

A: As suggested by the reviewer, we have changed "DGM-negative" to "canonical DGM-negative" throughout the relevant parts of the revised manuscript.

R1: b.The hydrodynamic tail vein injection method used to deliver the YAP transgene results in stable incorporation of recombinant DNA into the genome of hepatocytes. Transgene integration can cause insertional mutagenesis, depending on integration sites. As integration sites were not assessed, it is possible that insertional mutagenesis could contribute to tumor formation. There is precedent for mouse tumors, including HCC, being driven by transgene-induced insertional mutation (e.g. PMID 17656716).

A: We agree with the reviewer on this important point. Genes introduced by hydrodynamic tail vein injection (HTVi) can be integrated into the genome, and insertional mutagenesis may occur depending on the integration sites. However, when WT YAP was introduced in this manner, no tumor formation was observed at 6 months (0/10 mice), and no tumors were detected even at 12 months (0/3 mice) (revised Fig. 1d). In contrast, when YAP1(2SA) was introduced, tumor formation was observed in all mice at 6 months (3/3 mice). Based on these results, we consider that tumor formation in this model is unlikely to be driven by insertional mutagenesis.

R1: The delivery method depends on plasmid integration for maintained expression of the transgenic

cargo. A lack of tumor formation with WT YAP constructs in 10 mice does not exclude the possibility that transgene insertions in YAP1(2SA)-injected mice produce oncogenic effects that cooperate with the transgene, which is clearly the primary driver of tumorigenesis. I'm not implying that this is likely to be the case, but it's a possibility that cannot be excluded. Some mention of the nature of the HTVi model (i.e. genomic integration of injected plasmids) should be included somewhere in the manuscript.

A: We agree with the reviewer that plasmids delivered by HTVi can integrate into the genome. However, as noted in our previous response, YAP1(WT) delivery did not induce HCC. Therefore, we consider that insertional effects were not the primary driver of HCC formation in this model. We have added the following sentence to the Discussion section.

"We also cannot exclude the possibility that the HTVi method used in our model might have induced plasmid integration into the genome. However, such integration-associated effects were unlikely to be the primary tumorigenic drivers because HTVi delivery of YAP1(WT) did not induce HCC (Fig. 1d)." (Page 29, Lines 3-7)

R1: 2.Only protein-altering mutations were considered in the analysis depicted in Figure 3. It is unclear why this requirement was implemented when identifying driver gene mutations". There are many examples of non-coding alterations known to be drivers of tumorigenesis (e.g. those analyzed in Reference 3).

A: We would like to clarify our rationale for focusing on protein-altering mutations. In our study, somatic mutation analysis was performed using whole-exome sequencing (WES). Compared with whole- genome sequencing (WGS), WES enables greater sequencing depth for a given sequencing capacity and is therefore suitable for sensitive detection of low-frequency mutations. In our analysis, sequencing was performed at a mean depth of over 330×, allowing robust evaluation of protein-altering mutations in known cancer-related genes. As the reviewer points out, non-coding alterations can contribute to tumorigenesis. However, recent large-scale pan-cancer whole-genome analyses have shown that such alterations are less frequent than protein-coding driver mutations. For example, a comprehensive analysis of 2,658 cancer genomes (Rheinbay et al., Nature, 2020, Revised reference # 4) identified more than 1,475 driver mutations in protein-coding regions, whereas substantially fewer were identified in non-coding regions. The latter included 96 in promoters (73 in

the TERT promoter), 22 in 5′ untranslated regions (5′UTRs), and 68 in 3′ untranslated regions (3′UTRs). Although non-coding regions were not directly analyzed in our study, we did not observe increased expression of TERT, a representative non- coding driver-associated gene, in the six HCCs analyzed (Supplementary Table 1). Given these considerations, we feel we are justified in focusing on protein-altering mutations in our study. Nevertheless, because we cannot exclude the possibility that non-coding alterations may have occurred, we have added the following sentences to our revised Discussion:

"We used short-read WES for our work, we cannot completely exclude the possibility that non-coding driver mutations or structural rearrangements may have contributed to the observed tumorigenesis. However, recent pan-cancer whole-genome analyses have shown that non-coding drivers are rare compared to protein-coding drivers 4,56,57. In a comprehensive analysis of non-coding somatic drivers in whole genomes of 2,658 cancer cells, it was predicted that more than 1,475 true driver mutations occurred in the protein-coding sequences of cancer-related genes 4. In contrast, only 96 driver mutations were estimated to have arisen in promoters (73 attributed to TERT), 22 in 5′ untranslated regions (5′UTRs), and 68 in 3′ untranslated regions (3′UTRs). None of our six HCCs showed elevated TERT expression (Supplementary Table 1), and consistent gene expression and activation of DNA demethylation targets were observed in all of our YAP1–TEAD-induced HCCs. Furthermore, TET1 knockdown suppressed tumorigenesis in our mice. Taken together, these findings indicate that HCC formation in our model is primarily mediated by epigenetic changes driven by a YAP1–TEAD-TET1 axis rather than by changes to the DNA sequence itself, including non-coding mutations or structural rearrangements." (Discussion section, Page 27, Lines 7- Page 28, Lines 4)

R1: The authors' point is well taken. However, WES data typically includes coverage of non-coding exons (i.e. UTRs). If that data is available, it should be included in the mutation analysis.

A: We performed an additional analysis of non-coding somatic variants in WES-captured non-coding exonic regions, including UTRs, based on a report by Rheinbay *et al*. (*Nature*, 2020). These authors performed two analyses: one to identify non-coding driver candidate elements from whole-genome data (Figure 1b and Supplementary Table 5), and another to identify non-coding driver candidate elements restricted to regions surrounding cancer-associated genes (Figures 2b and 4d, and Supplementary Table 11). The former analysis identified seven significant non-coding exonic driver candidate elements (*TOB1* 3′UTR, *ALB* 3′UTR, *NFKBIZ* 3′UTR, *WDR74* 5′UTR, *PTDSS1* 5′UTR, *DTL* 5′UTR, and *MTG2* 5′UTR), whereas the latter analysis identified two significant non-coding

exonic driver candidate elements (*TP53* 5′ region and *FOXA1* 3′UTR). We therefore compared the non-coding exonic somatic variants detected in our WES data that had a tumor allele frequency (TAF) > 0.03 with these nine non-coding exonic driver candidate elements. We detected no corresponding variants in any of our six HCCs. We have added these data as revised Supplementary Figure 8, and have described this analysis in the revised Discussion and Methods sections.

"None of our six HCCs showed elevated *TERT* expression (Supplementary Table 1), and no somatic mutations corresponding to reported non-coding driver candidates [4] were detected among the WES-captured non-coding exonic regions, including UTRs (Supplementary Fig. 8)." (Discussion, Page 28, Line 18- Page 29, Line 3)

R1: 3.From the single-cell spatial transcriptomics analysis, four HCC-specific clusters were identified. Clusters 1-3 each expressed HCC markers, yet only Clusters 1 and 3 expressed Yap1 and its downstream targets, according to wording on page 16, lines 264-267. Does this finding suggest non-cell-autonomous effects of the YAP transgene in Cluster 2 cells? Is there an alternative interpretation for this cluster of HCC cells that don't express Yap1 and its target genes? More discussion is warranted on this topic.

a.In the Discussion section, it is stated that "the same YAP1-TEAD target genes" are expressed across Clusters 1-4, which seems to contradict with the main text.

A: We apologize for this inconsistency between the main text and the Discussion. In our revised manuscript, we have corrected the description to clarify that YAP–TEAD target gene expression was upregulated across all HCC clusters (Clusters 01) compared with normal hepatocyte clusters (Hepatocytes 1). Therefore, there is no need to discuss non-cell-autonomous effects in Cluster 02.

R1: I was initially confused, because Figure 5f (previously 6i) shows very low Yap1 expression in Cluster 02 and Cluster 04, which I interpreted as a lack of transgene expression. I realize now that the transgene encodes human YAP1 (as indicated by capitalization in the manuscript and designation in the cited reference), which would generally not map to the mouse genome based on the degree of exact sequence homology. However, given that detected Yap1 expression is lower in Cluster 02 than in the hepatocyte control, the statement on Page 20, Line 9 regarding Yap1 expression is inaccurate. This statement should be revised to reflect the experimental data

A: Following the reviewer's suggestion, we have revised the relevant statement as follows.

"All four clusters expressed *Tead* family genes and YAP–TEAD target genes, whereas elevated endogenous mouse Yap1 expression was observed in Clusters 01, 03, and 04, but not in Cluster 02 (Fig. 5f)." (Page 21, Lines 10-12)

New Major Issue

R1: 1.In the Introduction, References 8 and 9 are used to support the statement "In FALD patients, the liver is subjected to persistent hemodynamic stress due to chronically elevated central venous pressure, leading to the development of both intrahepatic cholangiocarcinoma (ICC) and HCCs that are canonical DGM-negative". This statement is not supported by the references. Reference 8 describes molecular analysis of 5 FALD-associated liver tumors. In all 5 tumors, mutations altering canonical cancer driver genes are reported, including variants in MAP2K2, NRAS, GNAS, MSH3, CTNNB1, NOTCH1, and others. In many cases, the same genes are recurrently mutated across the small cohort. The basis for the authors' statement regarding a lack of canonical DGMs is unclear. Reference 9 is a Review article that mentions genetic/molecular profiling of FALD-associated liver cancer only by referencing Reference 8. Another reference (PMID 41587344, not cited here) performed WGS and transcriptome profiling on liver tumors from 10 FALD patients, finding rampant mutation and alteration of cancer driver genes.

A: We sincerely thank the reviewer for this important comment and for bringing this issue to our attention. Francalanci *et al.* (Reference 8) stated that "No variants in either the *TERT* promoter or *TP53* were identified in the HCCs as well as how no *TP53*, *CDKN2A/B*, *KRAS*, and *IDH1* variations and no *FGFR2* fusion were present in the ICC (Intrahepatic cholangiocarcinoma)." In addition, as shown in detail in Table 4, the variants detected in patient P3 (*FGFR3*, *LRP1B*, *CTNNB1*, *KDM6A*, *GNAS*, and *RANBP2*), and those detected in patient P2 (*FGFR3*, *FGFR2*, *GNAS*, *ATRX*, and *PALB2*), were also present in the non-lesional liver at similar variant allele frequencies (VAFs). The only tumor-specific variant detected in patient P2 was an *MSH3* variant, but its VAF was low (6%). These findings suggest that the detected variants were not able to function as effective tumor-specific driver mutations. Regarding the study by Yamazoe *et al.* (PMID: 41587344; *Hepatology*, 2026), the authors concluded that "The top-ranked altered SNVs in the ICGC (International Cancer Genome Consortium) HCC cohorts were in *CTNNB1*, *TERT* promoter, and *TP53*; these were not seen in the FALD HCCs or non-Fontan CHS (congenital heart surgery other than the Fontan procedure) HCCs." Figure 3A in this paper also shows that mutations in the other 33 standard driver genes examined

were not detected in FALD-HCCs. These findings suggest that FALD-associated HCCs harbor canonical DGMs at lower frequencies than other types of HCCs. We now cited the study by Yamazoe *et al.* in our revised manuscript as reference #10. Also as suggested by the reviewer, we have revised the relevant statements to be more accurate, as follows.

"In FALD patients, the liver is subjected to persistent hemodynamic stress due to chronically elevated central venous pressure, leading to the development of both HCCs and intrahepatic cholangiocarcinoma (ICC) [6-9]. Recent studies have reported some genomic mutations in FALD-associated HCCs [8,10]. However, canonical HCC DGMs, including *CTNNB1*, *TP53*, and *TERT* promoter mutations, were less frequent in FALD-associated HCCs than in other types of HCCs [10]." (Introduction, Page 6, Line 15- Page 7, Line 2)

R1: a.On Page 24, Line 1, a similar statement with the same references is included. Reference 55 also includes analysis of FALD-associated liver cancer samples, but it is transcriptomics based and makes no mention of a paucity of canonical driver gene mutations.

A: We have revised the relevant statement as follows:

"Fontan-associated liver disease (FALD) is associated with the development of HCCs characterized by lower frequencies of canonical HCC DGMs, but the mechanism underlying this association is unknown [8,10]." (Page 25, Lines 2-4)

R1: b.Page 25, Line 6 also references FALD-associated HCC as canonical DGM-negative".

A: We have modified the relevant statement as follows:

"Taken together, these results demonstrate that the Cluster 01 signature driven by the YAP1-TEAD-TET1 axis is not only activated in FALD, but can also serve as a valuable prognostic marker for established human HCCs (Fig. 7h)." (Page 26, Lines 5-8)

R1: c.Page 31, Line 4 also references a “paucity of canonical DGMs” in FALD-associated liver cancer.

A: We have revised the relevant statement as follows:

“This early onset may be one factor explaining the lower frequencies of canonical HCC DGMs reported in FALD-related HCCs [8-10].” (Page 32, Lines 10-11)

New Minor Issues

R1: 1.Page 16, Lines 13-14. The statement “These results indicate that TET1 is essential for YAP1-TEAD-induced HCC formation.” is too strong, given the supporting evidence. In the Tet1 knockdown cohort, 5 of 11 mice still developed liver tumors. If the tumors in this cohort have restored levels of Tet1, which was not assessed, the statement is justified. Otherwise, it’s clear that the activated YAP1 transgene can still drive tumor formation in the face of Tet1 knockdown, just with lower penetrance and multiplicity.

A: We have revised the relevant statement as follows:

“These results indicate that *Tet1* knockdown attenuates YAP1–TEAD-induced HCC formation, reducing tumor penetrance and multiplicity.” (Page 17, Lines 13-15)

R1: 2.Page 19, Line 3; Page 20, Line 5; Page 21, Line 18; Page 29, Line 1. It’s unclear what experimental evidence supports the conclusion that HCCs induced in this model system are derived from expansion of a single progenitor hepatocyte. Constitutively-active YAP seems to be a strong oncogenic driver, so it seems plausible that multiple hepatocytes that initially integrated the transgenic plasmid following hydrodynamic injection could clonally expand and contribute to the same tumor mass, even given the mosaic nature of the model. Clonality could be assessed by mapping insertion sites for the transgenic plasmid. I don’t think that’s necessary for this study, but without direct evidence conclusions about monoclonality can’t be made.

A: We thank the reviewer for this important comment. As the reviewer points out, we cannot conclude that the tumors were derived from a single hepatocyte. Therefore, we have removed the words “a single hepatocyte” from the revised manuscript.

R1: 3.The DGM designation should be clarified throughout the manuscript, particularly when compared to the Day 10 data. Day 10 data is from the Wu et al. study's pre-malignant samples, while 4M" data is from the current study's HCC samples. Simply referring to these data sets as Day 10 and 4M does not provide the adequate context that one is tumor and the other is non-tumor. I recommend changing 4M to 4M HCC throughout the manuscript, as was done for Fig. 5B.

A: As suggested by the reviewer, we have changed "4M" to "4M HCC" throughout the relevant parts of the revised manuscript and figures.

R1: 4.Page 26, Lines 10-12. Claims that the current model is the first mammalian model of DGM-negative carcinogenesis shown to be driven by epigenetic alterations that are induced by YAP1 activation" are undercut somewhat by Reference 37, which is accurately described on Page 28, Lines 5-10. Given that Wu et al. demonstrated YAP1 activation-driven HCC in mice that was dependent on Tet1 and associated with epigenetic alteration of Yap1 targets, some additional clarification of would be helpful. Is the statement based on the lack of detected canonical DGMs in the current study, which Wu et al. didn't assess?

A: We agree with the reviewer's interpretation. Wu et al. did not evaluate whether canonical DGMs were present or absent in their model. In contrast, our study focused on the mechanism of carcinogenesis in tumors that harbor fewer canonical DGMs. To clarify this point, we added the following sentence.

"Although Wu *et al*. previously showed that TET1-mediated DNA demethylation downstream of YAP1 activation contributes to liver cancer formation, they did not evaluate DGMs in this study of their model [38]." (Page 27, Lines 11-13)

R1: 5.Page 59, Line 8. The Fig. 4 legend should have the word "promoter" removed from this line, as the authors extended analysis beyond promoter regions for the revised manuscript.

A: As suggested by the reviewer, we have removed the word “promoter” from the revised Figure 4 legend.

Reviewer #2 (Remarks to the Author):

The authors have addressed all the comments. The quality of the manuscript was significantly improved.

We thank the reviewer for this positive evaluation and for acknowledging that our manuscript has improved in quality.

Reviewer #3 (Remarks to the Author):

The authors have provided a substantially improved manuscript. All of my concerns have been addressed sufficiently, and I now approve publication of this work.

We sincerely thank the reviewer for this positive evaluation and for approving the publication of our work.

Point-by-Point Rebuttal to the Reviewers for Manuscript # COMMSBIO-25-7657B

Editor (Remarks to the Author):

Thank you again for submitting your revised manuscript "YAP1–TEAD–TET1 axis induces hepatocellular carcinoma via DNA demethylation rather than by canonical driver gene mutations" to Communications Biology. It has now been seen again by our Reviewer #1, whose comments are appended below. As you can see, they are now recommending publication. We therefore invite you to revise your paper to comply with editorial policies and formatting requirements and to maximise the accessibility and therefore the impact of your work.

Please edit your revised manuscript according to the requests listed in the online table. Please read and complete the table, outline all revisions made in the right-hand column, and include any relevant notes that may aid us in assessing your revised work. When resubmitting, please include a clean version of your revised manuscript with no mark-ups.

We sincerely thank the Editor for the opportunity to revise our manuscript and for providing detailed editorial guidance. We have carefully addressed all requests listed in the online table.

In accordance with the editorial request to limit the title to no more than 15 words, we changed the title from "YAP1–TEAD–TET1 axis induces hepatocellular carcinoma via DNA demethylation rather than by canonical driver gene mutations" to "YAP1 induces hepatocellular carcinoma via DNA demethylation rather than by canonical driver gene mutations."

A clean version of the revised manuscript with no mark-ups has been prepared for resubmission.

Reviewer #1 (Remarks to the Author):

The authors have addressed all of the issues raised during review. Their efforts have significantly improved clarity of the manuscript and are appreciated. I recommend publication at this stage.

We sincerely thank the reviewer for the careful reassessment of our revised manuscript and for recommending publication.

In this revised manuscript, the authors have adequately addressed nearly all of the issues raised in my review of the initial submission. In some cases, however; some slight additional modifications should be made, as detailed below. Additionally, the authors have added a substantial amount of new data and discussion to the revised manuscript. Based on this new information, one major issue and 5 minor issues should be addressed prior to acceptance for publication.

Remaining Major Issues (Initial Submission)

1. Claims of tumors lacking "driver gene mutations" need to be removed, revised, or significantly qualified. This is a major theme throughout the manuscript and even included in the title but is, in my opinion, unjustified. Several reasons are provided below.
    a. The mouse model utilized to generate HCCs is driven by expression of a transgenic YAP construct that has been engineered with serine-to-alanine mutations that render it unaffected by phosphorylation-based regulatory mechanisms that would otherwise decrease its signaling activity. At a fundamental level, these tumors are essentially induced by "driver gene mutations" in the YAP transgene, which is expressed from a promoter with very high activity in the liver (Alb). As the authors state, mutations in YAP are very rare in human cancer, though activation of the pathway is fairly common. While the transgene is meant to mimic YAP-TEAD activation that can occur downstream of fibrosis or other disease states, it still represents a mutant form of YAP that is essential to the model. Notably, a wild-type YAP transgene delivered the same way did not produce tumors in any of the 22 mice listed in Table 1.

We thank the reviewer for this important comment. In this study, we used a mutant form of the oncogene YAP1, YAP1(2SA), to induce hepatocellular carcinoma (HCC) in mice. The rationale for using this system was to induce HCC within a short period (4 months). In contrast, sustained activation of endogenous YAP1–TEAD by mechanical stress (without genetic alteration) requires a prolonged period to induce HCC. Indeed, as the reviewer points out, expression of wild-type (WT) YAP1 did not induce HCC even after 12 months. Therefore, we consider that this experimental system using YAP1(2SA) represents an appropriate model to mimic the results of sustained YAP1– TEAD activation that would otherwise require a long time to induce malignancy.

I agree that the model is appropriate and justified. Transformation and tumor growth is, however, still induced by expression of a driver gene harboring an activating mutation. The authors' modifications to the text are appreciated and have sufficiently clarified and qualified conclusions in almost all sections.

There are some remaining instances where "DGM-free" should be changed to "canonical DGM-free", as listed below.

Page 13, Line 11

Page 26, Lines 3-4, 9, 11, 17
Page 30, Line 8

b. The hydrodynamic tail vein injection method used to deliver the YAP transgene results in stable incorporation of recombinant DNA into the genome of hepatocytes. Transgene integration can cause insertional mutagenesis, depending on integration sites. As integration sites were not assessed, it is possible that insertional mutagenesis could contribute to tumor formation. There is precedent for mouse tumors, including HCC, being driven by transgene-induced insertional mutation (e.g. PMID 17656716).

We agree with the reviewer on this important point. Genes introduced by hydrodynamic tail vein injection (HTVi) can be integrated into the genome, and insertional mutagenesis may occur depending on the integration sites. However, when WT YAP was introduced in this manner, no tumor formation was observed at 6 months (0/10 mice), and no tumors were detected even at 12 months (0/3 mice) (revised Fig. 1d). In contrast, when YAP1(2SA) was introduced, tumor formation was observed in all mice at 6 months (3/3 mice). Based on these results, we consider that tumor formation in this model is unlikely to be driven by insertional mutagenesis.

The delivery method depends on plasmid integration for maintained expression of the transgenic cargo. A lack of tumor formation with WT YAP constructs in 10 mice does not exclude the possibility that transgene insertions in YAP1(2SA)-injected mice produce oncogenic effects that cooperate with the transgene, which is clearly the primary driver of tumorigenesis. I'm not implying that this is likely to be the case, but it's a possibility that cannot be excluded.

Some mention of the nature of the HTVi model (i.e. genomic integration of injected plasmids) should be included somewhere in the manuscript.

2. Only protein-altering mutations were considered in the analysis depicted in Figure 3. It is unclear why this requirement was implemented when identifying "driver gene mutations". There are many examples of non-coding alterations known to be drivers of tumorigenesis (e.g. those analyzed in Reference 3).

We would like to clarify our rationale for focusing on protein-altering mutations. In our study, somatic mutation analysis was performed using whole-exome sequencing (WES). Compared with whole- genome sequencing (WGS), WES enables greater sequencing depth for a given sequencing capacity and is therefore suitable for sensitive detection of low-frequency mutations. In our analysis, sequencing was performed at a mean depth of over 330×, allowing robust evaluation of protein- altering mutations in known cancer-related genes. As the reviewer points out, non-coding alterations can contribute to tumorigenesis. However, recent large-scale pan-cancer whole-genome analyses have shown that such alterations are less frequent than protein-coding driver mutations. For

example, a comprehensive analysis of 2,658 cancer genomes (Rheinbay et al., Nature, 2020, Revised reference # 4) identified more than 1,475 driver mutations in protein-coding regions, whereas substantially fewer were identified in non-coding regions. The latter included 96 in promoters (73 in the TERT promoter), 22 in 5′ untranslated regions (5′UTRs), and 68 in 3′ untranslated regions (3′UTRs). Although non-coding regions were not directly analyzed in our study, we did not observe increased expression of TERT, a representative non- coding driver-associated gene, in the six HCCs analyzed (Supplementary Table 1). Given these considerations, we feel we are justified in focusing on protein-altering mutations in our study. Nevertheless, because we cannot exclude the possibility that non-coding alterations may have occurred, we have added the following sentences to our revised Discussion:

"Because we used short-read WES for our work, we cannot completely exclude the possibility that non-coding driver mutations or structural rearrangements may have contributed to the observed tumorigenesis. However, recent pan-cancer whole-genome analyses have shown that non-coding drivers are rare compared to protein-coding drivers 4,56,57. In a comprehensive analysis of non-coding somatic drivers in whole genomes of 2,658 cancer cells, it was predicted that more than 1,475 true driver mutations occurred in the protein-coding sequences of cancer-related genes 4. In contrast, only 96 driver mutations were estimated to have arisen in promoters (73 attributed to TERT), 22 in 5′ untranslated regions (5′UTRs), and 68 in 3′ untranslated regions (3′UTRs). None of our six HCCs showed elevated TERT expression (Supplementary Table 1), and consistent gene expression and activation of DNA demethylation targets were observed in all of our YAP1–TEAD-induced HCCs. Furthermore, TET1 knockdown suppressed tumorigenesis in our mice. Taken together, these findings indicate that HCC formation in our model is primarily mediated by epigenetic changes driven by a YAP1–TEAD-TET1 axis rather than by changes to the DNA sequence itself, including non- coding mutations or structural rearrangements."（Discussion section, Page 27, Lines 7- Page 28, Lines 4）

The authors' point is well taken. However, WES data typically includes coverage of non-coding exons (i.e. UTRs).

If that data is available, it should be included in the mutation analysis.

3. From the single-cell spatial transcriptomics analysis, four HCC-specific clusters were identified. Clusters 1-3 each expressed HCC markers, yet only Clusters 1 and 3 expressed Yap1 and its downstream targets, according to wording on page 16, lines 264-267. Does this finding suggest non-cell-autonomous effects of the YAP transgene in Cluster 2 cells? Is there an alternative interpretation for this cluster of HCC cells that don't express Yap1 and its target genes? More discussion is warranted on this topic.
    a. In the Discussion section, it is stated that "the same YAP1-TEAD target genes" are expressed across Clusters 1-4, which seems to contradict with the main text.

We apologize for this inconsistency between the main text and the Discussion. In our revised manuscript, we have corrected the description to clarify that YAP–TEAD target

gene expression was upregulated across all HCC clusters (Clusters 01–04) compared with normal hepatocyte clusters (Hepatocytes 1–6). Therefore, there is no need to discuss non-cell-autonomous effects in Cluster 02.

I was initially confused, because Figure 5f (previously 6i) shows very low *Yap1* expression in Cluster 02 and Cluster 04, which I interpreted as a lack of transgene expression. I realize now that the transgene encodes human *YAP1* (as indicated by capitalization in the manuscript and designation in the cited reference), which would generally not map to the mouse genome based on the degree of exact sequence homology. However, given that detected *Yap1* expression is lower in Cluster 02 than in the hepatocyte control, the statement on Page 20, Line 9 regarding *Yap1* expression is inaccurate.

This statement should be revised to reflect the experimental data

New Major Issue

1. In the Introduction, References 8 and 9 are used to support the statement "In FALD patients, the liver is subjected to persistent hemodynamic stress due to chronically elevated central venous pressure, leading to the development of both intrahepatic cholangiocarcinoma (ICC) and HCCs that are canonical DGM-negative". This statement is not supported by the references. Reference 8 describes molecular analysis of 5 FALD-associated liver tumors. In all 5 tumors, mutations altering canonical cancer driver genes are reported, including variants in *MAP2K2*, *NRAS*, *GNAS*, *MSH3*, *CTNNB1*, *NOTCH1*, and others. In many cases, the same genes are recurrently mutated across the small cohort. The basis for the authors' statement regarding a lack of canonical DGMs is unclear. Reference 9 is a Review article that mentions genetic/molecular profiling of FALD-associated liver cancer only by referencing Reference 8. Another reference (PMID 41587344, not cited here) performed WGS and transcriptome profiling on liver tumors from 10 FALD patients, finding rampant mutation and alteration of cancer driver genes.

    a. On Page 24, Line 1, a similar statement with the same references is included. Reference 55 also includes analysis of FALD-associated liver cancer samples, but it is transcriptomics based and makes no mention of a paucity of canonical driver gene mutations.
    b. Page 25, Line 6 also references FALD-associated HCC as "canonical DGM-negative".
    c. Page 31, Line 4 also references a "paucity of canonical DGMs" in FALD-associated liver cancer.

New Minor Issues

1. Page 16, Lines 13-14. The statement "These results indicate that TET1 is essential for YAP1-TEAD-induced HCC formation." is too strong, given the supporting evidence. In the *Tet1* knockdown cohort, 5 of 11 mice still developed liver tumors. If the tumors in this cohort have restored levels of Tet1, which was not assessed,

the statement is justified. Otherwise, it's clear that the activated YAP1 transgene can still drive tumor formation in the face of *Tet1* knockdown, just with lower penetrance and multiplicity.

2. Page 19, Line 3; Page 20, Line 5; Page 21, Line 18; Page 29, Line 1. It's unclear what experimental evidence supports the conclusion that HCCs induced in this model system are derived from expansion of a single progenitor hepatocyte. Constitutively-active YAP seems to be a strong oncogenic driver, so it seems plausible that multiple hepatocytes that initially integrated the transgenic plasmid following hydrodynamic injection could clonally expand and contribute to the same tumor mass, even given the mosaic nature of the model. Clonality could be assessed by mapping insertion sites for the transgenic plasmid. I don't think that's necessary for this study, but without direct evidence conclusions about monoclonality can't be made.

3. The "4M" designation should be clarified throughout the manuscript, particularly when compared to the "Day 10" data. "Day 10" data is from the Wu *et al.* study's pre-malignant samples, while "4M" data is from the current study's HCC samples. Simply referring to these data sets as "Day 10" and "4M" does not provide the adequate context that one is tumor and the other is non-tumor. I recommend changing "4M" to "4M_HCC" throughout the manuscript, as was done for Fig. 5B.

4. Page 26, Lines 10-12. Claims that the current model is the "first mammalian model of DGM-negative carcinogenesis shown to be driven by epigenetic alterations that are induced by YAP1 activation" are undercut somewhat by Reference 37, which is accurately described on Page 28, Lines 5-10. Given that Wu et al. demonstrated YAP1 activation-driven HCC in mice that was dependent on Tet1 and associated with epigenetic alteration of Yap1 targets, some additional clarification of would be helpful. Is the statement based on the lack of detected canonical DGMs in the current study, which Wu et al. didn't assess?

5. Page 59, Line 8. The Fig. 4 legend should have the word "promoter" removed from this line, as the authors extended analysis beyond promoter regions for the revised manuscript.

In this manuscript, the authors develop and characterize a mouse model of hepatocellular carcinoma (HCC) driven by transgenic expression of an activated form of YAP in hepatocytes. Comprehensive molecular profiling of HCCs generated with this model system is described, including whole exome sequencing, RNA-Seq, bisulfite sequencing, and single-cell spatial transcriptomics. The authors provide data supporting a model in which activated YAP leads to dysregulation of chromatin modifying and DNA methylating/demethylating machinery (most notably Tet1), leading to genome-wide effects on gene expression that promote neoplastic transformation. YAP1-TEAD targets and Tet1-demethylated gene sets are defined and shown to associate with survival outcomes in human HCC and mesothelioma cohorts. Overall, the manuscript is written in clear and concise language, and it covers an area of molecular cancer cell signaling that is timely and of broad interest to cancer researchers and clinicians. There are, however, several issues (detailed below) that should be addressed prior to acceptance for publication. Most importantly, language concerning the lack of “driver gene mutations” in patient cohorts and experimental HCCs should be softened, as it is in some cases inaccurate and in others not possible to conclude definitively based on available data. The model described is interesting and valuable in its own right but should not be described as free from driver gene mutations.

<u>Major Issues</u>

1. Claims of tumors lacking “driver gene mutations” need to be removed, revised, or significantly qualified. This is a major theme throughout the manuscript and even included in the title but is, in my opinion, unjustified. Several reasons are provided below.
    a. The mouse model utilized to generate HCCs is driven by expression of a transgenic YAP construct that has been engineered with serine-to-alanine mutations that render it unaffected by phosphorylation-based regulatory mechanisms that would otherwise decrease its signaling activity. At a fundamental level, these tumors are essentially induced by “driver gene mutations” in the YAP transgene, which is expressed from a promoter with very high activity in the liver (Alb). As the authors state, mutations in YAP are very rare in human cancer, though activation of the pathway is fairly common. While the transgene is meant to mimic YAP-TEAD activation that can occur downstream of fibrosis or other disease states, it still represents a mutant form of YAP that is essential to the model. Notably, a wild-type YAP transgene delivered the same way did not produce tumors in any of the 22 mice listed in Table 1.
    b. The hydrodynamic tail vein injection method used to deliver the YAP transgene results in stable incorporation of recombinant DNA into the genome of hepatocytes. Transgene integration can cause insertional mutagenesis, depending on integration sites. As integration sites were not assessed, it is possible that insertional mutagenesis could contribute to tumor formation. There is precedent for mouse tumors, including HCC, being driven by transgene-induced insertional mutation (e.g. PMID 17656716).

c. Although a great deal of genome-level characterization is provided for the HCC samples, it is based on short-read sequencing. No assessment of structural rearrangements is performed, so it is possible that this type of variation contributes to tumorigenesis in the model.
d. The absence of cancer-promoting genetic aberrations in tumor samples is much more difficult to definitively demonstrate than their presence. Methodologies utilized may not be able to detect certain types of mutations (e.g. short-read sequencing cannot adequately assess structural rearrangements). Sampling bias may cause mutations to be missed. The absence of <u>detectable</u> mutations in <u>known</u> driver genes, as limited by methodologies utilized and depth of analysis, is not the same as a lack of driver gene mutations in a sample.
e. References to human tumors lacking driver gene mutations are discussed in the Introduction on page 5, with two main studies cited (References 3-4). Additionally, DGM-negative cancers are described as a "phenomenon often observed in human malignancies", which is not accurate based on the data provided.
    i. Reference 3 does not make a claim that a subset of tumors analyzed lacked driver gene mutations. This interpretation by the authors of the current manuscript appears to come from the oncoplot shown in Figure 1b, which lists mutations in specific genes identified in the patient cohort and shows a group of 38 samples for which none of these mutations were detected. Supplemental Table 1b provides more comprehensive data on all 494 analyzed HCCs. Among the 38 samples for which no mutations were detected in the genes shown in the oncoplot, there are over 1,500 instances of structural variations, chromothripsis, kataegis, altered ploidy, and extrachromosomal DNA that could affect activity of cancer-promoting genes.
    ii. In Reference 4, "driver genes" are defined relatively narrowly as known promoters of lung cancer. The authors of the current manuscript highlight the finding that "19% of these tumors in never-smokers had no detectable DGMs". This number is based on data shown in Figure 2 of Reference 4, for which "driver genes" were defined as "known activating mutations or fusions in RTK/RAS/RAF pathway genes". By this same classification, 51.5% of tumors from smokers fall in the same category, and it is well accepted that smoking-associated lung cancer is driven by accumulated mutations. Data presented in Figure S2 of Reference 4 uses a broader definition of "drivers" as "LUAD-related genes", finding alterations in 94% of tumors from never-smokers. This definition is still too narrow to claim absence of driver gene mutations, and other types of mutations (e.g. structural variations) were not comprehensively assessed.

2. Only protein-altering mutations were considered in the analysis depicted in Figure 3. It is unclear why this requirement was implemented when identifying “driver gene mutations”. There are many examples of non-coding alterations known to be drivers of tumorigenesis (e.g. those analyzed in Reference 3).

3. From the single-cell spatial transcriptomics analysis, four HCC-specific clusters were identified. Clusters 1-3 each expressed HCC markers, yet only Clusters 1 and 3 expressed Yap1 and its downstream targets, according to wording on page 16, lines 264-267. Does this finding suggest non-cell-autonomous effects of the YAP transgene in Cluster 2 cells? Is there an alternative interpretation for this cluster of HCC cells that don’t express Yap1 and its target genes? More discussion is warranted on this topic.
    a. In the Discussion section, it is stated that “the same YAP1-TEAD target genes” are expressed across Clusters 1-4, which seems to contradict with the main text.

4. Figure 7a,e display p-value=0 for gene set correlations. A p-value of zero is not possible. As with other figures in the manuscript “p < x” should be used instead of p = 0.

<u>Minor Issues</u>

1. TAZ is not mentioned at all in the manuscript. YAP and TAZ function at the same mechanistic level in the Hippo signaling pathway. They are often treated as interchangeable/redundant mediators of Hippo signaling. While this characterization is an oversimplification, it would be beneficial to include some discussion of TAZ, at least in the Introduction and/or Discussion section. Is it expected that transgenic expression of active TAZ would produce a similar model of HCC?

2. In Table 1, several constructs are shown that are not described in the manuscript. In particular, the 5SA/WW1,2* construct (which according to Reference 37 is defective in binding transcription factors, such as p73) drives robust HCC formation in all mice analyzed. What is the interpretation of this result? Any of the constructs shown in Table 1 should be discussed in the text of the manuscript.

3. Page 10, lines 151-153: The authors discuss significantly upregulated and downregulated genes identified by RNA-Seq. It would be valuable to perform gene set enrichment analysis (GSEA) to determine whether YAP-TEAD target gene sets are significantly enriched in tumors versus normal liver samples. Several such gene sets have been described (e.g. in PMID 38473214).

4. Did the authors analyze RNA-Seq data for altered splicing or RNA editing in HCCs versus normal liver samples? This data could be informative.

5. Figures 4b and 5e show data from 11 HCCs and 10 non-tumor samples, while Figure 2e-g only shows the 6 HCCs and 5 non-tumor samples that were the focus of most of the manuscript. It is unclear which additional samples were utilized for the analyses in Figures 4-5.

6. Page 14, Line 222: It is unclear what is meant by a fold-change of ≤1/1.5.

7. Page 16, Lines 260-262: Do any of the gene expression signatures identified in HCC clusters 1-4 overlap with findings from the bulk sequencing analysis?

8. In Figure 1c-d, it is unclear whether an anti-YAP antibody (as used in Figure 1c) or an anti-Myc antibody was used for immunofluorescence. This point is important, as it determines whether total YAP or only transgenic YAP is detected.

9. In Figure 6m, it would improve clarity if a border could be drawn around the portion of the tissue section that is histologically HCC.

10. Page 18, lines 301-303: The authors suggest that the gene expression signature may be applicable to additional cancer types. It would be simple to do similar assessments as shown in Figure 7 on additional TCGA datasets.

11. Page 19, Lines 307-308: The statement "YAP1 was introduced into single hepatocytes, followed by mosaic analysis" is strangely phrased. Something like "YAP1 was introduced mosaically into hepatocytes" would be more clear.
