# Supplementary material for: YAP1 induces hepatocellular carcinoma via DNA demethylation rather than by canonical driver gene mutations
Source: Commun Biol. 2026 Sep 17;9:1198. doi: 10.1038/s42003-026-10882-w (PMC13586348; doi:10.1038/s42003-026-10882-w)
Supplement: Supplementary file 2 [file 42003_2026_10882_MOESM26_ESM.pdf]

nature portfolio

Corresponding author(s): Yoshimi Okamoto-Uchida, Teppei Shimamura, Hiroshi Nishina

Last updated by author(s): June 30, 2026

Reporting Summary

Nature Portfolio wishes to improve the reproducibility of the work that we publish. This form provides structure for consistency and transparency in reporting. For further information on Nature Portfolio policies, see our Editorial Policies and the Editorial Policy Checklist.

Statistics

For all statistical analyses, confirm that the following items are present in the figure legend, table legend, main text, or Methods section.

n/a	Confirmed	
☐	☒	The exact sample size (*n*) for each experimental group/condition, given as a discrete number and unit of measurement
☐	☒	A statement on whether measurements were taken from distinct samples or whether the same sample was measured repeatedly
☐	☒	The statistical test(s) used AND whether they are one- or two-sided<br>*Only common tests should be described solely by name; describe more complex techniques in the Methods section.*
☐	☒	A description of all covariates tested
☐	☒	A description of any assumptions or corrections, such as tests of normality and adjustment for multiple comparisons
☐	☒	A full description of the statistical parameters including central tendency (e.g. means) or other basic estimates (e.g. regression coefficient) AND variation (e.g. standard deviation) or associated estimates of uncertainty (e.g. confidence intervals)
☐	☒	For null hypothesis testing, the test statistic (e.g. *F*, *t*, *r*) with confidence intervals, effect sizes, degrees of freedom and *P* value noted<br>*Give P values as exact values whenever suitable.*
☒	☐	For Bayesian analysis, information on the choice of priors and Markov chain Monte Carlo settings
☒	☐	For hierarchical and complex designs, identification of the appropriate level for tests and full reporting of outcomes
☐	☒	Estimates of effect sizes (e.g. Cohen's *d*, Pearson's *r*), indicating how they were calculated

Our web collection on statistics for biologists contains articles on many of the points above.

Software and code

Policy information about availability of computer code

Data collection	BZ-X710 fluorescence microscope (Keyence, Osaka, Japan) NovaSeq 6000 plus NovaSeq Control Software v1.6.0, Real Time Analysis (RTA) v3.4.4, Bcl2fastq2 v2.20 (Illumina, San Diego, CA, USA) ChemiDoc MP system (Bio-Rad, Hercules, CA, USA) AVITI platform (Element Biosciences, San Diego, CA, USA)
Data analysis	ImageJ software (v1.54g, National Institutes of Health, Bethesda, MD, USA) Integrative Genomics Viewer (IGV, v2.18.0, Broad Institute, Cambridge, MA, USA) DRAGEN Bio-IT Platform v3.6.3 (Illumina, San Diego, CA, USA) iDEP (v1.1, South Dakota State University, Brookings, SD, USA) R (v4.4.2) Python (v3.12.3) with libraries: gseapy (v1.1.8) , seaborn (v0.13.2) , Matplotlib (v3.9.2) , pandas (v2.2.2) Trim Galore (Babraham Institute, Cambridge, UK) Bismark (v0.23.0, Babraham Institute, Cambridge, UK) Space Ranger (10x Genomics, Pleasanton, CA, USA) Cellpose (v3.1.1.1, Janelia Research Campus, Ashburn, VA, USA) scVI model (v1.3.0, University of California, Berkeley, CA, USA) GraphPad Prism 8 (GraphPad Software, San Diego, CA, USA) GEPIA2 (http://gepia2.cancer-pku.cn)

For manuscripts utilizing custom algorithms or software that are central to the research but not yet described in published literature, software must be made available to editors and reviewers. We strongly encourage code deposition in a community repository (e.g. GitHub). See the Nature Portfolio guidelines for submitting code & software for further information.

Data

Policy information about availability of data

All manuscripts must include a data availability statement. This statement should provide the following information, where applicable:

- Accession codes, unique identifiers, or web links for publicly available datasets
- A description of any restrictions on data availability
- For clinical datasets or third party data, please ensure that the statement adheres to our policy

All high-throughput sequencing data generated in this study have been deposited in the DDBJ Sequence Read Archive (DRA) under BioProject accession number PRJDB35548. The Visium HD data have been deposited in the Genomic Expression Archive (GEA) under GEA Accession ID E-GEAD-1095. Data corresponding to the processed RNA-seq, RRBS, and WES analyses are also provided as supplementary tables.Numerical source data for graphs and charts are provided in Supplementary Data.

Research involving human participants, their data, or biological material

Policy information about studies with human participants or human data. See also policy information about sex, gender (identity/presentation), and sexual orientation and race, ethnicity and racism.

Reporting on sex and gender	Not applicable. The analysis of public TCGA data was not stratified by sex or gender.
Reporting on race, ethnicity, or other socially relevant groupings	Not applicable. The analysis of public TCGA data was not stratified by race or ethnicity.
Population characteristics	The analysis was performed using public TCGA liver cancer datasets and published single-cell RNA-seq datasets from Fontan-associated liver disease (FALD) livers. Demographic characteristics were not included as variables in the analyses.
Recruitment	Not applicable. No participants were recruited for this study as the analysis used publicly available data.
Ethics oversight	Not applicable to this study. The human data used were obtained from the publicly available database, which was collected by a third party with its own institutional review board approval.

Note that full information on the approval of the study protocol must also be provided in the manuscript.

Field-specific reporting

Please select the one below that is the best fit for your research. If you are not sure, read the appropriate sections before making your selection.

☒ Life sciences ☐ Behavioural & social sciences ☐ Ecological, evolutionary & environmental sciences

For a reference copy of the document with all sections, see nature.com/documents/nr-reporting-summary-flat.pdf

Life sciences study design

All studies must disclose on these points even when the disclosure is negative.

Sample size	Sample size was determined to be sufficient to assess statistical significance for each experiment. Specific sample sizes (e.g., n=3, n=6, n=11) are described in the legend of each figure.
Data exclusions	No data were excluded from the analyses.
Replication	All major findings were confirmed through multiple biological replicates, as detailed in the figure legends.
Randomization	Due to the nature of the study design (e.g., comparison of tumor and non-tumor tissues within the same individual), randomization of animals was not applicable.
Blinding	During experiments and data analysis, investigators were not blinded to group allocation. As objective quantitative methods such as sequencing data analysis were primarily used, the lack of blinding is unlikely to have influenced the results.

Reporting for specific materials, systems and methods

We require information from authors about some types of materials, experimental systems and methods used in many studies. Here, indicate whether each material, system or method listed is relevant to your study. If you are not sure if a list item applies to your research, read the appropriate section before selecting a response.

Materials & experimental systems

n/a	Involved in the study
☐	☒ Antibodies
☒	☐ Eukaryotic cell lines
☒	☐ Palaeontology and archaeology
☐	☒ Animals and other organisms
☐	☒ Clinical data
☒	☐ Dual use research of concern
☒	☐ Plants

Methods

n/a	Involved in the study
☒	ChIP-seq
☒	☐ Flow cytometry
☒	☐ MRI-based neuroimaging

Antibodies

Antibodies used	Western blotting: anti-Tet1 (1:500; Abcam, ab191698), anti-GAPDH (1:5,000; Millipore, MAB374).A complete list of antibodies used for immunohistochemistry (IHC) and immunofluorescence (IF) is provided in Supplementary Table S21.
Validation	The antibodies used in this study are commercially available from reputable manufacturers, and their catalog numbers are provided. The antibodies were used according to the manufacturer's protocols at the dilutions specified in the Methods section and Supplementary Table S21.

Animals and other research organisms

Policy information about studies involving animals; ARRIVE guidelines recommended for reporting animal research, and Sex and Gender in Research

Laboratory animals	Male C57BL/6J mice were used. The mice were purchased from Sankyo Labo Service Corporation and housed under specific-pathogen-free conditions. The experimental duration was approximately 4 to 12 months for the YAP1-induced HCC development model.
Wild animals	Wild animals were not used in this study.
Reporting on sex	Male mice were predominantly used in the HCC induction experiments. This was because a sex difference was observed in preliminary experiments, where female mice had a lower incidence of developing liver cancer even when the same plasmid was introduced.
Field-collected samples	Field-collected samples were not used in this study.
Ethics oversight	All mouse experiments were conducted with the approval of the Animal Care and Use Committee of the Institute of Science Tokyo.

Note that full information on the approval of the study protocol must also be provided in the manuscript.

Clinical data

Policy information about clinical studies

All manuscripts should comply with the ICMJE guidelines for publication of clinical research and a completed CONSORT checklist must be included with all submissions.

Clinical trial registration	This study is not a clinical trial; therefore, this is not applicable.
Study protocol	The expression of the YAP1-TEAD-TET1 axis and DNA demethylation-related genes was evaluated in public TCGA liver cancer datasets and FALD single-cell RNA-seq datasets.
Data collection	Publicly available TCGA liver cancer datasets and published single-cell RNA-seq datasets from Fontan-associated liver disease (FALD) livers (GSE223843) were re-analyzed in this study.
Outcomes	The expression of the YAP1-TEAD-TET1 axis and DNA demethylation-related genes was evaluated in public TCGA liver cancer datasets and FALD single-cell RNA-seq datasets, together with their association with patient survival.

Plants

Seed stocks	NA
Novel plant genotypes	NA
Authentication	NA

ChIP-seq

Data deposition

- [ ] Confirm that both raw and final processed data have been deposited in a public database such as GEO.
- [ ] Confirm that you have deposited or provided access to graph files (e.g. BED files) for the called peaks.

Data access links<br>*May remain private before publication.*	NA
Files in database submission	NA
Genome browser session<br>(e.g. UCSC)	NA

Methodology

Replicates	NA
Sequencing depth	NA
Antibodies	NA
Peak calling parameters	NA
Data quality	NA
Software	NA
