# Supplementary material for: YAP1 induces hepatocellular carcinoma via DNA demethylation rather than by canonical driver gene mutations
Source: Commun Biol. 2026 Sep 17;9:1198. doi: 10.1038/s42003-026-10882-w (PMC13586348; doi:10.1038/s42003-026-10882-w)
Supplement: Supplementary file 3 [file 42003_2026_10882_MOESM2_ESM.pdf]

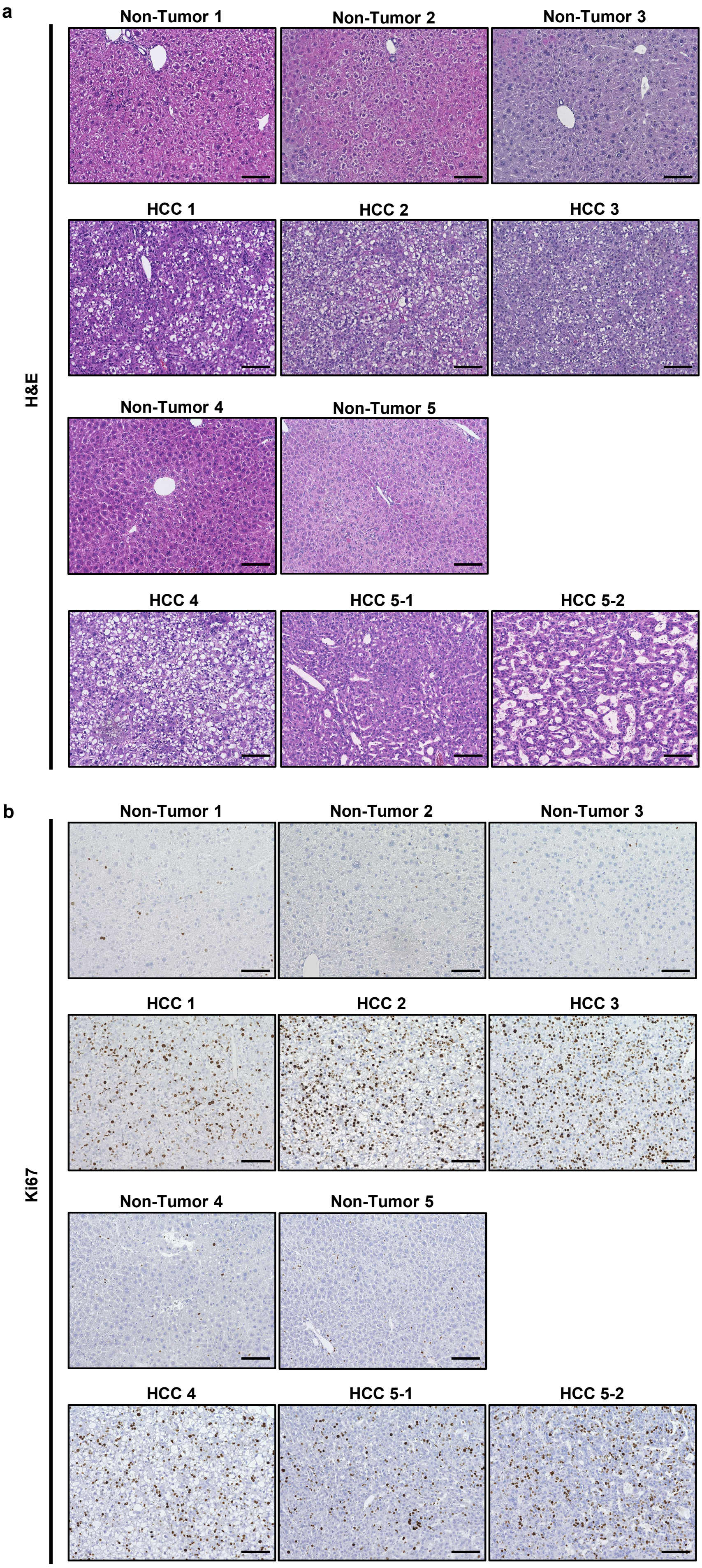

Supplementary Figure 1

Supplementary Figure 1. Histological staining of YAP1-TEAD-induced HCCs and adjacent non-tumor tissues.

(a) H&E staining and **(b)** anti-Ki67 staining of the six YAP1-TEAD-induced HCCs and non-tumor regions shown in Figure 1f. Scale bars, 100 μm.

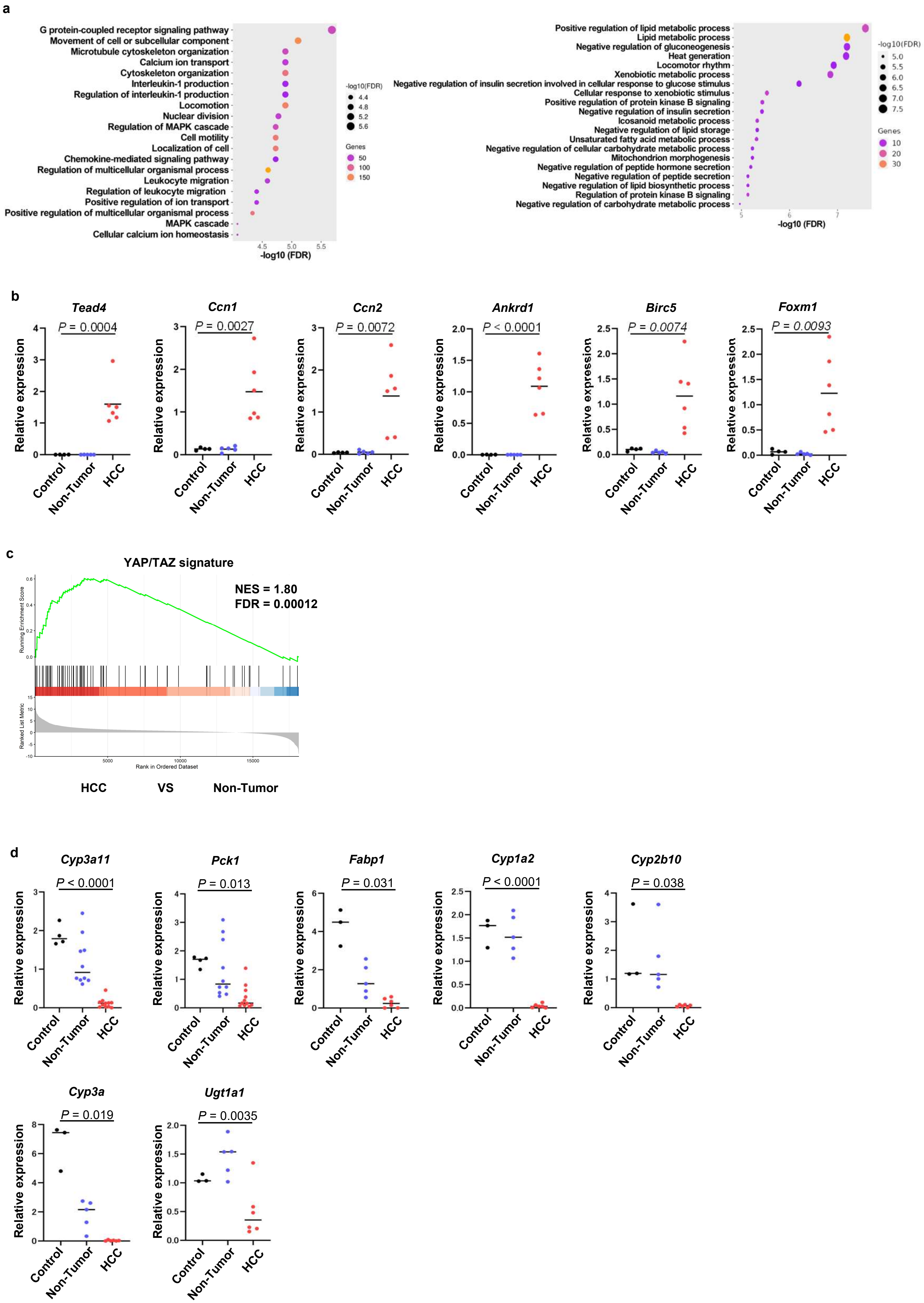

Supplementary Figure 2

Supplementary Figure 2. Gene expression analysis of six pairs of YAP1-TEAD-induced HCCs and adjacent non-tumor tissues.

(a) Reactome pathway analysis of significantly upregulated (left) and downregulated (right) gene sets in the HCCs in Figure 1f. **(b)** Relative mRNA levels of the indicated YAP1-upregulated genes in the tissues in Fig. 1h as determined by qPCR (n=6). **(c)** GSEA of the YAP/TAZ gene expression signature in YAP1-TEAD-induced HCCs compared with adjacent non-tumor tissues. **(d)** Relative mRNA levels of the indicated YAP1-downregulated genes in the tissues in Fig. 1h as determined by qPCR (n=6).

a

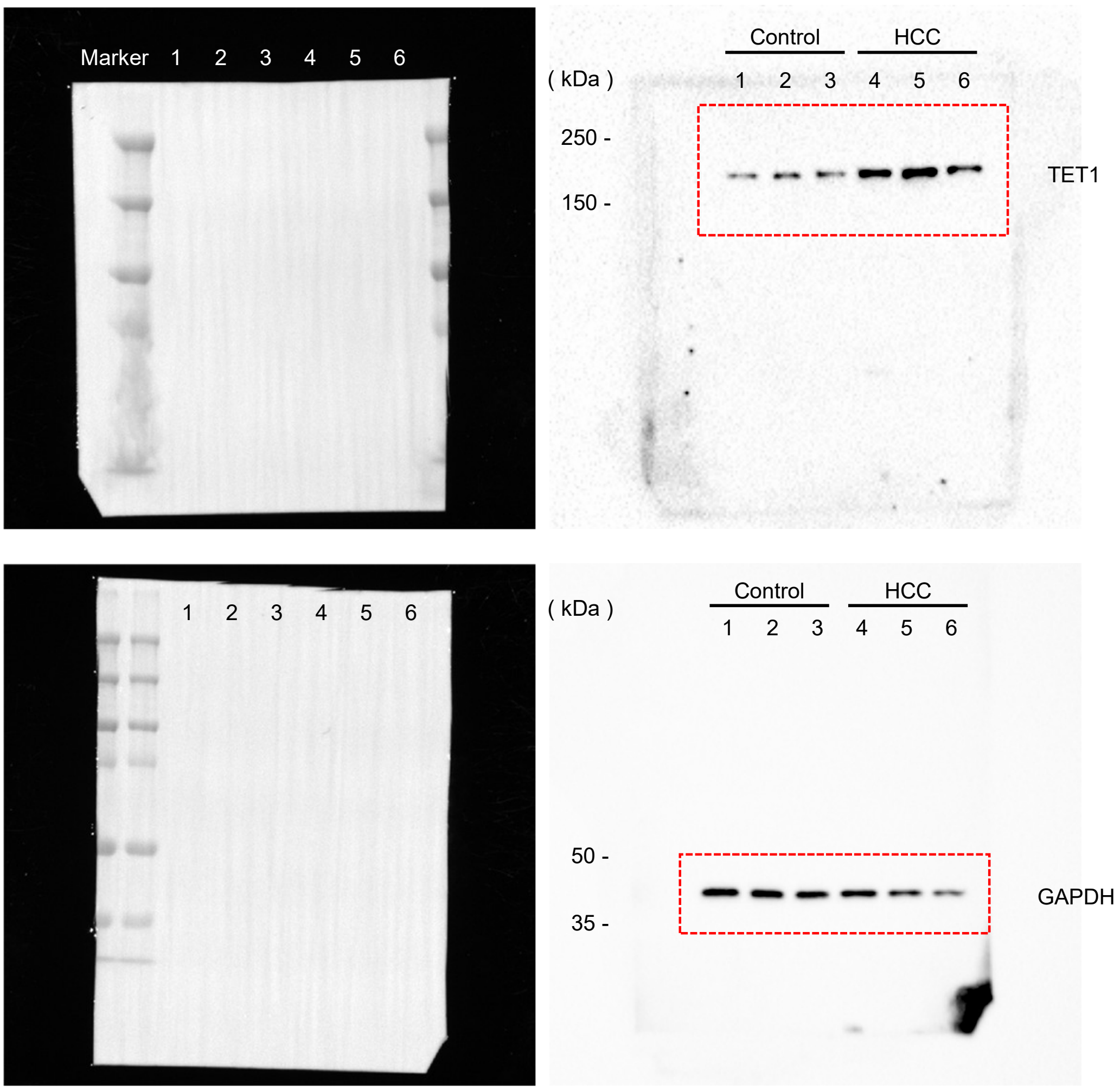

b

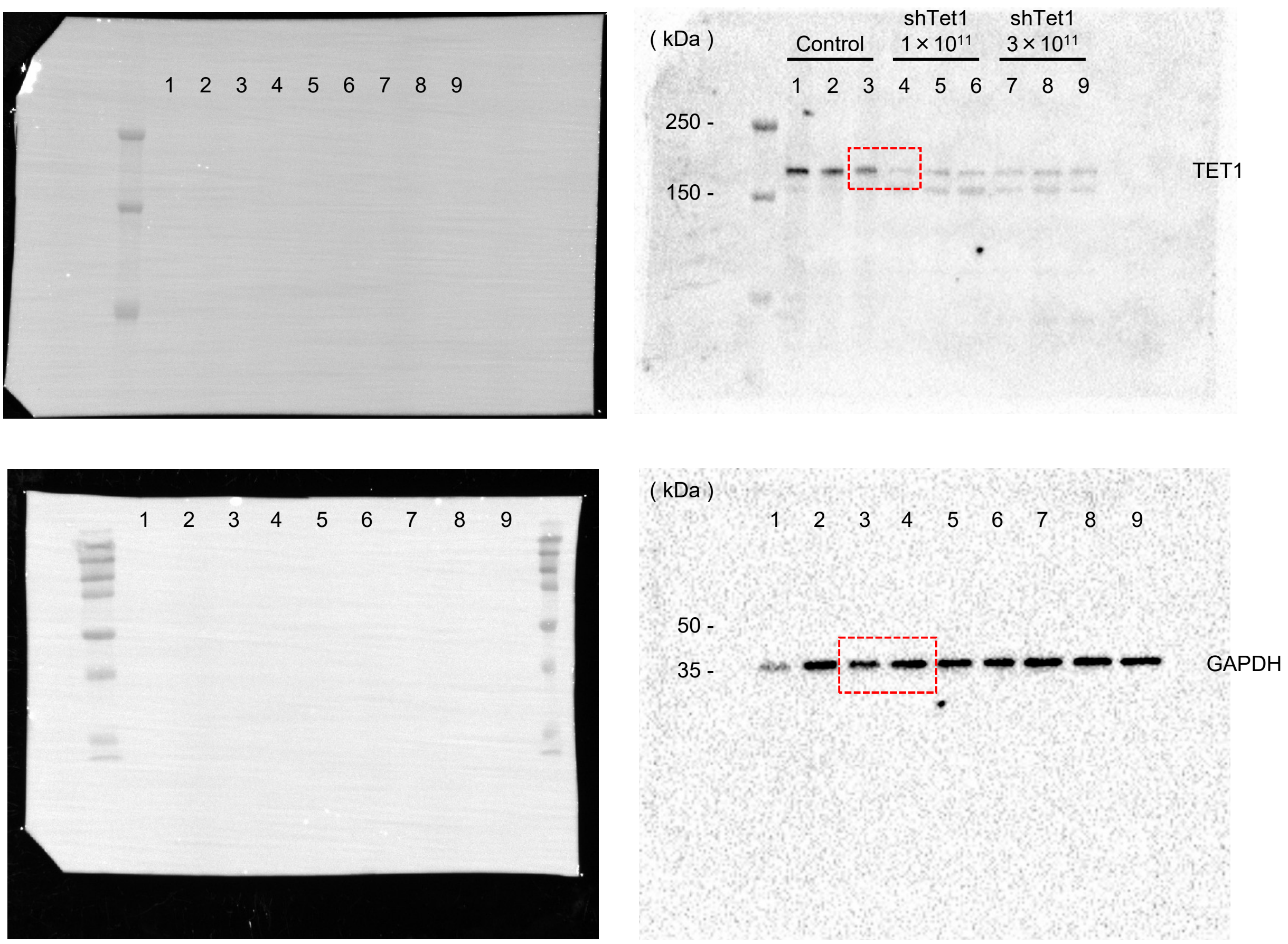

Supplementary Figure 3

Supplementary Figure 3. Raw, untrimmed Western blot data for Figure 3.

(a, b) Complete unprocessed immunoblots for **(a)** the experiment presented in Figure 3c and **(b)** the experiment presented in Figure 3d. Top: Full scan of each immunoblot for TET1. Bottom: Full scan of each immunoblot for GAPDH.

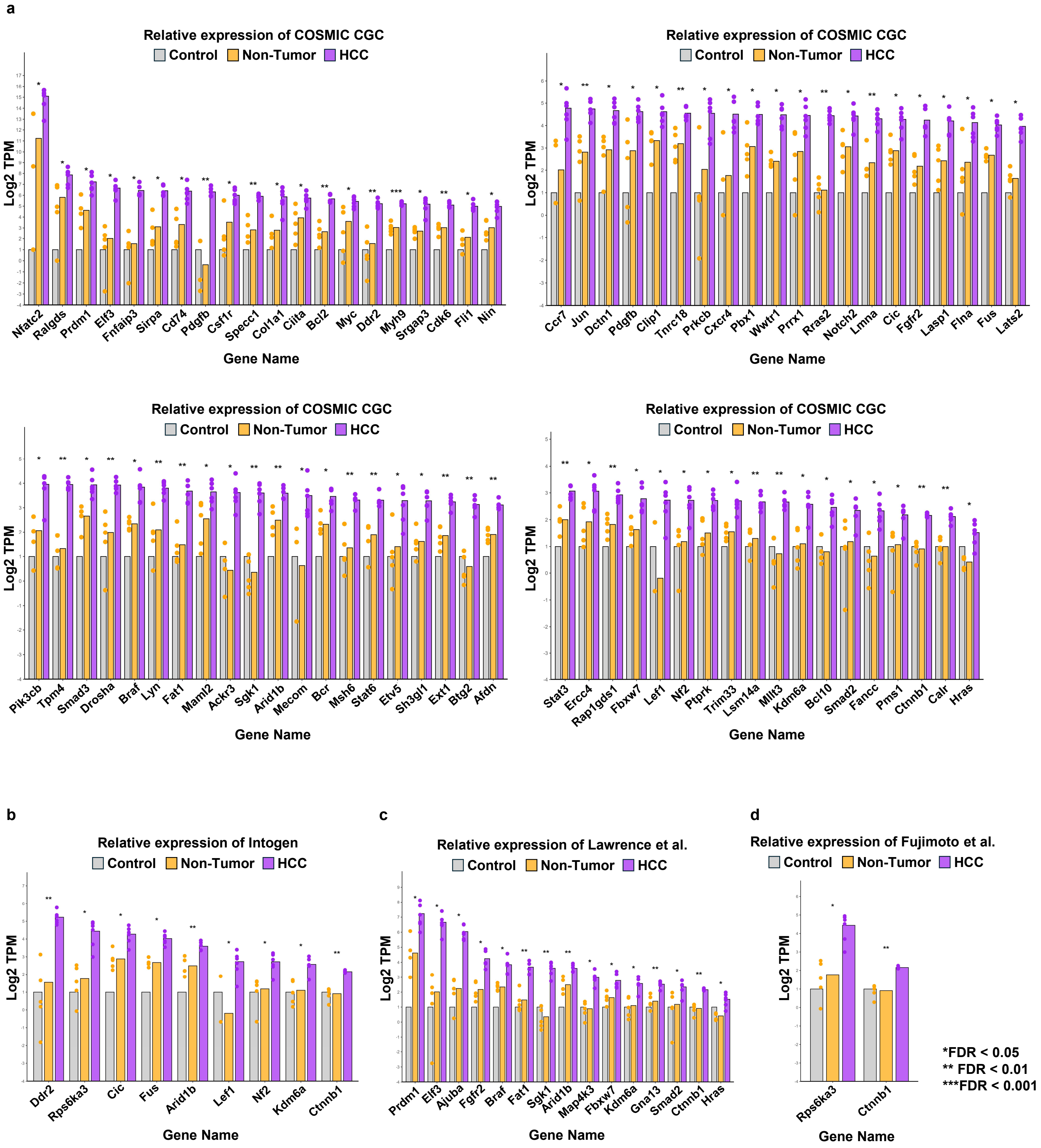

Supplementary Figure 4

Supplementary Figure 4. Expression analysis of YAP1-TEAD-TET1 target oncogenes.

(a-d) RNA-seq results for driver genes that were included in the **(a)** COSMIC CGC, **(b)** IntOgen, **(c)** Lawrence *et al.,* and **(d)** Fujimoto *et al.* datasets [45-48] and were identified among the demethylated and upregulated genes shown in Fig. 4h for YAP1-TEAD-induced HCCs (n=6).

GO Pathway Enrichment (Top20)

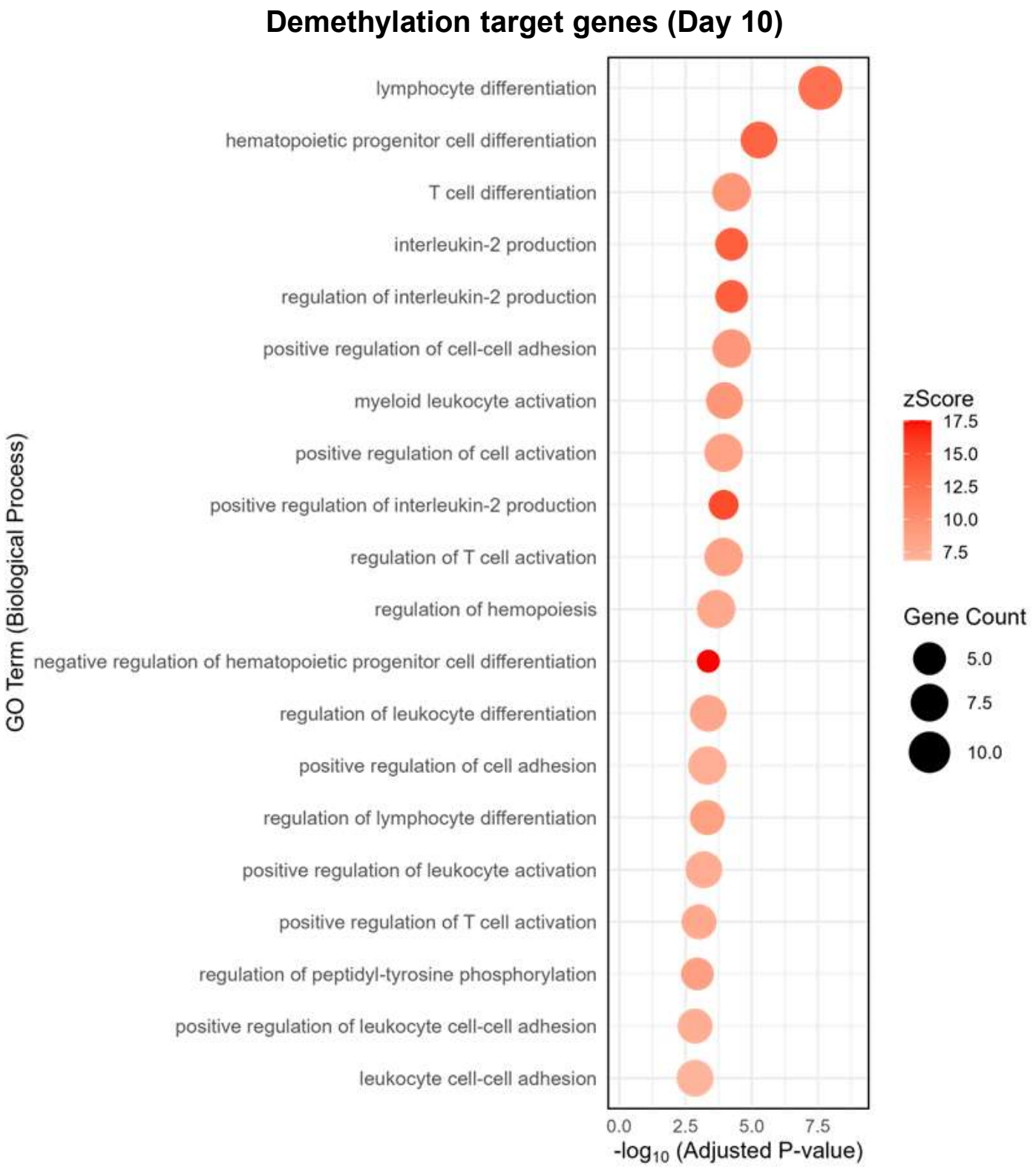

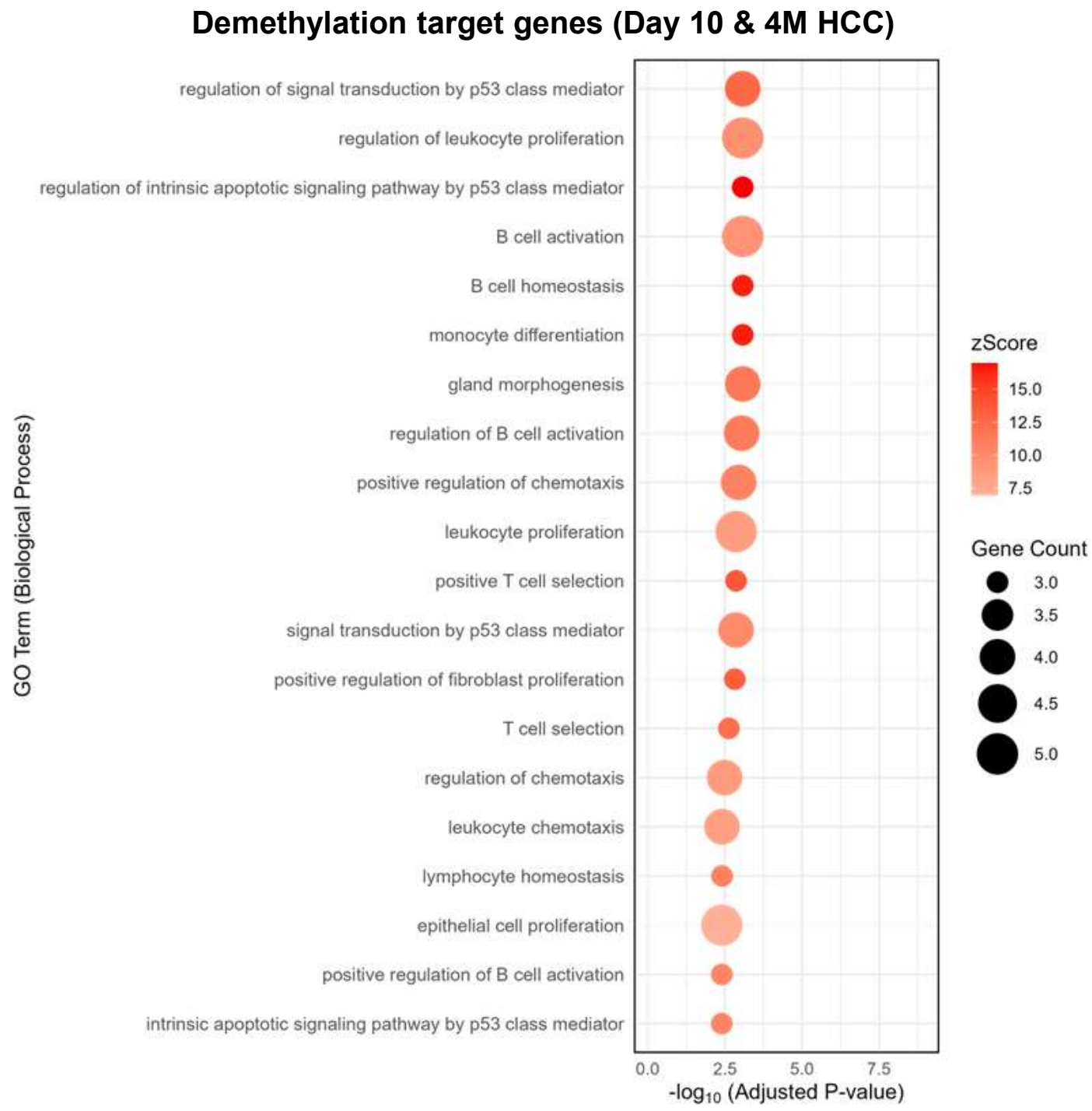

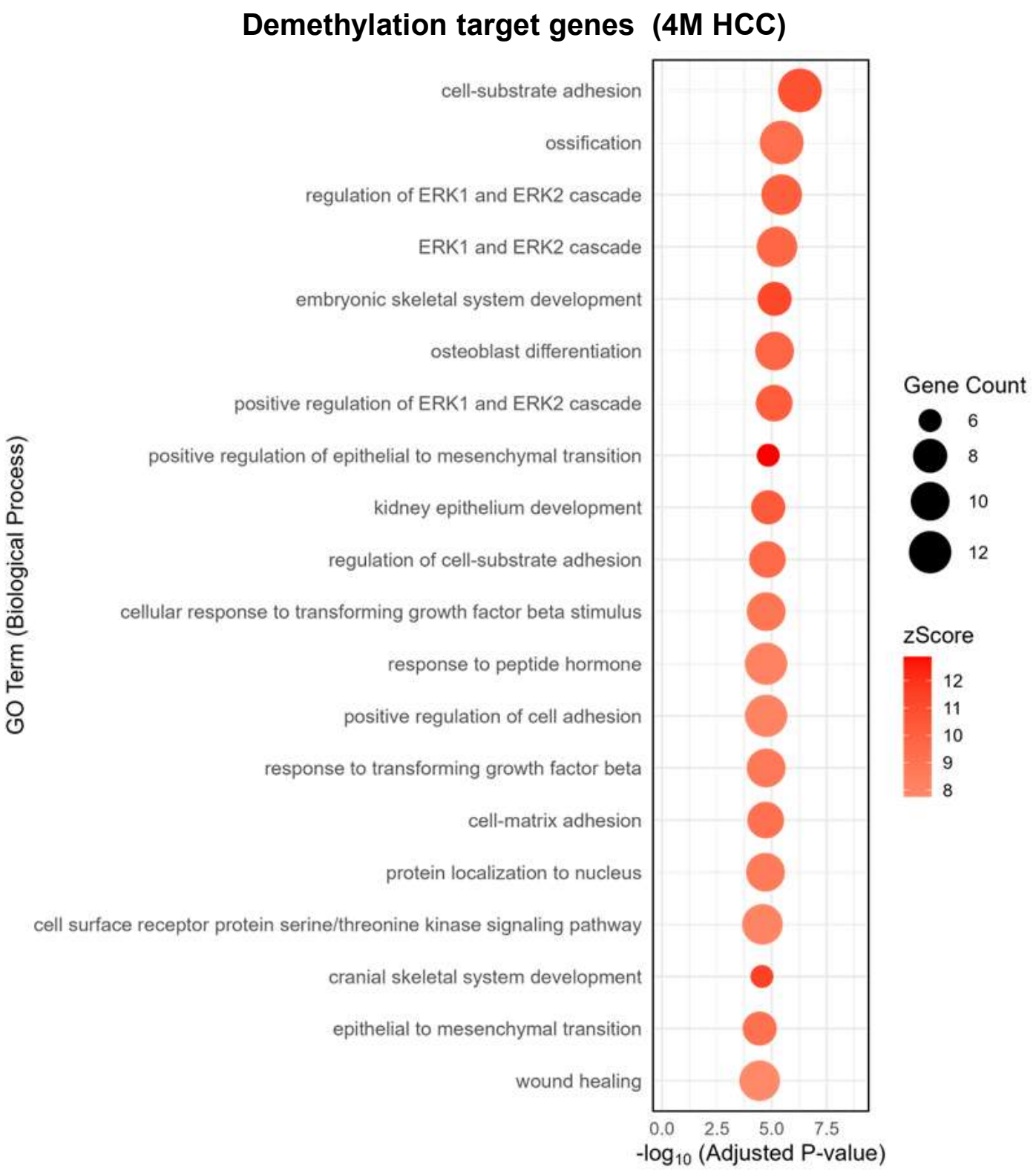

Supplementary Figure 5

Supplementary Figure 5. GO analysis of YAP1-TEAD-TET1 target oncogenes.

Dot plots showing GO pathway enrichment analysis results for oncogenes that were identified in Fig. 4h as being specific to Day 10, Day 10 & 4M HCC or 4M HCC stage liver tissues.

a

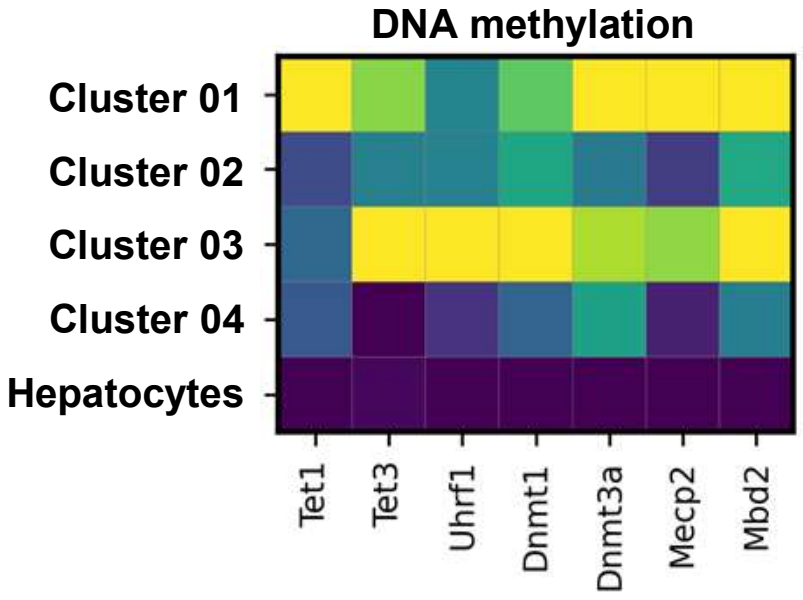

b

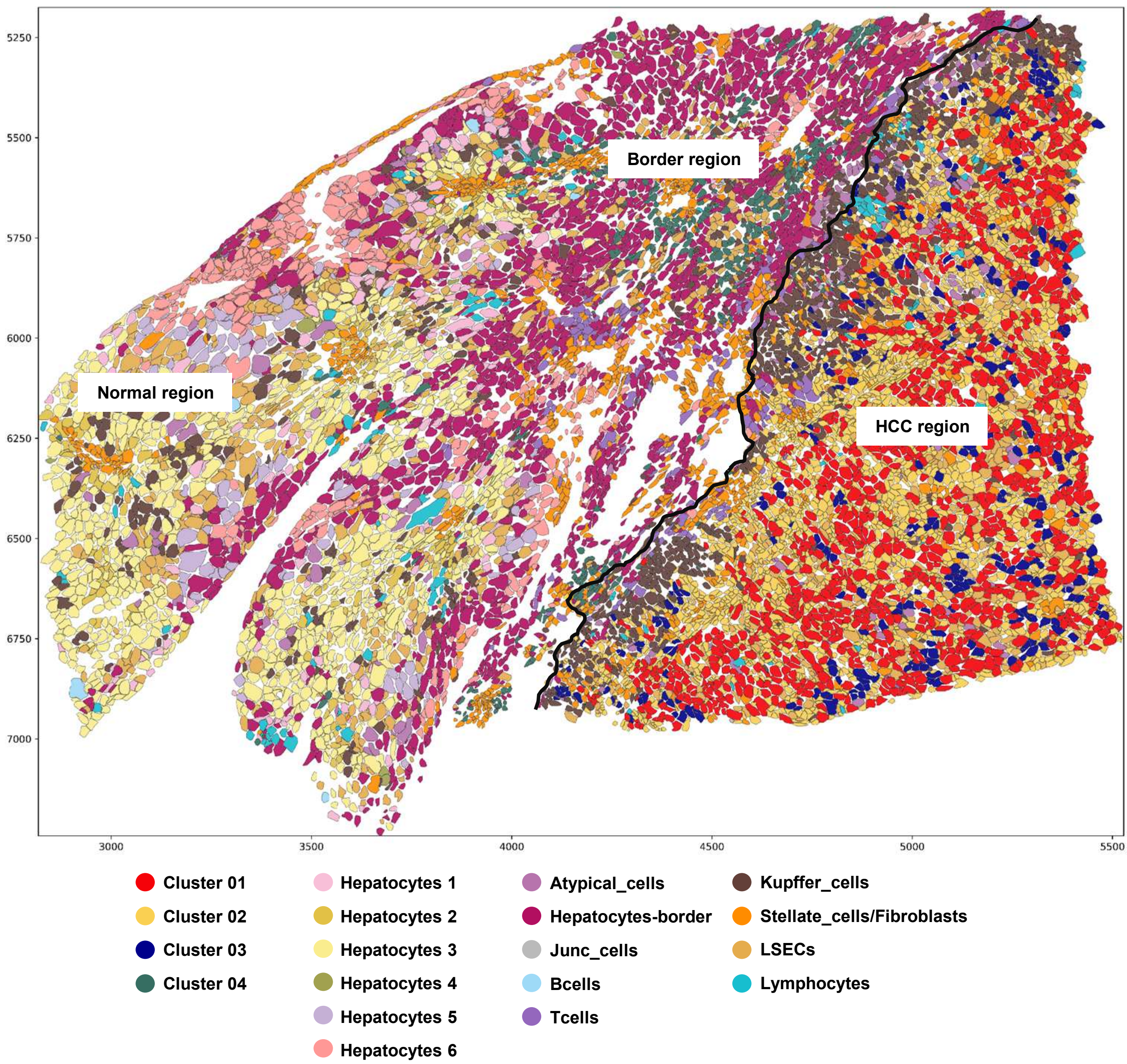

Supplementary Figure 6

Supplementary Fig. 6. Histological annotation of the spatial map shown in Figure 5m, with supplementary matrix plot analysis of DNA methylation regulatory genes. (a) Matrix plot analysis of the indicated DNA methylation regulatory genes in HCC Cluster 01, Cluster 02, Cluster 03, and Cluster 04, and in normal hepatocyte Clusters 01–06 (Hepatocytes). **(b)** Spatial distribution of the indicated cell populations (by color) in YAP1-TEAD-induced HCC and surrounding tissues, as shown in Figure 5m, with an added boundary line indicating the histologically defined HCC and non-HCC regions.

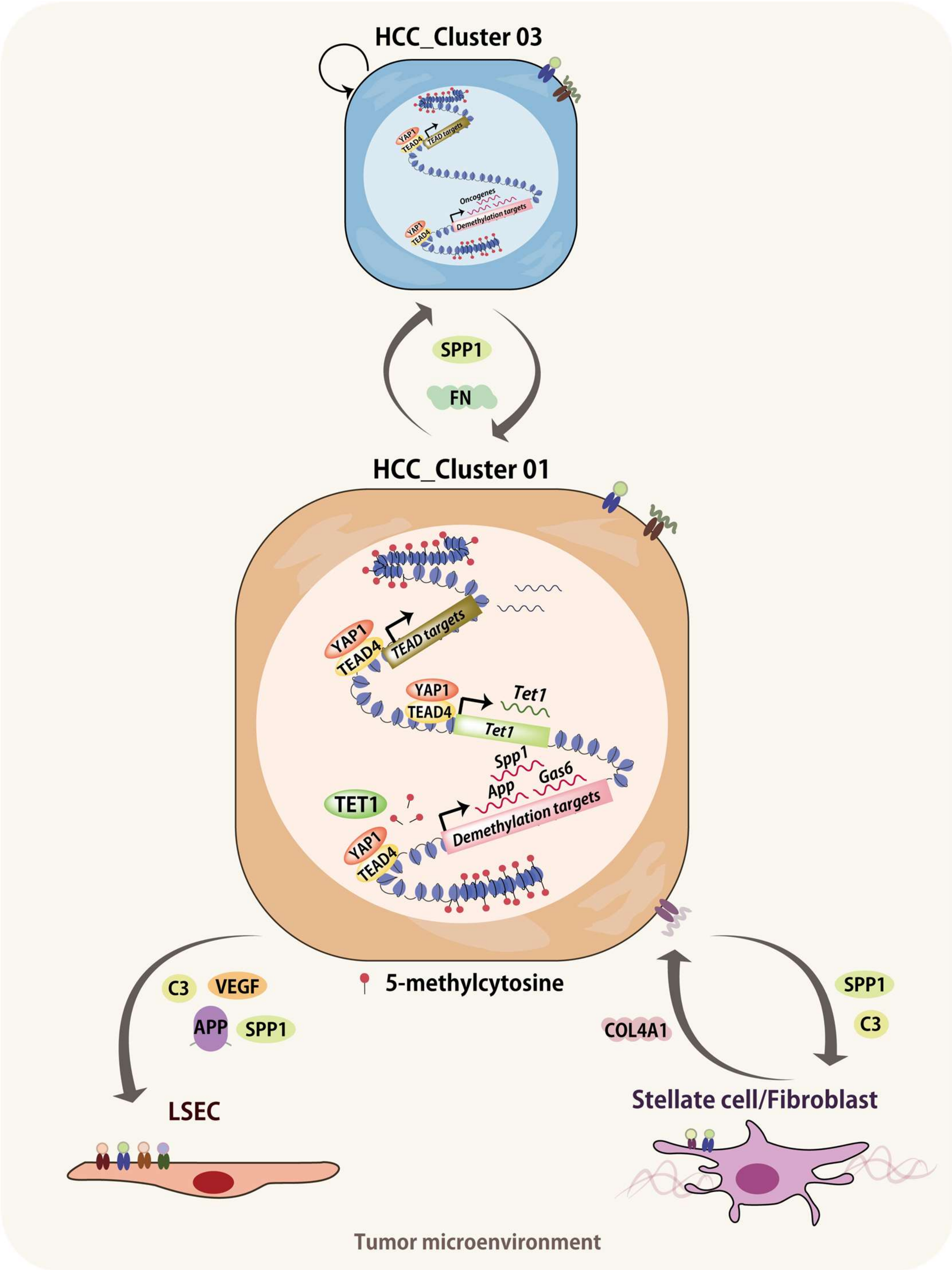

Supplementary Figure 7

Supplementary Figure 7. Schematic model of the HCC tumor microenvironment.

The diagram shows the interaction networks between HCC clusters (01 and 03) and surrounding stromal cells (LSECs and HSCs) predicted by CellChat. In HCC Cluster 01, activation of the YAP1–TEAD4–TET1 axis induces DNA demethylation and expression of demethylation target genes, including secreted factors such as SPP1 and APP. HCC Clusters 01 and 03 may mutually sustain their activation states through SPP1- and FN-mediated signaling. HCC cells, particularly Cluster 01 cells, may also remodel the tumor microenvironment through secretion of factors such as C3, VEGF, APP, and SPP1, thereby activating LSECs and stellate cells/fibroblasts. Activated stellate cells/fibroblasts may in turn promote HCC cell activation through COL4A1 signaling.

a

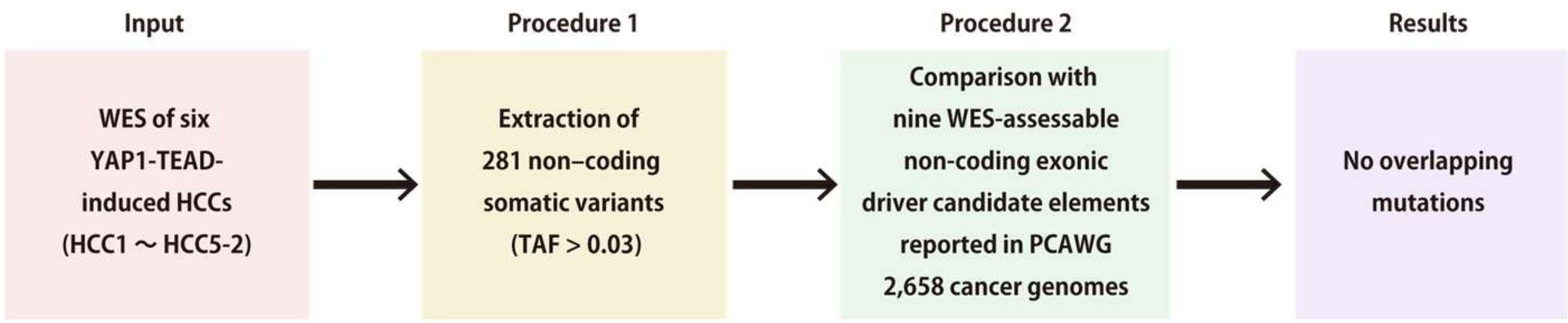

b

	HCC1	HCC2	HCC3	HCC4	HCC5-1	HCC5-2
TP53/TRP53 5' region	0	0	0	0	0	0
WDR74 5'UTR	0	0	0	0	0	0
PTDSS1/MTERF3 5'UTR	0	0	0	0	0	0
DTL 5'UTR	0	0	0	0	0	0
MTG2 5'UTR	0	0	0	0	0	0
ALB 3'UTR	0	0	0	0	0	0
TOB1 3'UTR	0	0	0	0	0	0
NFKBIZ 3'UTR	0	0	0	0	0	0
FOXA1 3'UTR	0	0	0	0	0	0

Supplementary Figure 8

Supplementary Figure 8. Analysis of non-coding somatic variants detected by WES.

(a) Workflow for comparing 281 non-coding somatic variants detected by WES in six YAP1–TEAD-induced HCCs with nine WES-assessable non-coding exonic driver candidate elements reported in the PCAWG analysis of 2,658 cancer genomes [4]. **(b)** Matrix summarizing the overlap analysis between the six HCC samples analyzed in this study and the nine WES-assessable non-coding exonic driver candidate elements in (a).
