# Supplementary material for: YAP1 induces hepatocellular carcinoma via DNA demethylation rather than by canonical driver gene mutations
Source: Commun Biol. 2026 Sep 17;9:1198. doi: 10.1038/s42003-026-10882-w (PMC13586348; doi:10.1038/s42003-026-10882-w)
Supplement: Supplementary file 4 [file 42003_2026_10882_MOESM3_ESM.pdf]

Description of Additional Supplementary files

Supplementary Data 1. RNA-seq data comparing Control, Non-Tumor, and HCC samples.

Supplementary Data 2. Tumor-specific somatic mutations detected using the DRAGEN Bio-IT Platform in whole-exome sequencing data from Non-Tumor_1 and HCC_1.

Supplementary Data 3. Tumor-specific somatic mutations detected using the DRAGEN Bio-IT Platform in whole-exome sequencing data from Non-Tumor_2 and HCC_2.

Supplementary Data 4. Tumor-specific somatic mutations detected using the DRAGEN Bio-IT Platform in whole-exome sequencing data from Non-Tumor_3 and HCC_3.

Supplementary Data 5. Tumor-specific somatic mutations detected using the DRAGEN Bio-IT Platform in whole-exome sequencing data from Non-Tumor_4 and HCC_4.

Supplementary Data 6. Tumor-specific somatic mutations detected using the DRAGEN Bio-IT Platform in whole-exome sequencing data from Non-Tumor_5 and HCC_5_1.

Supplementary Data 7. Tumor-specific somatic mutations detected using the DRAGEN Bio-IT Platform in whole-exome sequencing data from Non-Tumor_5 and HCC_5_2.

Supplementary Data 8. PubMed-based validation of cancer-related genes harboring somatic mutations identified by whole-exome sequencing in YAP1–TEAD-induced HCCs.

Supplementary Data 9. Target genes of the YAP1–TEAD–TET1 axis in YAP1-induced HCCs.

Supplementary Data 10. Comparison of YAP1–TET1 target genes between the Day 10 and HCC stages.

Supplementary Data 11. Gene Ontology analysis of Day 10-specific YAP1–TET1 target genes.

Supplementary Data 12. Gene Ontology analysis of YAP1–TET1 target genes common to the Day 10 and HCC stages.

Supplementary Data 13. Gene Ontology analysis of HCC-specific YAP1–TET1 target genes.

Supplementary Data 14. Comparison of upregulated genes targeted by DNA demethylation with four lists of known cancer driver genes.

Supplementary Data 15. Sequencing depth and saturation of the Visium HD analysis.

Supplementary Data 16. Demethylated oncogenes in Clusters 01–04.

Supplementary Data 17. Ligand–receptor pairs in the Secreted Signaling category identified by CellChat analysis.

Supplementary Data 18. Ligand–receptor pairs in the ECM–Receptor category identified by CellChat analysis.

Supplementary Data 19. Ligand–receptor pairs in the Cell–Cell Contact category identified by CellChat analysis.

Supplementary Data 20. Target sequences of shRNAs used for Tet1 knockdown.

Supplementary Data 21. List of antibodies used in this study.

Supplementary Data 22. List of primers used in this study.
